# **EPMA World Congress: Traditional Forum in Predictive, Preventive and Personalised Medicine for Multi-Professional Consideration and Consolidation**

**DOI:** 10.1007/s13167-017-0108-4

**Published:** 2017-08-14

Olga Golubnitschaja^1,2,3,4^ , Vincenzo Costigliola^,1,5^ and Godfrey Grech^1,6^

^1^European Association for Predictive, Preventive and Personalised Medicine, EPMA, Brussels, Belgium

^2^Radiological Clinic, Rheinische Friedrich-Wilhelms-Universität Bonn, Sigmund-Freud-Str 25, 53105 Bonn, Germany

^3^Breast Cancer Research Centre, Rheinische Friedrich-Wilhelms-Universität Bonn, Bonn, Germany

^4^Centre for Integrated Oncology, Cologne-Bonn, Rheinische Friedrich-Wilhelms-Universität Bonn, Bonn, Germany

^5^European Medical Association, Brussels, Belgium

^6^Laboratory of Molecular Pathology, Department of Pathology, University of Malta, Malta

*Correspondence: Prof. Dr. Olga Golubnitschaja, Radiological Clinic, Rheinische Friedrich-Wilhelms-Universität Bonn, Sigmund-Freud-Str 25, 53105 Bonn, Germany; e.mail: Olga.Golubnitschaja@ukbonn.de

**Keywords**: Predictive Preventive Personalised Medicine, Cancer, Neurodegenerative Neurological Neuropsychiatric Disorders, Cardiovascular disease, Diabetes mellitus, Innovative technologies, Dentistry, Healthcare strategies, Health policy 

EPMA was created in 2009 as the Forum for Predictive, Preventive and Personalised Medicine (PPPM). During the 8 years since then, EPMA meetings – in the form of world congresses and international summits [1-3] – have traditionally included multi-professional presentations, discussions, considerations, publications, and consolidation in the field of PPPM. The EPMA Journal releases over 90% of publications dedicated to PPPM worldwide, acting as the leading journal in the field. EPMA currently comprises a network of over 50 countries globally, actively contributing to the innovative concepts of PPPM. For more information, please see www.epmanet.eu

The EPMA World Congress 2017 in Malta is specifically focused on the following topics: PPPM in Cancer, PPPM in Neurodegenerative Neurological Neuropsychiatric Disorders (NNND), PPPM in Cardiovascular Disease, PPPM in Diabetes Mellitus and related pathologies, PPPM in Dentistry, Innovative Technologies in PPPM, and Innovative PPPM Strategies in Healthcare.

Global challenges and PPPM-solutions: Long-term strategies of the EPMA (keynote speech by the EPMA Secretary-General, Prof. Dr. Olga Golubnitschaja, September 14^th^) will be presented based on the “EPMA position paper 2016” as the fundamental document of the association [4]. Several approaches have been suggested in modern medicine for optimising medical services, including person-centred, personalised, individualised, and precision medicine. The great plurality of approaches indicates a broad understanding of clear deficits which do exist in currently applied medical services and the attempts of diverse professional groups to remedy them. On the other hand, there is a growing understanding that persisting deficits are fundamental in nature and, therefore, cannot be solved by superficial modifications of health care systems facilitating individual technologies such as “cancer genomics” by “precision medicine.” Global deficits are well-defined and have been described elsewhere as unpredictable, unpreventable, and impersonal medicine. It is evident that a paradigm shift is needed to move from “reactive” to “predictive, preventive and personalised medicine” as a new philosophy for medical services covering both “health care” and “disease care”, promoting an integrated approach combining advantages of individual biomedical fields and technologies, and consolidating a multi-professional collaboration. An individual with an actual or potential disease wants a medicine in which he/she is at the centre, a medicine which is tailored to his/her polymorphism, a medicine which is able to provide him/her with the right therapy, in the right dose, at the right moment, for the right period of time. But he/she also wants a medicine which is able to predict and prevent possible diseases. He/she is not interested in the way in which this kind of medicine is described. However, he/she is interested in understanding why it is described in that way, in order to appreciate its potential to restore health. And this is the real advantage of speaking in terms of predictive, preventive, and personalised medicine: the actual or potential patient understands what is going on! Nevertheless, there is something more. PPPM lends itself to the role of an overarching umbrella under which the main ethical issues of contemporary biomedicine can be positively tackled.

Flammer syndrome (FS) in the context of PPPM (keynote speech by Prof. Dr. Josef Flammer and Dr. Katarzyna Konieczka, Ophthalmologic Clinic, University of Basel, September 15^th^) will present a special phenotype/health condition which is clearly relevant for disease development and individual outcomes in several pathologies including glaucoma, multiple sclerosis, breast cancer, and metastatic disease, amongst others [5-9]. FS is frequently observed in young populations (the symptoms appear early in puberty), making innovative PPPM approaches particularly meaningful in the area of population screening, predictive diagnostics, targeted prevention, and individualised prognosis.

Innovative healthcare strategies

PPPM represents only innovative biomedical sciences but also advanced healthcare services focusing on the patient’s needs and the most optimal solutions for society as the whole. The practical aspects of the paradigm change from “unPPPM” to PPPM will be discussed at dedicated sessions including global healthcare benefits (keynote speech by Prof. Dr. Russel Andrews, NASA, USA, September 16^th^), socioeconomic aspects of the PPPM implementation, and creation of new institutions to facilitate the development of PPPM services in healthcare.

Breast Cancer Epidemic in the early Twenty-First century [10] is the “leitmotiv” of the specialised congress session “PPPM in Cancer”. PPPM approaches will be discussed to apply multi-level diagnostics in order to improve the patient stratification, prediction of disease development and progression, targeted preventive measures, and personalised treatment algorithms [11].

PPPM in Neurological, Neurodegenerative and Neuropsychiatric Disorders (NNND) is a specialised session which will present the top expertise in the relevant areas including computer-assisted disease-modelling, personalised approaches to diagnosis and treatment in psychiatry, neuroinflammation, neurodegeneration, suboptimal health conditions, and comorbidities, amongst others.

PPPM in Diabetes Mellitus is a specialised session dedicated to the innovative approaches for predicting, preventing, and effectively treating the metabolic syndrome, its pre-stages and comorbidities, and special health conditions such as pregnancy.

PPPM in Cardiovascular Disease (CVD) is a specialised session featuring a collection of innovative data regarding CVD prevention in stratified cohorts, as well as molecular pathways targeted for CVD prediction and prognosis.

PPPM in Dentistry is a specialised session with multi-professional expertise which will present a whole spectrum of the field related innovation including the role of stem cells in tooth regeneration (keynote speech by Prof. Dr. Mahmood Mozaffari, University of Augusta, USA, September 16^th^), dental health in childhood, caries prevention in stratified patient cohorts, personalised orthopaedic treatments, and prevention of severe complications in CVD patients with dental pathologies, amongst others.

Innovative Technologies in PPPM is a specialised session with multi-professional expertise which will introduce unique technologies such as liquid biopsy (keynote speech by Prof. Dr. Evi S. Lianidou, University of Athens, Greece, September 15^th^), health condition-specific biomarker panels discovered in human tear fluid and saliva, and disease-specific multi-omics approaches, amongst others.

Workshop of young professionals in PPPM (EPMA-YPS, September 14 ^th^ ) is the traditional session presenting new ideas developed by young researchers, including “Mini-encyclopaedia of Wound Healing: Lessons for PPPM”, “Longevity – good luck or reasonable lifelong healthcare strategy?” The best presentations chosen by the international jury-board of prominent scientists will be awarded by the EPMA. The award ceremony will take place on September 16^th^.

The University of Malta, as the host of the EPMA World Congress 2017, has created an excellent platform for an effective congress and a pleasant atmosphere for the thematic sessions scheduled in the congress programme, as well as spontaneous professional meetings to exchange professional information and to plan new, successful projects in PPPM.

**Welcome to the EPMA World Congress 2017 in Malta – enjoy the meeting!** 
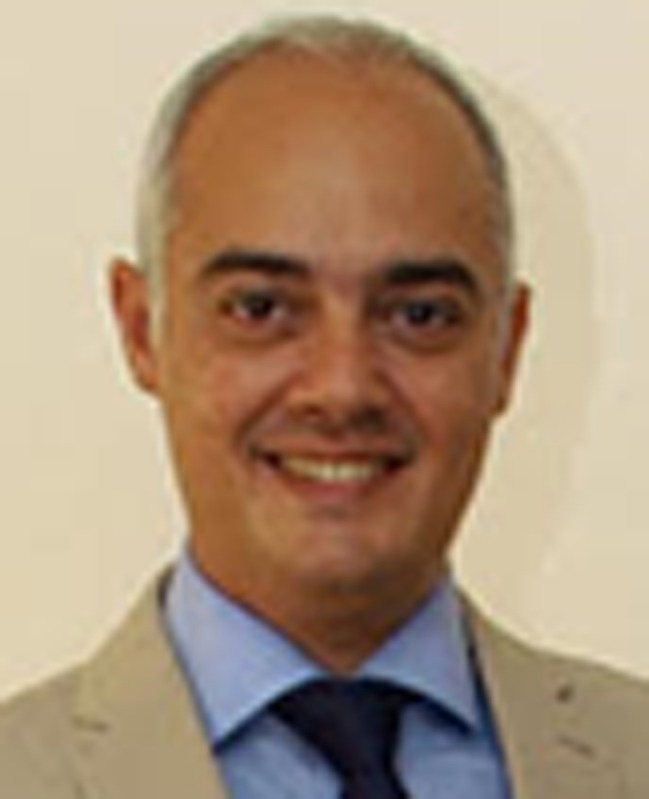


Prof. Dr. Godfrey Grech

National Representative of the EPMA in Malta
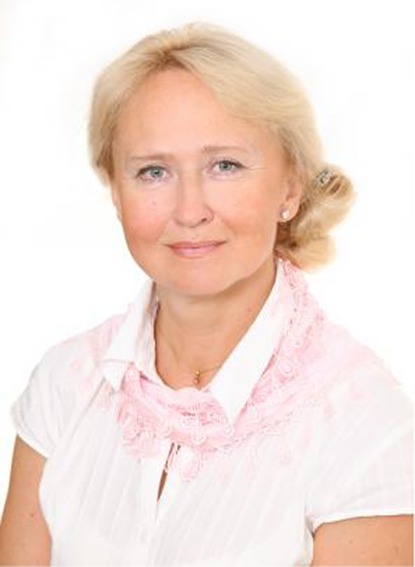


Prof. Dr. Olga Golubnitschaja

Secretary-General of the EPMA
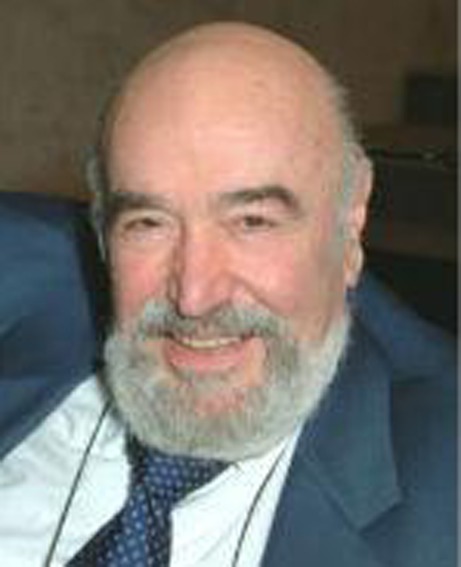


Dr. Vincenzo Costigliola

President of the EPMA

**References**

1. Golubnitschaja O, Costigliola V. European strategies in predictive, preventive and personalised medicine: highlights of the EPMA World Congress 2011. EPMA J 2011;2(4): 315–332. doi:10.1007/s13167-011-0134-6.
2. Golubnitschaja O, Costigliola V and EPMA General report & recommendations in predictive, preventive and personalised medicine 2012: white paper of the European association for predictive, preventive and personalised medicine. EPMA J 2012;1;3(1):14. doi:10.1186/1878-5085-3-14.
3. Golubnitschaja O, Costigliola V, EPMA. EPMA summit 2014 under the auspices of the presidency of Italy in the EU: professional statements. EPMA J 2015;6(1):4. doi:10.1186/s13167-015-0026-2.
4. Golubnitschaja O, Baban B, Boniolo G, Wang W, Bubnov R, Kapalla M, Krapfenbauer K, Mozaffari M, Costigliola V. Medicine in the early twenty-first century: paradigm and anticipation – EPMA position paper 2016. EPMA J 2016;7:23, doi:10.1186/s13167-016-0072-4.
5. Konieczka K, Ritch R, Traverso CE, Kim DM, Kook MS, Gallino A, Golubnitschaja O, Erb C, Reitsamer HA, Kida T, Kurysheva N, Yao K. Flammer syndrome. EPMA J 2014;5(1):11. doi:10.1186/1878-5085-5-11.
6. Konieczka K, Koch S, Binggeli T, Schoetzau A, Kesselring J. Multiple sclerosis and primary vascular dysregulation (Flammer syndrome). EPMA J. 2016 Jun 15;7:13. doi:10.1186/s13167-016-0062-6.
7. Zubor P, Gondova A, Polivka J Jr, Kasajova P, Konieczka K, Danko J, Golubnitschaja O. Breast cancer and Flammer syndrome: Any symptoms in common for prediction, prevention and personalised medical approach? EPMA J 2017. doi:10.1007/s13167-017-0089-3.
8. Bubnov R, Polivka J Jr, Zubor P, Koniczka K, Golubnitschaja O. “Pre-metastatic niches" in breast cancer: are they created by or prior to the tumour onset? "Flammer Syndrome" relevance to address the question. EPMA J 2017. doi:10.1007/s13167-017-0092-8.
9. Smokovski I, Risteski M, Polivka J Jr, Zubor P, Konieczka K, Costigliola V, Golubnitschaja O. Postmenopausal Breast Cancer: European Challenge and Innovative Concepts. EPMA J 2017. doi:10.1007/s13167-017-0094-6.
10. Golubnitschaja O, Debald M, Yeghiazaryan K, Kuhn W, Pešta M, Costigliola V, Grech G. Breast cancer epidemic in the early 21st century: Evaluation of risk factors, cumulative questionnaires and recommendations for preventive measures Tumor Biol 2016;37(10):12941-12957. doi:10.1007/s13277-016-5168-x.
11. Grech G, Zhan X, Yoo BC, Bubnov R, Hagan S, Danesi R, Vittadini G, Desiderio DM. EPMA position paper in cancer: current overview and future perspectives. EPMA J. 2015;6(1):9. doi:10.1186/s13167-015-0030-6.

**PPPM IN CANCER**

**Breast cancer epidemic in the early 21st century: risk factors for disease onset and progression**

Olga Golubnitschaja^1,2,3^

^1^Radiological Clinic, Rheinische Friedrich-Wilhelms-Universität Bonn, Sigmund-Freud-Str 25, 53105 Bonn, Germany

^2^Breast Cancer Research Centre, Rheinische Friedrich-Wilhelms-Universität Bonn, Bonn, Germany

^3^Centre for Integrated Oncology, Cologne-Bonn, Rheinische Friedrich-Wilhelms-Universität Bonn, Bonn, Germany

*Correspondence: Prof. Dr. Olga Golubnitschaja, Radiological Clinic, Rheinische Friedrich-Wilhelms-Universität Bonn, Sigmund-Freud-Str 25, 53105 Bonn, Germany; e.mail: Olga.Golubnitschaja@ukbonn.de

**Keywords**: Breast cancer, Metastatic disease, Predictive preventive personalised medicine, Systems medicine, Individualised patient profile, Multi-level diagnostics, Biomarker panel, Health policy

**Abstract**

The breast cancer (BC) epidemic in the twenty-first century is evidenced by the approximately two million new cases and half-million related deaths annually worldwide [1]. The overall situation is particularly dramatic in specific subgroups such as those with triple-negative BC: more than 50% of these patients die of metastatic BC within the first 6 months of diagnosis. Consequently, breast cancer management in general should be thoroughly reconsidered, promoting a new paradigm based on predictive diagnostics, new screening programs, targeted prevention and individually tailored treatments. Due to the extremely high level of complexity of the field, the new paradigm represents a spectrum of complementary components that constitute the innovative PPPM concept. At the EPMA World Congress 2015, the dedicated working group elaborated the working hypothesis and concepts for a multi-centre pilot project; this action has consequently been taken [2], and the new knowledge collected will be summarised. The overall results substantially extend the “seed and soil” theory of metastasis with the finding that among individuals at risk, a strong predisposition toward the formation of the systemic hypoxic pre-metastatic niches can be established long before the breast malignancy is clinically manifested [3]. The “Flammer syndrome” phenotype may strongly contribute to BC onset [4] and aggressive metastatic disease with particularly poor outcomes [4]. Both pre- and postmenopausal women may be affected [3-5]. Individualised patient profiles and the involvement of primary caregivers (family doctors) are essential for implementation of PPPM in BC management [5].

**References**

1. Golubnitschaja O, Debald M, Yeghiazaryan K, Kuhn W, Pešta M, Costigliola V, Grech G. Breast cancer epidemic in the early 21st century: Evaluation of risk factors, cumulative questionnaires and recommendations for preventive measures Tumor Biol 2016;37(10):12941-12957. doi:10.1007/s13277-016-5168-x.
2. Golubnitschaja O, Debald M, Kuhn W, Yeghiazaryan K, Bubnov RV, Goncharenko VM, Lushchyk U, Grech G, Konieczka K. Flammer Syndrome and potential formation of pre-metastatic niches: A multi-centred study on phenotyping, patient stratification, prediction and potential prevention of aggressive breast cancer and metastatic disease. EPMA J 2016;7(Suppl 1):9,A25. doi:10.1186/s13167-016-0054-6.
3. Bubnov R, Polivka J Jr, Zubor P, Koniczka K, Golubnitschaja O. Pre-metastatic niches" in breast cancer: are they created by or prior to the tumour onset? "Flammer Syndrome" relevance to address the question. EPMA J 2017. doi:10.1007/s13167-017-0092-8
4. Zubor P, Gondova A, Polivka J Jr, Kasajova P, Konieczka K, Danko J, Golubnitschaja O. Breast cancer and Flammer syndrome: Any symptoms in common for prediction, prevention and personalised medical approach? EPMA J 2017. doi:10.1007/s13167-017-0089-3.
5. Smokovski I, Risteski M, Polivka J Jr, Zubor P, Konieczka K, Costigliola V, Golubnitschaja O. Postmenopausal Breast Cancer: European Challenge and Innovative Concepts. EPMA J 2017. doi:10.1007/s13167-017-0094-6.

**Application of molecular medicine towards personalised treatment in oncology**

Godfrey Grech ^1^

^1^ Laboratory of Molecular Pathology, Department of Pathology, University of Malta, Malta

*Correspondence: Prof. Godfrey Grech, Laboratory of Molecular Pathology, Department of Pathology, University of Malta, Malta; e.mail: godfrey.grech@um.edu.mt

**Keywords**: Molecular medicine, Predictive biomarkers, Breast cancer

**Abstract**

Molecular medicine has evolved rapidly over the past decade, focusing on genetics and cellular mechanisms of disease, providing knowledge on (1) causative genes in monogenic diseases and syndromes, as well as susceptibility genes that work in concert with other genetic and environmental factors to elicit the disease; and (2) deregulated mechanisms within specific subtypes of disease, to classify patients molecularly and to identify potential or already known therapeutic targets. In oncology, research and its evidence-based scientific outcomes have evolved from the lab bench to bedside, implementing the use of biomarkers as classifiers of therapeutic groups; characterization of syndromes with high risk for specific disease, hence providing the opportunity for risk reduction strategies; predictive biomarkers to guide treatment type, dose and toxicity; and prognostic biomarkers to define specific clinical outcomes. Preventive genetics plays a role in defining specific genetic disorders through population screening for carriers of rare, fully penetrant alleles that cause monogenic diseases, and genotyping of susceptibility genes within families at high risk of developing a specific disease, providing the basis of public health genetics. Predictive genetics deals with the efficacy and toxicity of drugs in individuals. Breast cancer will be discussed as a model for the use of molecular markers; the use of technology to bridge the gap between laboratory and clinical setting; and the need for novel targets to overcome targeted therapy resistance. The uptake of molecular medicine in the healthcare system requires continuous education of healthcare professionals and proper dissemination of information within the healthcare system.

**Dietary factors in breast cancer prevention**

Niva Shapira, Ph.D., R.D., Agr.*^1^

^1^Department of Nutritional Science and Diet, School of Health Professions

*Correspondence: Niva Shapira, Department of Nutritional Science and Diet, School of Health Professions, Ashkelon Academic College, 12 Ben Zvi St., P.O.B 1071, Ashkelon, Israel

E-mail: nivnet@inter.net.il

**Keywords:** Breast cancer, Nutritional prevention gender nutrition, Estrogen, Obesity, Metabolic syndrome, Plant-based diet, Antioxidants, DNA adducts

**Abstract**

Breast cancer (BC), the leading cancer in women, is increasing in prevalence worldwide, concurrent with western metabolic epidemics including obesity, metabolic syndrome, and diabetes, and shares major risk factors with these diseases. Here, potential dietary contributions to BC prevention are reviewed, with a focus on their pathometabolic trajectories.

The development of BC potentially involves diet-related pro-oxidative, inflammatory, and pro-carcinogenic processes, i.e. through lipid/fatty acid peroxidation, estrogen metabolism, related DNA adduct depurination, and mutation formation [1]; free fatty acids (FFA), e.g. the n-6 polyunsaturated fatty acid (PUFA) linoleic acid, have been associated with BC migration and invasion [2].

The pathometabolic trajectory is affected by high estrogen, insulin, and growth factor cascades and the resultant accelerated proliferation/progression [3]. Anthropometric measures – high birth weight, adult tallness, fatness/body mass index (BMI), and weight-gain – are often reflective of these factors [3, 4].

A gender-based approach targets women’s specific nutritional risk in western obesogenic environments, i.e. with increasing adiposity, estrogen metabolism, and n-6 PUFA conversion to pro-inflammatory/carcinogenic eicosanoids under high n-6:n-3 PUFA ratio conditions [5]; and as related to the timing of life events, such as lower age at menarche, late full-term pregnancy, and early menopause [6].

Recent large-scale studies have confirmed the effectiveness of evidence-based recommendations against BC risk, emphasizing low dietary energy density (ED), nutritious plant-based diets, physical activity, and body/abdominal adiposity management [7, 8].

A better understanding of dietary interrelationships with BC and recommended dietary patterns – e.g. Mediterranean, DASH, plant-based, low-ED, and glycemic load (GL), with high nutrient/phytonutrient density – will be discussed, with emphasis on the need for personalized adaptation to support early/timely prevention, optimally merging with other dietary/health goals, for lifelong BC prevention.

**References**

1. Cavalieri EL, Rogan EG. The etiology and prevention of breast cancer. Drug Discov Today Dis Mech. 2012 Summer;9:e55-e69.
2. Byon CH, Hardy RW, Ren C, Ponnazhagan S, Welch DR, McDonald JM, Chen Y. Free fatty acids enhance breast cancer cell migration through plasminogen activator inhibitor-1 and SMAD4. Lab Invest. 2009 Nov;89:1221-8.
3. Endogenous H, Breast Cancer Collaborative G, Key TJ, Appleby PN, Reeves GK, Roddam AW, Helzlsouer KJ, Alberg AJ, Rollison DE, et al. Circulating sex hormones and breast cancer risk factors in postmenopausal women: reanalysis of 13 studies. Br J Cancer. 2011 Aug 23;105:709-22.
4. Berrino F, Villarini A, Traina A, Bonanni B, Panico S, Mano MP, Mercandino A, Galasso R, Barbero M, et al. Metabolic syndrome and breast cancer prognosis. Breast Cancer Res Treat. 2014 Aug;147:159-65.
5. Shapira N. Women's higher risk with N-6 PUFA vs. men's relative advantage: an "N-6 gender nutrition paradox" hypothesis. Isr Med Assoc J. 2012 Jul;14:435-41.
6. Shapira N. Women's higher health risks in the obesogenic environment: a gender nutrition approach to metabolic dimorphism with predictive, preventive, and personalised medicine. The EPMA journal. 2013;4:1.
7. Catsburg C, Miller AB, Rohan TE. Adherence to cancer prevention guidelines and risk of breast cancer. Int J Cancer. 2014 Nov 15;135:2444-52.
8. Hastert TA, Beresford SA, Patterson RE, Kristal AR, White E. Adherence to WCRF/AICR cancer prevention recommendations and risk of postmenopausal breast cancer. Cancer Epidemiol Biomarkers Prev. 2013 Sep;22:1498-508.

**Heterogeneity in breast cancer**

Elaine Borg^1,2^, Pierre Schembri Wismayer^**2**^ and Godfrey Grech^**2**^

^1^ Mater Dei Hospital, Malta

^2^ University of Malta, Malta

*Correspondence: Ms Elaine Borg, Surgery Department, Mater Dei Hospital, Malta. Email: elaineborg86@gmail.com

**Keywords:** Breast cancer, Heterogeneity, Immunohistochemistry, PI3k/pAKT pathway

**Abstract**

Breast cancer is by far the most common cancer in women worldwide. The World Health Organization (WHO) claims that there are about 1.38 million new cases and 458,000 deaths from breast cancer each year [1]. The Malta Cancer registry showed that 13% of cancer deaths in Malta are due to breast cancer, the second highest rate in the EU [2].

Exploring the molecular difference among breast cancer subtypes is of crucial importance in understanding its heterogeneity and seeking its effective clinical treatment. Predicting drug response in cancer patients remains a major challenge in the clinic; one of the reasons is heterogeneity [3]. Ex vivo culturing enables a personalized culture method, which allows for investigation of anti-tumoral pharmacological properties that preserves the original cancer microenvironment [4]. It is believed that molecular predictive markers may form the basis for tailoring an individual’s adjuvant therapy based on the genetic fingerprint of their cancer.

This study aims to assess heterogeneity in breast cancer tissue resected in Malta using both routine immunohistochemistry and that specific to the PI3K/pAkt pathway. Using LIS, PATHSA and iSOFT software, a curated dataset of patients who had documented tumoral heterogeneity between 2008 and 2015 was created. Demographics that were noted included gender, age, histology types, Bloom-Richardson grade, type of surgery the patient underwent, presence of axillary micrometastasis or macrometastasis, and vascular invasion. The tissue blocks with confirmed heterogeneity were traced using LIS software and retrieved from the storage unit. Sections from these tissue blocks were used to run tests to assess tissue architecture with H&E and immunohistochemistry. Different techniques for ex vivo culturing of breast tissue are explored. Results will be analysed using SPSS.

**References**

1. Global Health Estimates, WHO 2013
2. Malta National Cancer Registry. Cancer in the Maltese islands.. 2015.
3. Rivenbark AG, O'Connor SM, Coleman WB. Molecular and cellular heterogeneity in breast cancer: Challenges for personalized medicine. Am J Pathol. 2013;183(4):1113-1124.
4. Vaira V, Fedele G, Pyne S, Fasoli E, Zadra G, Bailey D, Snyder E, Faversani A, Coggi G, Flavin R, Bosari S, Loda M. Preclinical model of organotypic culture for pharmacodynamic profiling of human tumours. Proc Natl Acad Sci USA 2010

**Predictive modelling of breast cancer in patients with breast benignancy**

Holger Fröhlich^1^, Sabyasachi Patjoshi^1^, Walther Kuhn^2,3,4^,

Olga Golubnitschaja*^3,4,5^

^1^Bonn-Aachen International Center for IT, Friedrich-Wilhelms-Universität Bonn, Germany

^2^Centre for Obstetrics and Gynaecology, Rheinische Friedrich-Wilhelms-Universität Bonn, Germany

^3^Breast Cancer Research Centre, Rheinische Friedrich-Wilhelms-Universität Bonn, Germany

^4^Centre for Integrated Oncology, Cologne-Bonn, Rheinische Friedrich-Wilhelms-Universität Bonn, Germany

^5^Radiological Clinic, Rheinische Friedrich-Wilhelms-Universität Bonn, Germany

*Correspondence: Prof. Dr. Olga Golubnitschaja, Radiological clinic, Rheinische Friedrich-Wilhelms-Universität Bonn, Sigmund-Freud-Str. 25, 53105 Bonn, Germany; e.mail: Olga.Golubnitschaja@ukbonn.de

**Keywords**: Predictive preventive personalised medicine, Breast cancer, Bioinformatics, Disease modelling, Multi-level diagnostics, Biomarker panel, Laboratory medicine

**Abstract**

Research background

The breast cancer (BC) epidemic is a hallmark of the early twenty-first century [1]. BC is a multifactorial disease: BC experts utilise the plural form, now referring to “breast cancers” [2]. Contextually, the BC patient cohort demonstrates the whole spectrum of subgroups, including both antipodes: on one side this is the “postmenopausal breast cancer as the European challenge” [3], and on the other the particularly aggressive premenopausal “metastatic breast cancer” [4]. The latter subgroup is particularly unpredictable, comprising sporadic BC cases with highly promoted metastatic spread to the vital organs [5]. Although postmenopausal BC cases still represent the majority of the overall patient cohort, recent statistics demonstrate that young populations are increasingly affected by BC [1]. Therefore, the old rule "the older the age, the higher the BC risk" is becoming relativized. Contextually, BC prediction and consequent prevention is a highly complex task requiring effective computation of individual patient profiles and precise modelling of the disease [1].

Results and conclusions

Our pilot project dedicated to the predictive modelling of BC has utilised data collected using a multi-level diagnostic approach in BC patients versus breast benignancy. The study revealed two clearly separate clusters with high versus low BC similarity in premenopausal BC-free individuals. The developed model is capable of distinguishing “high” and “low” BC risk with high confidence (>90%). This diagnostic approach is based on a disease-specific molecular signature including signalling and detoxification pathways, longevity-related proteins, regulation of cytoskeleton, and tissue remodelling. Large-scale validation of the clinical utility of the model is under consideration.

**References**

1. Golubnitschaja O, Debald M, Yeghiazaryan K, Kuhn W, Pešta M, Costigliola V, Grech G. Breast cancer epidemic in the early 21st century: Evaluation of risk factors, cumulative questionnaires and recommendations for preventive measures Tumor Biol 2016;37(10):12941-12957. doi:10.1007/s13277-016-5168-x.
2. Golubnitschaja O. Feeling cold and other underestimated symptoms in breast cancer: anecdotes or individual profiles for advanced patient stratification? EPMA J 2017;8(1),17-22. doi:10.1007/s13167-017-0086-6.
3. Smokovski I, Risteski M, Polivka J Jr, Zubor P, Konieczka K, Costigliola V, Golubnitschaja O. Postmenopausal Breast Cancer: European Challenge and Innovative Concepts. EPMA J 2017. doi:10.1007/s13167-017-0094-6.
4. Bubnov R, Polivka J Jr, Zubor P, Koniczka K, Golubnitschaja O. Pre-metastatic niches" in breast cancer: are they created by or prior to the tumour onset? "Flammer Syndrome" relevance to address the question. EPMA J 2017. doi:10.1007/s13167-017-0092-8.
5. Polivka J Jr, Kralickova M, Polivka J Jr, Kaiser C, Kuhn W, Golubnitschaja O. Mystery of the brain metastatic disease in breast cancer patients: improved patient stratification, disease prediction and targeted prevention on the horizon? EPMA J 2017. doi:10.1007/s13167-017-0087-5.

**Predictive molecular signature in blood of premenopausal triple-negative breast cancer patients**

Nora Filep^1^, Kristina Yeghiazaryan^2,3,4^, Martin Hofmann-Apitius^1^,

Walther Kuhn^3,4,5^, Olga Golubnitschaja*^2,3,4^

^1^Department of Bioinformatics, Fraunhofer Institute for Algorithms and Scientific Computing (SCAI), Sankt Augustin, Germany

^2^Department of Radiology, Rheinische Friedrich-Wilhelms-Universität Bonn, Sigmund-Freud-Str 25, 53105 Bonn, Germany

^3^Breast Cancer Research Centre, Rheinische Friedrich-Wilhelms-Universität Bonn, Bonn, Germany

^4^Centre for Integrated Oncology, Cologne-Bonn, Rheinische Friedrich-Wilhelms-Universität Bonn, Bonn, Germany

^5^Centre for Obstetrics and Gynaecology, Rheinische Friedrich-Wilhelms-Universität Bonn, Bonn, Germany

*Correspondence: Prof. Dr. Olga Golubnitschaja, Department of Radiology, Rheinische Friedrich-Wilhelms-Universität Bonn, Sigmund-Freud-Str. 25, 53105 Bonn, Germany; e.mail: Olga.Golubnitschaja@ukbonn.de

**Keywords**: Predictive preventive personalised medicine, Multi-level diagnostics, Biomarker panel, Laboratory medicine, Blood test, Oncology, Healthcare economy

**Abstract**

The breast cancer (BC) epidemic is currently recognised as a feature of the early twenty-first century, with almost two million new cases and a half-million BC-related deaths recorded annually [1]. Over 90% of all BC cases are sporadic in nature, which means currently unpredictable. The main cause of BC-related deaths is aggressive metastatic disease to distant organs – bones, lung, liver and brain, amongst others.

The premenopausal triple-negative breast cancer (pTNBC) subtype is characterised by particularly low predictability and poor prognosis: early diagnostic methods are underdeveloped and therapy approaches are rather un-targeted. Consequently, more than 50% of these patients die within the first 6 months of the diagnosis of metastatic disease [2], and up to 30% experience brain metastasis, with particularly poor outcomes [3]. Individualised profiling for advanced patient stratification has been recommended through the introduction of innovative patient management by prediction, prevention and personalised treatment algorithms for this highly specific patient cohort [4].

The predictive molecular signature in the blood of pTNBC patients has been identified using differential molecular profiles by protein patterns of circulating leukocytes, specific serum metabolites and subcellular imaging to evaluate the quality of chrDNA. Statistically significant differences have been demonstrated for the molecular signature of pTNBC patients compared to those with premenopausal benignancy, premenopausal E/P-receptor-positive BC and all postmenopausal BC. Additional stratification by the activity level of MMP2 and MMP9 in blood serum further improves the overall predictive power of our diagnostic approach [5]. As for the next stage, a large-scale validation in a multi-centre study is currently planned.

**References**

1. Golubnitschaja O, Debald M, Yeghiazaryan K, Kuhn W, Pešta M, Costigliola V, Grech G. Breast cancer epidemic in the early 21st century: Evaluation of risk factors, cumulative questionnaires and recommendations for preventive measures Tumor Biol 2016;37(10):12941-12957. doi:10.1007/s13277-016-5168-x.
2. Vaz-Luis I, Lin NU, Keating NL, Barry WT, Winer EP, Freedman RA. Factors Associated with Early Mortality Among Patients with De Novo Metastatic Breast Cancer: A Population-Based Study. Oncologist. 2017; doi:10.1634/theoncologist.2016-0369.
3. Polivka J Jr, Kralickova M, Polivka J Jr, Kaiser C, Kuhn W, Golubnitschaja O. Mystery of the brain metastatic disease in breast cancer patients: improved patient stratification, disease prediction and targeted prevention on the horizon? EPMA J 2017. doi:10.1007/s13167-017-0087-5.
4. Golubnitschaja O. Feeling cold and other underestimated symptoms in breast cancer: anecdotes or individual profiles for advanced patient stratification? EPMA J 2017;8(1),17-22. doi:10.1007/s13167-017-0086-6.
5. Golubnitschaja O, Yeghiazaryan K, Abraham JA, Schild HH, Costigliola V, Debald D, Kuhn W. Breast Cancer Risk Assessment: A Non-invasive Multiparametric Approach to Stratify Patients by MMP-9 Serum Activity and RhoA Expression Patterns in Circulating Leucocytes. Amino Acids 2017;49(2), 273-281. doi:10.1007/s00726-016-2357-2.

**Novel biomarkers predicting sensitivity to PP2A activators in triple-negative breast cancer**

Christian Saliba ^1^, Shawn Baldacchino^2^, Christian Scerri^3^, Robert Gauci^2^, Godfrey Grech^2^

^1^ Centre for Molecular Medicine and Biobanking, University of Malta

^2^ Laboratory of Molecular Pathology, Department of Pathology, University of Malta, Malta

^3^ Department of Physiology & Biochemistry, Faculty of Medicine and Surgery, University of Malta

*Correspondence: Prof. Godfrey Grech, Laboratory of Molecular Pathology, Department of Pathology, University of Malta, Malta; e.mail: godfrey.grech@um.edu.mt

**Keywords**: PP2A, Laser microdissection, FFPE, Predictive biomarkers, Triple-negative breast cancer

**Objective**: Triple-negative breast cancer (TNBC) patients derive little benefit from target-specific therapies due to a lack of favourable prognostic targets, ER, PR and HER2. The aim of this project was to define biomarkers to measure PP2A complex activity in breast cancer predicting a novel therapeutic class.

**Methods**: A multiplex assay using branched DNA technology was used to measure the expression of 40 genes in RNA derived from cell lines (*n*=12) and formalin-fixed, paraffin-embedded (FFPE) archival material from breast cancer patients (2009–2011). First, the PP2A activity biomarkers (PABs) were validated in breast cancer cellular models and compared to real-time PCR results. The QuantiGene 2.0 multiplex assay was used to measure expression of these markers in a cohort of laser-microdissected breast cancer (*n*=97) and normal breast tissue cases (*n*=30). Sensitivity of cell lines to FTY720 was measured using MTT assays.

**Results**: Overexpression of these biomarkers in cell lines predicted sensitivity to FTY720 and were down-regulated in a dose-dependent manner. Of interest, 37% of TNBC cases expressed high levels of PABs, and were associated with negative prognostic indicators (*P* = 0.023).

**Conclusion**: TNBC cell lines sensitive to the PP2A activator FTY720 showed a high expression of PABs. Using patient material, PAB overexpression was common in the TNBC subtype. The novel biomarkers (PABs) provide a multiplex gene expression signature for a potential therapeutic group sensitive to PP2A activators.

**Accelerated ageing in breast cancer aetiology and progression: Multi-factorial patient profiles for effective prediction and prevention of the disease**

Aviv Peer^1^ and Olga Golubnitschaja^2,3,4^

^1^The Ruth and Bruce Rappaport Faculty of Medicine, Technion-Israel Institute of Technology, Haifa, 31096, Israel

^2^Radiological Clinic, Rheinische Friedrich-Wilhelms-Universität Bonn, Sigmund-Freud-Str 25, 53105 Bonn, Germany

^3^Breast Cancer Research Centre, Rheinische Friedrich-Wilhelms-Universität Bonn, Bonn, Germany

^4^Centre for Integrated Oncology, Cologne-Bonn, Rheinische Friedrich-Wilhelms-Universität Bonn, Bonn, Germany

*Correspondence: Prof. Dr. Olga Golubnitschaja, Radiological Clinic, Rheinische Friedrich-Wilhelms-Universität Bonn, Sigmund-Freud-Str 25, 53105 Bonn, Germany; e.mail: Olga.Golubnitschaja@ukbonn.de

**Keywords**: Predictive preventive personalised medicine, Systems medicine, Individualised patient profile, Multi-level diagnostics, Biomarker panel, Ageing, Breast cancer, Environment, Risk factors

**Abstract**

Ageing has been associated with a great number of chronic and severe pathologies including cardiovascular disease, neurodegenerative disorders and cancer [1]. According to recent scientific reports, breast tissue is far older than other tissues in the body [2]. Consequently, accelerated ageing might be a specific risk factor for breast cancer development. Here we summarise currently available facts to support this hypothesis.

Lifestyle factors involved in both accelerated ageing and breast cancer risk:

Educational level, sociodemographic and marital status; tobacco and alcohol consumption; sedentary lifestyle against physical activity; participation in social networks and leisure activities; workload and occupational environment; nutrition and diet composition; sleep patterns [3].

Pathologies associated with both ageing and (breast) cancer predisposition:

Overweight/obesity; chronic inflammation; diabetes mellitus; autoimmune diseases; endocrine dysfunction; immune dysfunction; cognitive dysfunction; dental disorders; impaired wound healing [1,4,5].

Specific patient profiles for predictive diagnostics and targeted preventive measures:

Genetic predisposition (inborn syndromes, symptoms and disorders); specific molecular profiles (biomarker panels including “intrinsic epigenetic age acceleration” profiles, CpG-methylation patterns, detoxification pathways, DNA-repair patterns, energy balance, amongst others) in blood, saliva and urine; symptoms of the pathologies associated with ageing (such as impaired wound healing); lifestyle factors listed above.

The presented profiles are the subject of follow-up studies to be validated for their practical application within innovative population screening and educational programmes.

**References**

1. Ferrucci L, Giallauria F, Guralnik JM. Epidemiology of Aging. Radiol Clin North Am. 2008;46(4):643–v.
2. Sehl ME, Henry JE, Storniolo AM, Ganz PA, Horvath S. DNA methylation age is elevated in breast tissue of healthy women. Breast Cancer Res Treat. 2017 doi:10.1007/s10549-017-4218-4.
3. Golubnitschaja O, Debald M, Yeghiazaryan K, Kuhn W, Pešta M, Costigliola V, et al. Breast cancer epidemic in the early twenty-first century: evaluation of risk factors, cumulative questionnaires and recommendations for preventive measures. Tumor Biology. 2016; 37(10): 12941–57. doi:10.1007/s13277-016-5168-x.
4. Fu MR, Axelrod D, Guth AA, Cleland CM, Ryan CE, Weaver KR, et al. Comorbidities and Quality of Life among Breast Cancer Survivors: A Prospective Study. Armer JM, editor. J Pers Med. 2015;5(3):229–42.
5. Avishai E, Yeghiazaryan K, Golubnitschaja O. Impaired wound healing: facts and hypotheses for multi-professional considerations in predictive, preventive and personalised medicine. EPMA J. 2017;8(1), 23-33. doi:10.1007/s13167-017-0081-y.

**Validation of a diagnostic breast cancer expression assay using retrospective FFPE tissue**

Shawn Baldacchino^1^, Sharon Falzon^2^, James DeGaetano^2^, Christian Saliba^3^, Jeanesse Scerri^4^, Christian Scerri^4^, Godfrey Grech*^,1^

^1^ Department of Pathology, Faculty of Medicine & Surgery, University of Malta

^2^ Cellular Pathology, Pathology Department, Mater Dei Hospital

^3^ Centre for Molecular Medicine and Biobanking, University of Malta

^4^ Department of Physiology & Biochemistry, Faculty of Medicine and Surgery, University of Malta

*Correspondence: Prof. Godfrey Grech, Biomedical Sciences Building, Level 3, Rm316, University of Malta, Msida, MSD2080, Malta; E-mail: godfrey.grech@um.edu.mt

**Keywords:** Biomarkers, Innovative technologies, Translational medicine, Breast cancer, Expression, FFPE, Diagnosis, Classification, Cancer subtypes, HER2, ER, Triple-negative, PgR

**Abstract**

Diagnosis of breast cancer subtypes determines therapeutic options and prognosis. These subtypes are hallmarked by the expression of the human epidermal growth factor receptor 2, and oestrogen and progesterone receptors (HER2, ER and PgR). An audit of breast cancer diagnosed in Malta between 2009 and 2016 indicates that 85% were ER-positive, 11% were HER2-positive, and 10% were negative for all receptors. Current guidelines for diagnosis of HER2 are based on immunohistochemistry and confirmatory testing by fluorescent in situ hybridisation (FISH). HER2 copy number assessment on 960 breast cancer samples (cBIOPortal [1, 2]) is estimated to detect 1.3% amplification in the absence of expression (false positives) and 4.2% diploid accompanied by overexpression of the gene (false negatives).

A multiplex branched DNA assay has been validated for quantifying gene expression on degraded RNA derived from formalin-fixed paraffin-embedded (FFPE) breast cancer tissue [3]. Using the diagnostic results as benchmark, this assay has average sensitivity and specificity of 100% and 97.8% for the determination of HER2 status and ER status, respectively. The gene expression assay is a novel, quick multiplex method that can accurately diagnose breast cancer subtypes, eliminating subjectivity of interpretation and minimising technical variation. This method has a wide range of possible applications in the diagnosis of tumours and is adapted to the current diagnostic workflow.

**References**

1. Cerami E, Gao J, Dogrusoz U, Gross BE, Sumer SO, Aksoy BA et al. The cBio Cancer Genomics Portal: An Open Platform for Exploring Multidimensional Cancer Genomics Data. Cancer Discovery. 2012;2(5):401-4. doi:10.1158/2159-8290.cd-12-0095.
2. Gao J, Aksoy BA, Dogrusoz U, Dresdner G, Gross B, Sumer SO et al. Integrative Analysis of Complex Cancer Genomics and Clinical Profiles Using the cBioPortal. vol 269. 2013.
3. Grech G, Baldacchino S. Molecular Classification of Breast Cancer Patients Using Formalin-fixed Paraffin-embedded Derived RNA Samples. Journal of Molecular Biomarkers & Diagnosis. 2016;01(s8). doi:10.4172/2155-9929.s8-016.

**A clinical audit on HER2-positive breast cancer treatment and response trends in Malta**

Jeanesse Scerri^1^*, Godfrey Grech^2^, Christian Scerri^1^, Donika Metaraku^3^

^1^ Physiology & Biochemistry Department, Faculty of Medicine & Surgery, University of Malta, Msida, Malta

^2^ Pathology Department, Faculty of Medicine & Surgery, University of Malta, Msida, Malta

^3^ Oncology Day Ward, Sir Anthony Mamo Oncology Centre, Msida Malta

*Correspondence: Jeanesse Scerri, Laboratory of Molecular Genetics, Biomedical Sciences Building, University of Malta. Tel: +356 2340 2774; E-mail: jsce4@um.edu.mt.

**Keywords**: Breast cancer, HER2, Trastuzumab, Herceptin®, RECIST, Personalized medicine

**Abstract**

Human epidermal growth factor receptor 2 (HER2)-positive breast cancer cases constitute about 11% of the local breast cancer burden. These patients are candidates for targeted treatment with the anti-HER2 monoclonal antibody trastuzumab (Herceptin®, Genentech), in conjunction with chemotherapy and hormonal therapy if required, as an adjuvant or neoadjuvant to surgery in operable cases. The rates of clinical response to treatment have not yet been recorded locally. A retrospective clinical audit is thus currently in progress in collaboration with the Sir Anthony Mamo Oncology Centre (SAMOC). All HER2-positive breast cancer patients tested between 2010 and 2016, extracted from Mater Dei Hospital laboratory records, were included for the review of patient clinical records from their treatment history files. The following information is being collected where available:

- Date of initial diagnosis
- Age at diagnosis
- Tumour stage and pathological grade
- Treatment regime (adjuvant, neoadjuvant or treatment for advanced disease)
- Tumour measurements before and during/after treatment
- Date of recorded tumour progression/metastasis/new lesion diagnosis
- Date of death in the case of deceased patients

All clinical and radiological responses are given a final assessment using the Response Evaluation Criteria In Solid Tumors (RECIST) 1.1. The findings to date will be presented and compared to published findings in other countries. This audit will allow treatment response rates to be correlated to laboratory findings on archival tissue specimens in the ongoing Breast Cancer Project at the University of Malta.

**Early variations of KRAS mutations in circulating cell-free tumor DNA: a promising biomarker for monitoring chemotherapy resistance in pancreatic cancer**

Rofi E^1^, Vivaldi C^2^, Del Re M^1^, Danesi R^1^

^1^Clinical Pharmacology and Pharmacogenetic Unit, Dept. of Clinical and Experimental Medicine, University of Pisa

^2^Medical Oncology Unit, Dept. of Translational Research and New Technologies in Medicine and Surgery, University of Pisa

*Correspondence: Dr. Marzia Del Re, Clinical Pharmacology and Pharmacogenetic Unit, Dept. of Clinical and Experimental Medicine, University of Pisa, Via Roma 55, 56126 Pisa, Italy; email: marzia.delre@gmail.com

**Keywords:** Pancreatic adenocarcinoma, KRAS, Biomarkers, cftDNA, ddPCR

**Abstract**

**Background.** CA 19-9 is the only approved biomarker for monitoring tumor response in patients with pancreatic adenocarcinoma (PDAC), but it has several limitations, highlighting the need for new predictive biomarkers [1]. Mutant KRAS is a driving oncogene, occurring in 75–95% of PDAC [2].

**Aim.** To develop an analysis tool based on plasma to monitor the variation in mutant KRAS alleles in PDAC being treated with first-line chemotherapy.

**Methods.** Twenty-seven PDAC patients were enrolled in this study. Six milliliters of plasma were prospectively collected at baseline, after 15 days of chemotherapy and at each clinical follow-up. CftDNA was extracted from plasma with a specific kit and analyzed by the Droplet Digital™ PCR (ddPCR) for KRAS mutations in codon 12 (G12X) and G13D.

**Results.** At baseline, 19 out of 27 patients (70.3%) were carriers of one of the KRAS mutations. Eight patients had a negative cftDNA mutKRAS at baseline, and one out of eight became positive at day 15, and another one at the first radiological evaluation. There was a statistically significant difference in progression-free survival (PFS) between patients with increased vs. stable/reduced cftDNA at the 15-day sample (median PFS 2.5 vs. 7.5 months, *p*=0.03; see Fig. 1). A statistically significant difference was also observed in median overall survival (OS; 6.5 vs. 11.5 months in cftDNA increase vs. reduction, respectively, *p*=0.009).

**Conclusions.** cftDNA analysis of mutKRAS could be a reliable non-invasive approach for early prediction of tumor progression and responsiveness to chemotherapy. However, in order to assess these preliminary data, further analyses are needed.

**References**

1. Ballehaninna UK, Chamberlain RS. The clinical utility of serum CA 19-9 in the diagnosis, prognosis and management of pancreatic adenocarcinoma: An evidence based appraisal. J Gastrointest Oncol. 2012 Jun;3(2):105-19. doi:10.3978/j.issn.2078-6891.2011.021.
2. Bailey P, Chang DK, Nones K, Johns AL, Patch AM, Gingras MC, et al. Genomic analyses identify molecular subtypes of pancreatic cancer. Nature. 2016 Mar 3;531(7592):47-52. doi:10.1038/nature16965.

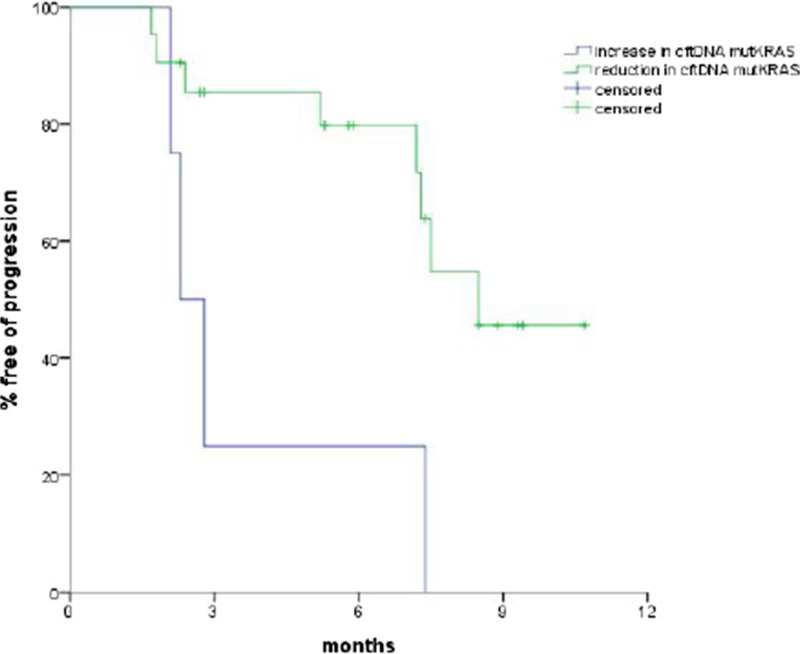


**Fig. 1** PFS according to cftDNA mutKRAS variation at day 15

**Role of microRNAs in diagnosis of lung cancers**

Erika Halasova^1,4^_,_ Martina Krutakova ^2,1^, Miroslava Sarlinova^1^, Tatiana Matakova^2,1^, Lukas Plank^3^

^1^Comenius University in Bratislava, Jessenius Faculty of Medicine, Biomedical Center Martin, Department of Molecular Medicine, Mala Hora 11161/4C Martin

^2^Comenius University in Bratislava, Jessenius Faculty of Medicine, Department of Medical Biology, Mala Hora 4, 03601 Martin, Slovak Republic

^3^Comenius University in Bratislava, Jessenius Faculty of Medicine, Department of Pathological Anatomy, Kollarova 2, 03601 Martin

^4^Comenius University in Bratislava, Jessenius Faculty of Medicine, Department of Medical Biology, Mala Hora 4, 03601 Martin, Slovak Republic

*Correspondence: Dr. Erika Halasova, E-mail: halasova@jfmed.uniba.sk

**Abstracts**

MicroRNAs represent an emerging class of non-coding RNAs with significant functions in the post-transcriptional regulation of gene expression. As they also regulate oncogenes and tumour-suppressive genes, microRNAs play an important role in the process of malignant transformation.

The aim of this study was to evaluate expression levels of selected candidate microRNAs (miR-221, miR-143, miR-145, miR-133a, miR-146, miR-31 and Let-7 g) in the peripheral blood of 95 lung cancer patients and 100 matched healthy control individuals. We used RNU48 as a reference gene. RNU48 was identified by geNorm and NormFinder algorithms as the most appropriate for the normalization of the microRNA expression in peripheral blood. The expression level of miR-143 was significantly higher (*p* < 0.0001), whereas the expression level of miR-221 (*p* = 0.000253) was significantly lower, in the blood samples of patients with lung cancer in comparison to the control group. Based on the capacity of these miRNAs to discriminate patient and control samples, we suggest that after further validation, these microRNAs could serve as potential non-invasive diagnostic biomarkers in lung cancer.

Acknowledgements: This work was supported by grants APVV-0412-11 and APVV-15-0217 and by the project Competence Center for Research and Development in the Field of Diagnostics and Therapy of Oncological Diseases, ITMS: 26220220153; the project is co-financed from EU sources.

**Monitoring of activating and resistance mutations in circulating cell-free tumor DNA of non-small cell lung cancer patients**

Restante G^1^, Rofi E^1^, Del Re M^1^, Bordi P^2^, Tiseo M^2^, Danesi R^1^

^1^Clinical Pharmacology and Pharmacogenetics Unit, Dept. of Clinical and Experimental Medicine, University of Pisa, Italy

^2^Medical Oncology Unit, University Hospital of Parma, Italy

*Correspondence: Prof. Romano Danesi, ^1^Clinical Pharmacology and Pharmacogenetics Unit, Dept. of Clinical and Experimental Medicine, University of Pisa, Italy; e-mail: romano.danesi@unipi.it

**Abstract**

**Background.** In non-small cell lung cancer (NSCLC) patients, the efficacy of treatment with EGFR tyrosine kinase inhibitors (EGFR-TKIs) depends on activating EGFR mutations [1]. However, in half of cases, inhibition of EGFR may select resistant cells displaying the EGFR p.T790M point mutation [2].

**Aim.** To monitor the early variation in EGFR mutations in NSCLC patients using circulating cell-free tumor DNA (cftDNA) extracted from plasma.

**Methods.** Seventy-nine NSCLC patients were enrolled in this ongoing explorative study. Six milliliters of plasma was prospectively collected at baseline and at each clinical follow-up. CftDNA was extracted from plasma with the QIAamp Circulating Nucleic Acid Kit (Qiagen®) and analyzed using the Droplet Digital PCR (ddPCR; (BioRad®) for EGFR p.L858R, exon 19 deletions and p.T790M mutations.

**Results.** Forty-four patients testing positive for EGFR activating mutation were treated with the first-generation EGFR-TKIs, while 35 patients testing positive for p.T790M at baseline were treated with AZD9291, the potent, irreversible EGFR-TKI selective for the p.T790M mutation. During the treatment with first-generation TKIs, nine patients experienced clinical progression after a median of 9 months and, testing positive for p.T790M, switched therapy to AZD9291. Interestingly, the appearance of low levels of activating EGFR mutations anticipated the appearance of both high levels of the resistance mutation and clinical progression occurring several months later.

**Conclusions.** CftDNA analysis is useful not only for monitoring clonal evolution of NSCL cancer cells under the stress of EGFR-TKIs, but also in evaluating treatment responses and choosing the more effective therapy in individual patients.

**References**

1. Yang Z, Hackshaw A, Feng Q, et al., Comparison of gefitinib, erlotinib and afatinib in non-small cell lung cancer: A meta-analysis. Int J Cancer. 2017 Jun 15;140(12):2805-2819. doi: 10.1002/ijc.30691.
2. Wang Z, Chen R, Wang S, et al., Quantification and dynamic monitoring of EGFR T790M in plasma cell-free DNA by digital PCR for prognosis of EGFR-TKI treatment in advanced NSCLC. PLoS One. 2014 Nov 18;9(11):e110780. doi:10.1371/journal.pone.0110780.

**Detection and analysis of circulating tumour cells in small cell lung cancer utilizing a flexible rare cell scanning platform**

^1^Kilpatrick MW, ^1^Kershnar ER, ^1^Borgerding RH, ^2,3^Robson P, ^2^Sivakamasundari V.

^1^Ikonisys Inc., New Haven, CT USA

^2^The Jackson Laboratory for Genomic Medicine, Farmington, CT USA

^3^Dept of Genetics & Genome Sciences, UConn School of Medicine, Farmington, CT USA

*Correspondence: Michael W. Kilpatrick, 5 Science Park, New Haven, CT 06032 USA; e.mail: kilpatrick@ikonisys.com

**Keywords**: Circulating tumour cells, Small cell lung cancer, Disease management, Personalized medicine, Rare-cell imaging

**Abstract**

The availability of CTCs as liquid biopsies suggests the potential for studying dynamic change(s) in CTCs during the course of therapy, thus informing both treatment and drug development. Small cell lung cancer (SCLC) has a poor prognosis due to rapid growth, early tumor spread, and unavoidable drug resistance [1]. It has been suggested that the CTC number and persistence in SCLC is highly prognostic, which could be of clinical utility for guiding disease management or in early clinical trials of novel agents [2]. A major challenge in the clinical utilization of CTCs is their rarity, and several approaches have been taken for their enrichment, each with its limitations and with no one clearly superior. We, therefore, developed a completely flexible rare-cell imaging approach that could be applied to CTCs enriched by any desired method. Enrichment can be marker-based, size-based, or microfluidic, or large (two million per slide) numbers of cells can be scanned quickly and efficiently, requiring no enrichment. This approach was able to detect a single tumor cell in one million peripheral blood mononuclear cells (PBMC), with either filtration-based enrichment or no enrichment. CTCs were identified in patients with a variety of cancers [3], utilizing both tumor-specific antibodies and fluorescent in situ hybridization (FISH) markers for the detection of aneuploidy. Analysis of a 2-ml blood sample from an SCLC patient identified eight CTCs (Fig. 1). This uniquely flexible approach permits the interrogation of a CTC for up to five nucleic acid or protein markers, allowing, in addition to enumeration, the molecular characteristics of the detected CTCs to be explored.

**References**

1. Semenova EA, Nagel R, Berns A. Origins, genetic landscape, and emerging therapies of small cell lung cancer. Genes Dev. 2015;15;29(14):1447-62. doi:10.1101/gad.263145.115.
2. Naito T, Tanaka F, Ono A, Yoneda K, Takahashi T, Murakami H et al. Prognostic impact of circulating tumor cells in patients with small cell lung cancer. J Thorac Oncol. 2012;7(3):512-9. doi:10.1097/JTO.0b013e31823f125d.
3. Ntouroupi TG, Ashraf SQ, McGregor SB, Turney BW, Seppo A, Kim Y et al. Detection of circulating tumour cells in peripheral blood with an automated scanning fluorescence microscope. Br J Cancer. 2008;2;99(5):789-95. doi:10.1038/sj.bjc.6604545.

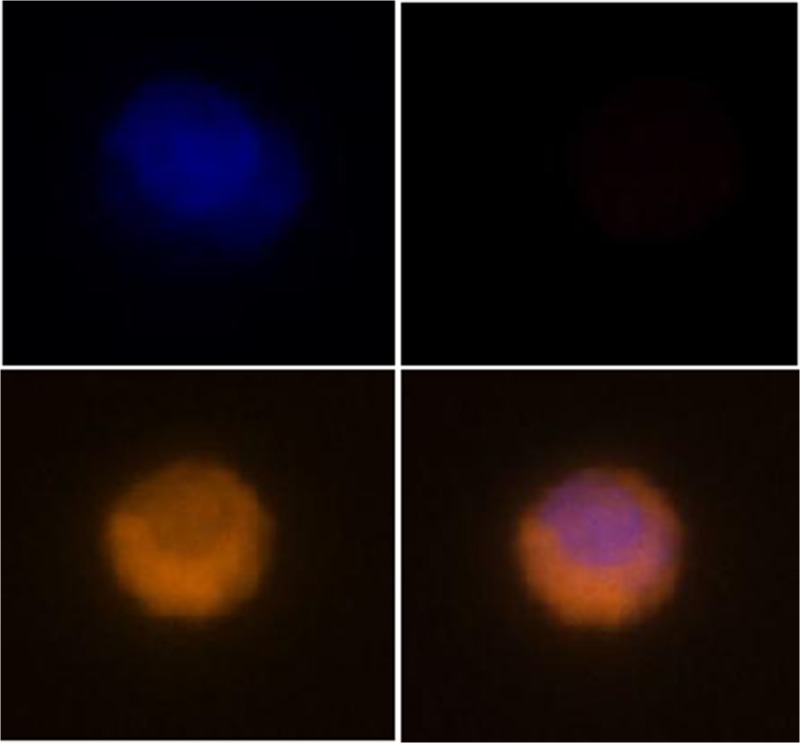


**Fig. 1** CTC detected in an SCLC patient based on the presence of a DAPI-stained nucleus (top left), expression of EpCAM (bottom left), and lack of CD45/CD31 expression (top right). A composite image of the DAPI, EpCAM, and CD45/CD31 channels is shown at the bottom right.

**Immune checkpoint inhibitors in the treatment of patients with glioblastoma multiforme: from preclinical research to early clinical results**

Jiri Polivka jr. *^1,2,3^, Jiri Polivka^3^, Milena Kralickova^1^, Lubos Holubec ^2^

^1^Department of Histology and Embryology, Faculty of Medicine in Plzen, Charles University, Czech Republic

^2^Biomedical Center, Faculty of Medicine in Plzen, Charles University, Czech Republic

^3^Department of Neurology, Faculty Hospital Plzen, Czech Republic

*Correspondence: Jiri Polivka jr., Ph.D., Department of Histology and Embryology, Faculty of Medicine in Plzen, Charles University, Karlovarska 48, 301 66 Plzen, Czech Republic; e.mail: polivkajiri@gmail.com

**Keywords**: Personalized medicine, Glioblastoma multiforme, CTLA4 inhibition, PD1 inhibition, Immune checkpoint inhibitors, Clinical trials

**Abstract**

Glioblastoma multiforme (GBM) represents one of the most malignant tumors of the primary central nervous system in adults, with considerably high mortality [1,2]. Standard therapies including neurosurgery, radiotherapy and chemotherapy with temozolomide have only a limited effect on the long-term survival of GBM patients [3,4]. A number of targeted anti-cancer therapeutics, including low-molecular-weight tyrosine kinase inhibitors or monoclonal antibodies, have been evaluated in numerous clinical trials with newly diagnosed as well as recurrent GBM. Unfortunately, until today, only one targeted anti-tumor drug – the antiangiogenic inhibitor bevacizumab – has been approved for the treatment of recurrent GBM, albeit not in the EU [5]. Moreover, its effect on the overall survival of patients with recurrent GBM remains controversial. Major advances in cancer immunotherapy over the past few years now offer hope for the possible future use of such treatment strategies for patients with GBM as well [6,7]. This presentation will discuss the recent preclinical and early clinical results from studies evaluating anti-tumor immunotherapy using so-called immune checkpoint inhibitors that might improve the very unfavorable prognosis of patients with GBM in the near future. This work was supported by MH CZ – DRO (Faculty Hospital Plzen - FNPl, 00669806) and by the National Sustainability Program I (NPU I) Nr. LO1503 provided by the Ministry of Education, Youth and Sports of the Czech Republic, and by the Charles University Research Fund (Progres Q39).

**References**

1. Louis DN, Perry A, Reifenberger G, von Deimling A, Figarella-Branger D, Cavenee WK, et al. The 2016 World Health Organization Classification of Tumors of the Central Nervous System: a summary. Acta Neuropathol. (Berl.). 2016;131:803–20.
2. Dolecek TA, Propp JM, Stroup NE, Kruchko C. CBTRUS statistical report: primary brain and central nervous system tumors diagnosed in the United States in 2005-2009. Neuro-Oncol. 2012;14 Suppl 5:v1-49.
3. Stupp R, Mason WP, van den Bent MJ, Weller M, Fisher B, Taphoorn MJB, et al. Radiotherapy plus concomitant and adjuvant temozolomide for glioblastoma. N. Engl. J. Med. 2005;352:987–96.
4. Tosoni A, Franceschi E, Poggi R, Brandes AA. Relapsed Glioblastoma: Treatment Strategies for Initial and Subsequent Recurrences. Curr. Treat. Options Oncol. 2016;17:49.
5. Polivka J, Polivka J, Holubec L, Kubikova T, Priban V, Hes O, et al. Advances in Experimental Targeted Therapy and Immunotherapy for Patients with Glioblastoma Multiforme. Anticancer Res. 2017;37:21–33.
6. Preusser M, Lim M, Hafler DA, Reardon DA, Sampson JH. Prospects of immune checkpoint modulators in the treatment of glioblastoma. Nat. Rev. Neurol. 2015;11:504–14.
7. Huang B, Zhang H, Gu L, Ye B, Jian Z, Stary C, et al. Advances in Immunotherapy for Glioblastoma Multiforme. J. Immunol. Res. 2017;2017:3597613.

**Biomarker individual repair capacity to susceptibility in colorectal cancer**

Tatiana Matakova^1,2^, Erika Halašova^1,3^, Anton Dzian^4^, Maria Skerenová^5^ and Dusan Dobrota^2^

^1^Comenius University in Bratislava, Jessenius Faculty of Medicine in Martin, Biomedical Center Martin, Department of Molecular Medicine, Mala Hora 11161/4C Martin, Slovakia

^2^Comenius University in Bratislava, Jessenius Faculty of Medicine in Martin, Department of Medical Biochemistry, Mala Hora 4D, 03601 Martin, Slovakia

^3^Comenius University in Bratislava, Jessenius Faculty of Medicine in Martin, Department of Medical Biology, Mala Hora 4, 03601 Martin, Slovakia

^4^Comenius University in Bratislava, Jessenius Faculty of Medicine in Martin, Clinic of Thoracic Surgery, University Hospital in Martin, Kollarova 2, 03659, Martin, Slovakia

^5^University Hospital in Martin, Department of Clinical Biochemistry, Kollarova 2, 03659 Martin, Slovakia

*Correspondence: Dr. Tatiana Matakova, E mail: matakova@jfmed.uniba.sk

**Keywords:** Colorectal cancer, Biomarkers, Individual repair capacity, Susceptibility

**Abstract**

Most colorectal cancers are considered sporadic, and often the cause is not known. Biomarkers of individual repair capacity (IRC) play a role not only in the risk of developing cancer, but also in virtually every aspect of the course of the disease.

In our case–control study of 273 colorectal cancer patients and 187 cancer-free controls, the 22 single-nucleotide polymorphisms (SNPs) in 12 genes (*GSTP1*, *CYP1A2*, *CYP1B1*, *CYP2C8*, *CYP2C9*, *CYP2E1*, CYP2W1*2, *CYP19A, MTHFR, XRCC1, hOOG1, DPYD)* were genotyped and analyzed for their correlation with the risk of sporadic colorectal cancer in multivariate logistic regression models. There were significant genotype associations of the following SNPs: rs1695 GSTP1, rs1801133 and rs1801131 MTHFR, rs25487 XRCC1 rs1052133 hOOG1 and rs1801160 and 1801159 DPYD. Haplotype analysis of four *DPYD* polymorphisms showed significant differences in the distribution of the IISt haplotype between cases and controls. In comparison to the most common haplotype (VISt), the IISt haplotype was associated with increased risk of colorectal cancer (*p* = 0.038, OR = 2.733, 95% CI = 1.019–7.326).

**Acknowledgements:** This work was supported by the project Competence Centre for Research and Development in the Field of Diagnostics and Therapy of Oncological Diseases, ITMS: 26220220153, the project is co-financed from EU sources.

**Unravelling the role of** ***TRIB1*** **in colorectal cancer: a functional molecular pathology approach**

Romina Briffa*^1,2^, Inhwa Um^1^, Peter Mullen^1^, Paul Reynolds^1^, David J Harrison^§1^ and Godfrey Grech^§2^

^1^School of Medicine, University of St Andrews, Fife KY16 9TF, United Kingdom

^2^Department of Pathology, Faculty of Medicine and Surgery, University of Malta, Msida MSD2080, Malta

^§^Both authors contributed equally to this work.

*Correspondence: Dr Romina Briffa, Department of Pathology, Faculty of Medicine and Surgery, University of Malta, Msida Malta; e.mail: rbrif01@um.edu.mt; rb228@st-andrews.ac.uk

**Keywords**: Precision medicine, Colorectal cancer, Multi-scale analysis, Patient stratification, Prognostic biomarkers

**Abstract**

Despite recent advances in clinical and experimental colorectal cancer (CRC) research, the optimal approach for stratifying CRC patients remains uncertain. We previously described amplification of *TRIB1* and *MYC* in 14.5% and 7.4% of a CRC cohort (*n*=118), respectively, and found these amplifications significantly correlated (*p* = 0.0001) in a number of cases^1^. Our current aims are (i) to understand the prognostic role and function of *TRIB1* in CRC, and ii) to identify a clinical and molecular signature for this subgroup of patients that can be used for prognostication and therapeutic targeting. To this end, we are validating *TRIB1* expression/amplification in 500 Maltese patients diagnosed with CRC between 2008 and 2011. A tissue microarray platform was used to quantify gene amplification by fluorescence in situ hybridisation (FISH) and protein expression by immunofluorescence imaging. TRIB1 expression will be associated with several clinicopathological parameters. CRISPR/Cas9 technology was used to create three stable *TRIB1* knock-down CRC cell lines, which were used for functional analysis using the Celigo® image cytometer to measure cell cycle progression, proliferation, tumour growth, and invasion. Western blotting will be used to interrogate several pathways including MAPK, PI3K/AKT, NF-κB, cell cycle, and apoptosis. Our data show that Trib1 protein expression in the patient cohort is significantly positively correlated with Akt, BRCA1, Met, Erk, Stat3, MEK, Myc, caspase 3, PTEN, Stat3, and CDK2 (*p* =<0.05). These findings will fully characterise the *TRIB1*-positive subgroup, laying the foundation for therapeutic targeting and companion diagnostics. Correct patient stratification increases patient survival, reduces recurrence, and minimises overtreatment.

**References**

1. Briffa R, Um I, Faratian D, Zhou Y, Turnbull AK, et al. (2015) Multi-Scale Genomic, Transcriptomic and Proteomic Analysis of Colorectal Cancer Cell Lines to Identify Novel Biomarkers. PLOS ONE 10(12): e0144708. doi: 10.1371/journal.pone.0144708.

**Modulation of the immune component of the tumor microenvironment: perspectives for personalized cancer therapy**

Julia Kzhyshkowska^1,2,3^, Irina Mitrofanova^3,4^, Tengfei Liu^1^, Marina Zavyalova^3,4^, Nikolai Litvyakov^3,4^, Mikhail Buldakov^3,4^, Nadezhda Cherdyntseva^3,4.^

^1^University of Heidelberg, Medical Faculty Mannheim, Institute of Transfusion Medicine and Immunology, Mannheim, Germany

^2^German Red Cross Blood Service Baden-Württemberg – Hessen, Mannheim, Germany

^3^Tomsk State University, Tomsk, Russia

^4^Tomsk National Research Medical Center of the Russian Academy of Sciences, Tomsk, Russia

*Correspondence: Prof. Dr. Julia Kzhyshkowska, Institute of Transfusion Medicine and Immunology, Medical Faculty Mannheim, Heidelberg University; Ludolf-Krehl Strasse 13-17, D-68167 Mannheim; e.mail: julia.kzhyshkowska@medma.uni-heidelberg.de

**Abstract**

The increasing number of cancer cases and high cancer-related mortality is a global problem that requires huge financial investments in both diagnostics and therapeutic interventions. The most challenging issue for cancer therapy is the development of personalized approaches. Despite recent advances in our understanding of fundamental aspects of cancer cell biology and the development of anti-cancer therapeutic approaches, accumulating evidence indicates that efficient anti-cancer personalized therapy is possible only if we consider the role of the immune component of the tumor microenvironment in carcinogenesis (1). Another essential factor that defines individual responses to chemotherapy is intratumoral heterogeneity. This is caused by genomic instability in cancer cells, resulting in the selection of resistant clones that can differentially interact with the immune component of the immune tumor microenvironment (2). Moreover, we have recently found that compartmentalization of the immune component in functionally different intratumoral areas is predictive of metastasis efficiency after neoadjuvant chemotherapy in breast cancer patients (3). Targeting specific immune components of the tumor microenvironment is a promising treatment strategy that can improve the outcome of conventional anti-cancer therapy. Modern immunotherapeutic approaches are based on targeting of tumor-infiltrating immune cells, including neutrophils, dendritic cells, NK cells, T cells, B cells, and macrophages. The most abundant immune cells in the tumor microenvironment are tumor-associated macrophages (TAM) that control tumor growth, vascularisation, and metastatic spread (4). TAM display a pronounced heterogeneity and phenotypic plasticity that offers the possibility of using them as biomarkers for predicting individual patient responses to therapy, and stratification of patients towards specific therapeutic schemas. TAM intra-tumor heterogeneity and the existence of patient-specific phenotype signatures form the basis for the development of novel personalized TAM-based therapeutic approaches.

Funding: Russian Science Foundation №14-15-00350.

**References**

1. Anderson KG, Stromnes IM, Greenberg PD. Obstacles Posed by the to Tumor Microenvironment to T cell Activity: A Case for Synergistic Therapies. Cancer Cell. 2017 Mar 13;31(3):311-325. doi:10.1016/j.ccell.2017.02.008.
2. McGranahan N, Swanton C. Clonal Heterogeneity and Tumor Evolution: Past, Present, and the Future. Cell. 2017 Feb 9;168(4):613-628. doi:10.1016/j.cell.2017.01.018.
3. Mitrofanova I, Zavyalova M, Telegina N, Buldakov M, Riabov V, Cherdyntseva N, Kzhyshkowska J. Tumor-associated macrophages in human breast cancer parenchyma negatively correlate with lymphatic metastasis after neoadjuvant chemotherapy. Immunobiology. 2017 Jan;222(1):101-109. doi:10.1016/j.imbio.2016.08.001.
4. Riabov V, Gudima A, Wang N, Mickley A, Orekhov A, Kzhyshkowska J**.**. 28. Role of tumor associated macrophages in tumor angiogenesis and lymphangiogenesis. Front Physiol. 2014 Mar 5;5:75. doi:10.3389/fphys.2014.00075.

**Evaluation of cytotoxicity of nanoparticles on cancer cells with LDH and MTT assays**

Orestis Strymponis

Biomedical Science, University of Bradford, UK

**Abstract**

Nanoparticles are gaining increasing popularity as medical tools against cancer. Their applications range from diagnostic purposes to possible therapeutic interventions. Although the ongoing research has revealed some promising results, a serious manifestation of nanoparticle-induced adverse effects has been reported by many researchers. Thus, the evaluation of the cytotoxicity that nanoparticles exert on cells must be thoroughly examined. This study focuses on the response of two cancer cell lines, HeLa cervical cancer and H460 lung cancer cells, to zinc oxide, gold and silicon dioxide nanoparticles. Cells were exposed to various doses over a 24-h period. Cytotoxicity levels were then evaluated by MTT and LDH cytotoxic assays. Results showed reduced cell viability over both cell lines and for all nanoparticle treatments. However, cytotoxicity levels varied, with correlations to dose-, nanoparticle- or cell-dependent cytotoxicity only in specific aspects of the experiment. Although further research is needed to reveal the full potential, along with the complete range of side effects, of nanoparticles, the versatile performance of these nanoscale materials can be a powerful tool for researchers. Thus far, gold nanoparticles have been the most popular in the modern face of personalised medicine, with applications in imaging, drug delivery and enhancement of conventional therapeutic strategies. In addition, zinc oxide and silica nanoparticles have been used in cancer diagnostics and therapeutics as well as imaging and drug monitoring, applications that make them promising candidates for individually targeted medicine [1-5].

**References**

1. Bhattacharya, D., Bhattacharyya, A. and Karmakar, P. Evaluation of Different Oxidative Stress Parameters and Apoptosis in Human Cervical Cancer Cells Exposed to Rod and Spherical Shaped Zinc Oxide Nanoparticles. BioNanoScience 2016;6(1): 1-14.
2. Choi, S. Y., Jang, S. H., Park, J., Jeong, S., Park, J. H., Ock, K. S., Lee, K., Yang, S. I., Joo, S.-W., Ryu, P. D. and Lee, S. Y. (2012) Cellular uptake and cytotoxicity of positively charged chitosan gold nanoparticles in human lung adenocarcinoma cells. Journal of Nanoparticle Research 2012;14(12): 1234.
3. Cui, W., Li, J., Zhang, Y., Rong, H., Lu, W. and Jiang, L. Effects of aggregation and the surface properties of gold nanoparticles on cytotoxicity and cell growth. Nanomedicine: Nanotechnology, Biology and Medicine 2012;8(1): 46-53.
4. Hong, H., Wang, F., Zhang, Y., Graves, S. A., Eddine, S. B. Z., Yang, Y., Theuer, C. P., Nickles, R. J., Wang, X. and Cai, W. Red Fluorescent Zinc Oxide Nanoparticle: A Novel Platform for Cancer Targeting. ACS Applied Materials & Interfaces 2015;7(5):3373-3381.
5. Kim, I.-Y., Joachim, E., Choi, H. and Kim, K. Toxicity of silica nanoparticles depends on size, dose, and cell type. Nanomedicine: Nanotechnology, Biology and Medicine 2015;11(6): 1407-1416.

**PPPM IN NEUROLOGICAL, NEURODEGENERATIVE AND NEUROPSYCHIATRIC DISORDERS**

**Psychiatry – Quo Vadis? Opportunities for a personalised approach to diagnosis and treatment**

Bernhard T. Baune

Discipline of Psychiatry, Adelaide Medical School, University of Adelaide, Australia

*Correspondence: Prof. Dr. Bernhard T. Baune, Discipline of Psychiatry, University of Adelaide, North Terrace, 5000 Adelaide, Australia

**Keywords:** Personalized psychiatry, Prevention, Prediction, Decision-making process, Biomarker, Treatment, Diagnosis

**Abstract**

Psychiatry as a clinical discipline is largely driven by a symptom-based approach that underpins current diagnostic (e.g. DSM-V) and therapeutic decision-making processes. Reliable biological markers are largely absent from current diagnosis and treatment. In addition, evaluation of treatment efficacy rests solely on patient self-report and clinical judgement. Does psychiatry as a field have the opportunity to develop beyond a clinically driven “wait-and-see” approach [1]? In this presentation, the concept of personalized psychiatry will be introduced, which integrates both biological systems and clinical markers, incorporating prevention, prediction and participation as key features of personalised medicine.

The identification of distinct clinical and biological disease trajectories underscores a prospective and proactive preventive approach to mental illness for a large proportion of people at risk for a first or for subsequent episodes of mental illness [2,3]. Pre-treatment stratification into meaningful subgroups of patients is a necessary part of personalized psychiatry, in order to inform more effective treatment decisions [4]. A specific biologically determined clinical subgroup of patients with depressive symptomatology consists of patients presenting with increased peripheral inflammatory markers that may make these suitable diagnostic markers for clinical stratification into specific treatment groups. In addition, the genomics of treatment response to pharmacological and psychological interventions is crucial for a personalized approach in psychiatry. The prospective and predictive approach taken in personalized psychiatry requires bioinformatics modelling that considers both clinical and biological markers for more accurate prediction of disease onset and disease course, as well as prediction of treatment needs. Using our multimodal probabilistic prediction model, prediction of treatment needs in psychosis have been illustrated [5].

The outlined concept of personalized psychiatry as illustrated by several examples comprehensively describes key ingredients needed for personalizing the diagnosis and treatment of patients with psychiatric disorders. A clinical experimental validation process is essential and among the key challenges in personalized psychiatry for the translation of findings into clinical practice and guidelines and for patient participation.

**References**

1. Baune BT. Taking a look into the future: Do clinical and neurobiological trajectories tell us more than what we already know? Aust N Z J Psychiatry. 2017;51(5):429-430.
2. Schubert KO, Clark SR, Baune BT. The use of clinical and biological characteristics to predict outcome following First Episode Psychosis. Aust N Z J Psychiatry. 2015;49(1):24-35.
3. Schubert KO, Clark SR, Van LK, Collinson JL, Baune BT. Depressive symptom trajectories in late adolescence and early adulthood: A systematic review. Aust N Z J Psychiatry. 2017;51(5):477-499.
4. Clark SR, Schubert KO, Baune BT. Towards indicated prevention of psychosis: using probabilistic assessments of transition risk in psychosis prodrome. J Neural Transm (Vienna). 2015;122(1):155-69.
5. Clark SR, Baune BT, Schubert KO, Lavoie S, Smesny S, Rice SM, Schäfer MR, Benninger F, Feucht M, Klier CM, McGorry PD, Amminger GP. Prediction of transition from ultra-high risk to first-episode psychosis using a probabilistic model combining history, clinical assessment and fatty-acid biomarkers. Transl Psychiatry. 2016;6(9):e897

**Retinal imaging in neuroinflammatory and neurodegenerative disorders: current research and clinical applications**

Friedemann Paul^1,2,3^

^1^ NeuroCure Clinical Research Center, Charité – University Medicine Berlin, Berlin, Germany

^2^ Clinical and Experimental Multiple Sclerosis Research Center, Department of Neurology, Charité – University Medicine Berlin, Berlin, Germany

^3^ Experimental and Clinical Research Center, Max Delbrueck Center for Molecular Medicine and Charité – University Medicine Berlin, Berlin, Germany

*Correspondence: Prof. Dr. Friedemann Paul, NeuroCure Clinical Research Center, Charité – University Medicine Berlin, Charitéplatz 1, 10117 Berlin, Germany, email: friedemann.paul@charite.de

**Keywords:**Neurodegeneration, Neuroinflammation, Retina, Optical coherence tomography, Disease prediction, Personalized medicine

**Abstract**

Imaging of the retina with the help of high-resolution spectral domain optical coherence tomography (OCT) has leveraged both clinical and preclinical investigations of inflammatory, autoimmune and neurodegenerative processes that affect retinal tissue and the anterior part of the visual pathway. In combination with (ultrahigh-field) MRI, a thorough investigation of the entire visual pathway from the retina to the visual cortex is feasible. Over the past 10 years, our understanding of the pathophysiology of retinal damage, and how this relates to global disease burden and clinical disability in neuroimmunological and neurodegenerative diseases such as optic neuritis, multiple sclerosis, neuromyelitis optica, Susac syndrome, Parkinson’s disease, and dementias has increased substantially. Consequently, OCT is increasingly used to assess patients with autoimmune and neurodegenerative conditions of the central nervous system in clinical routine, and the technique is also implemented as an outcome tool in clinical trials investigating the visual system. In preclinical research, retinal imaging by OCT is used in animal models to deepen our understanding of the pathophysiologic mechanisms prevailing in these conditions. Both preclinical data and clinical applications of retinal imaging techniques in relation to MRI findings in various conditions will be presented, and future research questions with regard to differential diagnosis and individualized treatment approaches will be discussed.

**References**

1. Martinez-Lapiscina EH, Arnow S, Wilson JA, Saidha S, Preiningerova JL, Oberwahrenbrock T, Brandt AU, Pablo LE, Guerrieri S, Gonzalez I, Outteryck O, Mueller AK, Albrecht P, Chan W, Lukas S, Balk LJ, Fraser C, Frederiksen JL, Resto J, Frohman T, Cordano C, Zubizarreta I, Andorra M, Sanchez-Dalmau B, Saiz A, Bermel R, Klistorner A, Petzold A, Schippling S, Costello F, Aktas O, Vermersch P, Oreja-Guevara C, Comi G, Leocani L, Garcia-Martin E, Paul F, Havrdova E, Frohman E, Balcer LJ, Green AJ, Calabresi PA, Villoslada P; IMSVISUAL consortium. Retinal thickness measured with optical coherence tomography and risk of disability worsening in multiple sclerosis: a cohort study. Lancet Neurol. 2016;15(6):574-84.
2. Oertel FC, Kuchling J, Zimmermann H, Chien C, Schmidt F, Knier B, Bellmann-Strobl J, Korn T, Scheel M, Klistorner A, Ruprecht K, Paul F, Brandt AU. Microstructural visual system changes in AQP4-antibody-seropositive NMOSD. Neurol Neuroimmunol Neuroinflamm. 2017;4(3):e334.
3. Pache F, Zimmermann H, Mikolajczak J, Schumacher S, Lacheta A, Oertel FC, Bellmann-Strobl J, Jarius S, Wildemann B, Reindl M, Waldman A, Soelberg K, Asgari N, Ringelstein M, Aktas O, Gross N, Buttmann M, Ach T, Ruprecht K, Paul F, Brandt AU; in cooperation with the Neuromyelitis Optica Study Group (NEMOS). MOG-IgG in NMO and related disorders: a multicenter study of 50 patients. Part 4: Afferent visual system damage after optic neuritis in MOG-IgG-seropositive versus AQP4-IgG-seropositive patients. J Neuroinflammation. 2016;13(1):282.
4. Sinnecker T, Kuchling J, Dusek P, Dörr J, Niendorf T, Paul F, Wuerfel J. Ultrahigh field MRI in clinical neuroimmunology: a potential contribution to improved diagnostics and personalised disease management. EPMA J. 2015;6(1):16.
5. Kuchling J, Brandt AU, Paul F, Scheel M. Diffusion Tensor Imaging for multi-level assessment of the visual pathway: possibilities for personalized outcome prediction in autoimmune disorders of the central nervous system. EPMA J 2017; 8(3).

**Machine-assisted cognitive bias correction and prediction are key to predictive, preventive, personalised medicine (PPPM)**

Michael Legg

School of Medical Sciences, University of New South Wales, Sydney, Australia

*Correspondence: Michael Legg & Associates, 33G Hospital Road, Bulli NSW 2516, Australia; email: michael.legg@mlanda.com.au

**Keywords**: Predictive, preventive, personalised medicine, Cognitive bias, Prediction, Informatics

**Abstract**

Proactive healthcare relies on the capacity to detect health risk and early disease states. The recent Institute of Medicine Report on diagnosis has identified diagnosis as a major source of error in the health system, even for established disease [1]. Diagnosis and the decisions that follow rely on good judgement – often involving the assessment of probability and comparisons of predictions over time for alternate scenarios.

Work in psychology around cognitive bias, heuristics and decision-making show that humans are poor at these.

Even the well-trained routinely fail at assessing probability and at predicting the outcome of even simple scenarios over time. Recognition of these weaknesses and training against them does not fix them – we seem unable to correct for our own biases. Indeed, it has been shown in well-constructed experiments that our confidence rises as we are more likely to predict the wrong outcome [2, 3].

Informatics and its machines are important in all aspects of proactive health care, but helping to accommodate for the way that people think offers an important opportunity for the advancement of predictive, preventive, personalised medicine (PPPM) [4]. Machine assistance can help people avoid likely errors and correct for biases that will lead to a less rational outcome, but using the way that people think in a constructive way, they can also be used to reinforce the most appropriate interpretation and action.

Scientific medicine could be made much easier, and informatics and its machines can help in new ways.

**References**

1. National Academies of Sciences, Engineering, and Medicine: Improving diagnosis in health care. Washington, DC: The National Academies Press. 2015.
2. Kahneman, D: Thinking Fast and Slow. New York: Farrar, Straus and Giroux. 2011.
3. Lewis, M: The Undoing Project: A Friendship That Changed Our Minds. New York: WW Norton. 2016.
4. Bodrova, T.A., Kostyushev, D.S., Antonova, E.N., Slavin, S., Gnatenko, D.A., Bocharova, M.O., Legg, M., Pozzilli, P Paltsev M.A., Suchkov, S.V.: Introduction into PPPM as a new paradigm of public health service: an integrative view. EPMA J. 2012; 3: 16. doi:10.1186/1878-5085-3-16

**Personalized approach in the treatment of tinnitus and insomnia: combining repetitive transcranial magnetic stimulation and cognitive behavioral therapy**

Kneginja Richter*^1,2,3^, Lukas Peter^1^, Jens Acker^4^, Joachim Höfig^1^, Lence Miloseva^2^ and Günter Niklewski^1^

^1^University Clinic for Psychiatry and Psychotherapy, Paracelsus Medical University Nuremberg, Germany

^2^Faculty of Medical Science, Goce Delčev University of Štip, R.Macedonia

^3^Faculty of Social Sciences, Georg-Simon-Ohm University of Applied Sciences, Nuremberg, Germany

^4^Clinic for Sleep Medicine Zurzach, Switzerland

*Correspondence: Kneginja Richter, University Clinic for Psychiatry and Psychotherapy, Paracelsus Medical University Nuremberg, Prof.-Ernst-Nathan-Str. 1, 90419 Nürnberg, Germany; e.mail: Kneginja.Richter@gmx.de

**Keywords:** Predictive preventive personalized medicine, Tinnitus, Insomnia, Sleep, Repetitive transcranial magnetic stimulation, Cognitive behavioral therapy

**Abstract**

We present the case of a 53-year-old male patient who had been suffering from symptoms of decompensated and chronified tinnitus for 4 years [1], most likely caused by work stress. In addition, the patient developed comorbid decompensated insomnia. Because of potential bidirectional connections between tinnitus and sleep disorders, an interdisciplinary approach to treatment was chosen. The treatment plan we developed consisted of ten sessions of repetitive transcranial magnetic stimulation (rTMS) for tinnitus [2], followed by ten sessions of cognitive behavioral therapy (CBT) for tinnitus and insomnia. We used the Tinnitus Questionnaire (TF) to assess tinnitus severity, the Beck Depression Inventory (BDI-II) for depressive symptoms, and the WHO Well-Being Index (WHO-5) for subjective well-being. Improvements were achieved with regard to everyday functioning, as the patient went from decompensated and severe to clinically negligible TF scores, from minimal to no depressive symptoms, and from just above critical to above average well-being. Combining equipment-based and psychological approaches to treatment proved successful in this case. We conclude that a combination of rTMS and CBT may be considered as an effective treatment for chronic tinnitus and comorbid sleep disorders.

**References**

1. Richter K, Acker J, Miloseva L, Peter L, Niklewski G. Management of Chronic Tinnitus and Insomnia with Repetitive Transcranial Magnetic Stimulation and Cognitive Behavioral Therapy – a Combined Approach. Front Psychol. 2017;8:575. doi:10.3389/fpsyg.2017.00575.
2. Langguth B, Kreuzer PM, Kleinjung T, DeRidder D. Tinnitus: Causes and Clinical Management. Lancet Neurol. 2013;12: 920.

**Prediction and prevention of suicidality among patients with depressive disorders: comorbidity as a risk factor**

Lence Miloseva *^1^, Vladimir Milosev ^1,2^, Kneginja Richter ^1,3,4^, Lukas Peter ^3^, Günter Niklewski ^1,3^

^1^Faculty of Medical Science, Goce Delcev University, Stip, R.Macedonia

^2^Clinical Hospital Stip, R. Macedonia

^3^University Clinic for Psychiatry and Psychotherapy, Paracelsus Medical University, Nuremberg, Germany

^4^ Technische Hochschule Nuernberg Georg Simon Ohm

*Correspondence: Prof. Dr. Lence Miloseva, Faculty of Medical Science, Goce Delcev University, Stip, Krste Misirkov 10-A, 2000 Stip, R.Macedonia; e-mail: lence.miloseva@ugd.edu.mk

**Keywords:** Prediction, Prevention, Suicidality, Depressive disorders, Comorbid disorders

**Abstract**

The aim of this paper is twofold. The first to assess the role of comorbidity as a risk factor in predicting suicidality among patients with depressive disorders and those with comorbidity or dual diagnosis. The second is to discuss implications for prevention.

Within the framework of the UGD-supported project, the data were collected from Clinical Hospital, Stip, Macedonia, during the period January 2015 to March 2017. The sample consists of 140 patients (64% female and 36% male) aged 19–72 years. The respondents were divided on the basis of mono and comorbid diagnosis into four subgroups: 1) those with depressive disorder, 2) those with depressive disorder and physical illness, 3) those with depressive disorder and another mental disorder, and 4) those with depressive disorder, physical illness and mental disorder. Data were collected using questionnaires about sociodemographic data, structured interviews and medical documentation, while suicidal behavior was studied using the C-SSRS scale [1,2]. Written informed consent was obtained from all study participants.

The specific focus was on examining the relationship of suicidal thoughts with gender and comorbidity, frequency and intensity, as well as differences in preparation, trials, methods and number of suicides. Additionally, we examined the characteristics of suicide in 46 respondents and whether there was a difference among groups in terms of suicidal behavior. The results are in line with our expectations, and they show that comorbidity is a significant factor in predicting suicidal behavior [3-5]. A detailed statistical analysis is reported in the paper.

Although we are talking about a small sample, at the level of absolute numbers and percentages, where comorbidity is a clinical reality and suicides are related to the number of diagnoses, a larger number of diagnoses leads to a greater risk of suicidal behavior.

**References**

1. Columbia University Medical Center, Columbia-Suicide Severity Rating Scale.2014; Available from: http://cssrs.columbia.edu/the-columbia-scale-c-ssrs/cssrs-for-research/
2. Posner K at all. The Columbia–Suicide Severity Rating Scale: Initial Validity and Internal Consistency Findings From Three Multisite Studies With Adolescents and Adults. Am J Psychiatry. 2011; 168(12):1266-1277.
3. World Health Organization. Suicide prevention (SUPRE).Geneva, Switzerland:World Health Organization. 2007; Available from:
4. http://WWW.who.int/mental_health/prevention/suicide/suicideprevent/en/index.html
5. Gonda X, Fountoulakis KN, Kaprinis G, Rihmer Z. Prediction and prevention of suicide in patients with unipolar depression and anxiety. Ann Gen Psychiatry. 2007; 6:23.
6. Ferro MA. Major depressive disorder, suicidal behaviour, bipolar disorder, and generalised anxiety disorder among emerging adults with and without chronic health conditions. Epidemiol Psychiatr Sci. 2016;25(5):462-474.

**Individualised paediatric drug therapy of an antiepileptic drug**

Hana Shabbi^1^, Christian Saliba^2^, Doriette Soler^3^, Godfrey Grech^4^, and Janet Mifsud^5^

^1^ University of Tripoli and Department of Clinical Pharmacology and Therapeutics, University of Malta, Msida MSD 2080, Malta;

^2^Centre for Molecular Medicine and Biobanking, University of Malta, Msida MSD 2080, Malta;

^3^Department of Paediatrics, Mater Dei Hospital, Malta;

^4^Department of Pathology, University of Malta, Msida MSD 2080, Malta;

^5^Department of Clinical Pharmacology and Therapeutics, University of Malta, Msida MSD 2080, Malta.

*Correspondence: Hana Shabbi, University of Tripoli and Department of Clinical Pharmacology and Therapeutics, University of Malta, Msida MSD 2080, Malta**;** e-mail: hana.shabbi.01@um.edu.mt

**Keywords**: Lamotrigine, Metabolism, Pharmacogenetics, Personalised medicine

**Abstract**

Lamotrigine is a novel antiepileptic drug metabolised by UGT enzymes, mainly UGT1A4 [1,2] and UGT2B7 [2]. Genetic polymorphisms of UGTs result in different phenotypes by affecting the expression levels or activity of individual UGT enzymes, hence variability in drug metabolism and elimination [3,4,5]. Thus, pharmacogenetics is extremely useful in the individualisation of lamotrigine drug therapy. Moreover, paediatric populations with epilepsy offer a specific challenge in dose individualisation, since they undergo significant developmental changes from childhood to adulthood [6]. The aim of this study was to investigate the influence of genetic polymorphisms of UGT1A4 and UGT2B7 on drug concentration in Maltese paediatric patients with epilepsy.

Blood samples from 18 Maltese paediatric patients were collected. Genomic DNA was extracted using well-established standard protocols. Primers were designed to span the entire coding sequence of the UGT1A4 and UGT2B7 genes. DNA amplification of the specific genes was performed by PCR on the respective patient samples. Gel electrophoresis was used to confirm that the desired DNA fragment was obtained. The PCR products were then purified and sequenced using the Sanger sequencing method.

Various mutations were identified in both genes of interest, leading to amino acid changes in the majority of patients. This may result in inter-individual variability in the pharmacokinetics of lamotrigine.

This research will lead to improved therapeutic outcomes for paediatric populations with epilepsy, by providing a greater understanding of the contribution of pharmacogenetics to the individualisation of drug therapy and development of personalised medicine.

**References**

1. Argikar UA, and Remmel RP. Variation in glucuronidation of lamotrigine in human liver microsomes. Xenobiotica. 2009; 39(5): 355–363.
2. Rowland A, Elliot DJ, Williams JA, Mackenzie PI, Dickinson RG, Miners JO. In vitro characterization of lamotrigine N2-glucuronidation and the lamotrigine-valproic acid interaction. Drug metabolism and disposition. 2006; 34(6): 1055-1062.
3. Limin L, Limei Z, Qiuning W, Feng Q, Xiujun W, and Yanan M. Influence of valproic acid concentration and polymorphism of UGT1A4*3, UGT2B7 -161C>T and UGT2B7*2 on serum concentration of lamotrigine in Chinese epileptic children. European Journal of Clinical Pharmacology. 2015; 71:1341–1347.
4. Sánchez MB, Herranz JL, Leno C, Arteaga R, Oterino A, Elsa M, Valdizán EM, Nicolas JM, Adín J, Shushtarian M, Armijo JA. UGT2B7_2161C.T polymorphism is associated with lamotrigine concentration-to-dose ratio in a multivariate study. Therapeutic Drug Monitoring. 2010; 32(2): 177-184.
5. Gulcebi MI, Ozkaynakcıa A, Gorena MZ, Akera RG, Ozkarab C, and Onata FY (2011). The relationship between UGT1A4 polymorphism and serum concentration of lamotrigine in patients with epilepsy. Epilepsy Research. 2011; 95: 1–8.
6. De Cock RFW, Piana C, Krekels EHJ, Danhof M, Allegaert K, Knibbe CAJ The role of population PK-PD modelling in paediatric clinical research. European Journal of Clinical Pharmacology. 2011; 67(Suppl 1): S5-S16.

**Effects of a physical activity program on cognitive function, depression and quality of life in first-stage Alzheimer's patients: a randomized controlled trial**

Muammer Canbaz^1^ Neslihan Lok^2^*, Sefa Lok^3^

^1^ Department of Physical Education And Sports, Selçuklu Anatolian School, Konya, Turkey

^2^ Department of Psychiatric Nursing, Selçuk University Faculty of Health Sciences, Konya, Turkey

^3^ Department of Coaching, Selçuk University Faculty of Sport Sciences, Konya, Turkey

*Correspondence: Assist. Prof. Neslihan Lok, Department of Psychiatric Nursing, Selçuk University Faculty of Health Sciences, Konya, Turkey

**Keywords:** Alzheimer patients, Physical activity program, cognitive functions, depression, quality of life, randomized controlled trial

**Abstract**

**Purpose:** The aim of this study is to determine the effects of a physical activity program for first-stage Alzheimer's patients with regard to cognitive function, depression and quality of life.

**Materials and methods:** This is single-blinded randomized controlled trial. The experimental group included 75 Alzheimer's patients (40 in the experimental group, 35 in the control group), as diagnosed by physicians, who were in the moderate stage of the disease (SMMSE score: 23-24) and who were allowed to undertake physical activity. An information form including the participants' sociodemographic characteristics and medical history, the Standardized Mini Mental State Examination (SMMSE), the Quality of Life–Alzheimer’s Disease scale (QoL–AD) and Beck Depression scale were used for data collection. Statistical analysis was performed using SPSS 18.0 (IBM, Armonk, NY, USA). Continuous data were presented as medians (interquartile range), categorical data as counts and percentages. The researchers used descriptive statistics, including numbers, percentages, means and standard deviations.

**Results:** This study showed that the physical activity program improved cognitive function, depression and quality of life among first-stage Alzheimer's patients (*p*<0.05). These results suggest that regular physical activity programs should be used in routine practice to prevent cognitive decline and depression, and to the improve quality of life for first-stage Alzheimer's patients.

**Conclusion:** According to this study's findings, cognitive function, depression and quality of life among first-stage Alzheimer's patients improved as a result of a physical activity program.

**References**

1. Lam FM, Liao LR, Kwok TC, Pang MY. Effects of adding whole-body vibration to routine day activity program on physical functioning in elderly with mild or moderate dementia: a randomized controlled trial. International Journal of Geriatric Psychiatry. 2017:12:78-83.
2. Lok N, Lok S, Canbaz M (2017) The effect of physical activity on depressive symptoms and quality of life among elderly nursing home residents: randomized controlled trial. Archives of Gerontology and Geriatrics, 72;92-98.
3. Lök S, Lök N (2016) Kronik Psikiyatri Hastalarına Uygulanan Fiziksel Egsersiz Programlarının Etkinliği: Sistematik Derleme. Current Approaches in Psychiatry/Psikiyatride Guncel Yaklasimlar, 8(4).
4. Lok S, Lok N (2015) An Analysis of the Effects of the Physical Activities On the Cognitive Functions of the Old People with Mild Cognitive Impairment. European Psychiatry 30:1432.
5. Lök S, Lök N (2015) Demansta Fiziksel Aktivite ve Egzersiz. *Psikiyatride Güncel Yaklaşımlar*, *7*(3), 289-294.

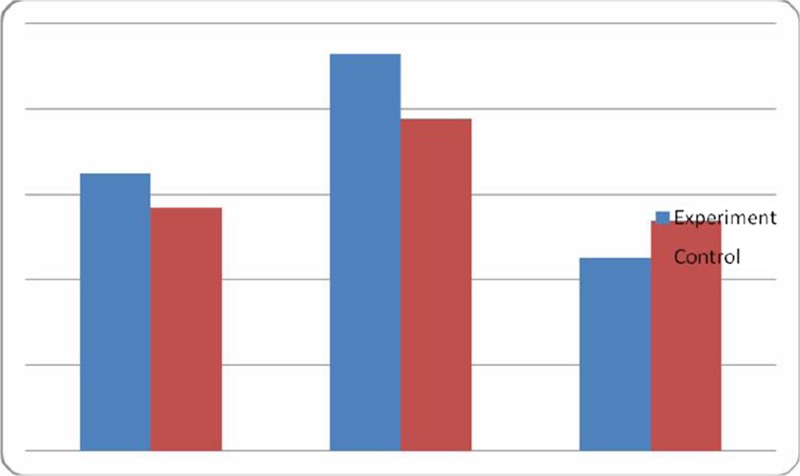


**Fig. 1** Distribution of cognitive functions, depression and quality of life scores among experimental and control groups after a physical activity program

**Systems medicine of mitochondrial parkinson’s disease**

Ronan M.T. Fleming ^1^

^1^Systems Biochemistry Group, Luxembourg Centre for Systems Biomedicine, University of Luxembourg

*Correspondence: Ronan MT Fleming, Systems Biochemistry Group, Luxembourg Centre for Systems Biomedicine, University of Luxembourg; e.mail: ronan.mt.fleming@gmail.com

**Keywords**: Systems medicine, Parkinson’s disease, iPSCs

**Abstract**

The overall objective of the Systems Medicine of Mitochondrial Parkinson’s Disease (SysMedPD, 2017-2021) project is to identify novel drug candidates capable of slowing the progression of neurodegeneration in the subset of Parkinson’s disease (PD) patients with overt mitochondrial dysfunction. Multi-modal phenotypic characterisation of cohorts of monogenic PD patients with overt mitochondrial dysfunction will be used as an anchor for the discovery of two extreme cohorts of idiopathic PD patients: with and without detectable mitochondrial dysfunction. A suite of personalised in vitro, in vivo, and in silico models will be generated using induced pluripotent stem cells (iPSCs) from selected subjects and controls. An industrial-quality 3D microfluidic cell culture product, specifically designed for the culture of iPSC-derived dopaminergic neurons, will be developed for use in a morphological and bioanalytical screen for lead compounds in order to reduce mitochondrial dysfunction. By monitoring motor behaviour and in situ striatal neurochemistry at high temporal resolution, the in vivo response to lead compounds will be characterised in humanised mouse models with striatal transplants of iPSC-derived dopaminergic neurons derived from PD patients. Personalised computational models of dopaminergic neuronal metabolism and mitochondrial morphology will be developed. These in silico models will be used to accelerate drug development by prioritising pathways for metabolomic assay optimisation, stratifying idiopathic PD patients by degree of mitochondrial dysfunction, predicting new targets for reducing mitochondrial dysfunction and mechanistic interpretation of in vitro and in vivo experimental results. SysMedPD unites a highly experienced multidisciplinary consortium in an ambitious project to develop and apply a systems biomedicine approach for preclinical identification of candidate neuroprotectants, for the estimated 1–2 million people worldwide who suffer from PD with mitochondrial dysfunction. An overview of the scientific rationale and main objectives of the project shall be presented, followed by preliminary results to date, particularly with respect to multi-scale mechanistic computational modelling of PD and the implications for systems medicine in other complex diseases.

**Flammer syndrome, a potential risk factor for central serous chorioretinopathy?**

Josifova Tatjana^1^, Katarzyna Koniezcka ^2^, Franz Fankhauser ^1^

^1^ Augenzentrum Prof. Fankhauser Bern, Switzerland

^2^ Universitätspital, Augenklinik Basel, Switzerland

*Correspondence: Prof. Dr. Med Josifova Tatjana, Augenzentrum Prof. Fankhauser Bern, Switzerland; e-mail: tatjana.josifova@augenzentrum-fankhauser.ch

**Keywords:** Flammer syndrome, Retinopathy, Predictive factors

**Abstract**

Central serous chorioretinopathy (CSCR) is the fourth most common retinopathy, typically affecting men in the third and fourth decades of life. The disease can be presented in acute or chronic form and can lead to decreased visual acuity (VA). Changes most often involve the macula, and are associated with pigment epithelial and neurosensory retinal detachment. Diverse risk factors such as the use of glucocorticoids, psychic stress, systemic hypertension, use of alcohol, and smoking have been reported in the pathophysiology of the disease. Several precise diagnostic tools showed that the choroidal blood flow is altered locally in the affected eye in CSCR. Our investigations of patients with CSCR showed that CSCR and Flammer syndrome have risk factors in common. Patients with Flammer syndrome generally have increased retinal vein pressure and elevated plasma endothelin-1 levels. This can be accompanied by choroidal hyperpermeability and subretinal fluid accumulation. Flammer Syndrome could be a potential risk factor for the development of CSCR. Screening for Flammer syndrome among CSCR patients can help us to better understand the pathophysiological process of CSCR development, and may lead to important discoveries regarding predictive factors for this disease.

**References**

1. Tittl MK, Spaide RF, Wong D, et al. Systemic findings associated with central serous chorioretinopathy. Am J Ophthalmol. 1999;128:63–68
2. Yoshioka H. The ethology of central serous chorioretinopathy. Arch. Ophth. 1991;95:1181–1195
3. Imamura Y, Fujiwara T, Margolis R, Spaide RF: Enhanced depth imaging optical coherence tomography of the choroid in central serous chorioretinopathy. Retina 2009;29:1469–1473
4. Yannuzzi LA. Central serous chorioretinopathy: a personal perspective. Am J Ophthalmol. 2010;149:361–363 5. Prünte C, Flammer J. Choroidal capillary and venous congestion in central serous chorioretinopathy. Am J Ophthalmol. 1996;12126- 34hy
5. Konieczka K, Ritch R, Traverso CE, Kim DM, Kook MS, Gallino A, Golubnitschaja O, Erb C, Reitsamer HA, Kida T, Kurysheva N, Yao K. Flammer syndrome. EPMA J 2014;5(1):11. doi:10.1186/1878-5085-5-11.

**Polymorphisms in the IL-7Ra gene associated with multiple sclerosis in a Slovak population**

Dusan Dobrota^1^, Daniel Čierny^1^, Jozef Michalik^2^, Jan Lehotsky^1^

^1^ Comenius University, Jessenius Faculty of Medicine, Martin, Slovakia

^2^ University Hospital in Martin, Clinic of Neurology, Martin, Slovakia

*Correspondence: Dr. Dusan Dobrota, e.mail: dobrota@jfmed.uniba.sk

**Abstract**

Interleukin-7 receptor-alpha (IL7-Ra) is involved in homeostasis of autoreactive T cells in multiple sclerosis (MS). Several studies have confirmed an association between the polymorphism rs6897932 C/T in the *IL-7Ra* gene and MS susceptibility. In our study, we tried to identify a possible association between this polymorphism and the risk of MS along with the rate of disease progression in a Slovak population. The rs6897932 polymorphism was detected in 219 clinically diagnosed MS patients and 218 healthy control subjects. We ascertained the Multiple Sclerosis Severity Score (MSSS) for each patient, and based on these scores, we chose 53 patients with rapidly progressing and 57 patients with slowly progressing disease. DNA samples were extracted from peripheral white blood cells and genotyped by PCR and restriction analysis. We found a significantly higher frequency of ancestral allele C in MS patients than in controls (*p* = 0.033). We found no significant differences in genotype or allele frequencies of SNP rs6897932 between the slow- and rapid-progression subgroups. Allele C of rs6897932 seems to increase the risk of MS development in a Slovak population. We did not confirm an association between this gene polymorphism and the rate of MS progression.

Acknowledgement: This work was supported by grant APVV 14-0088 and by project "CEVYPET" co-financed through EC sources and the European Regional Development Fund.

**Profiling the genetic variants responsible for hereditary ataxia in a Maltese family**

^1^Mark Briffa, ^1^Graziella Zahra, ^2^Joseph Borg.

^1^Molecular Diagnostics Laboratory, Department of Pathology, Mater Dei Hospital, Msida, MALTA

^2^Department of Applied Biomedical Science, Faculty of Health Science, University of Malta, Msida, MSD2080, MALTA

*Correspondence: Joseph Borg, Department of Applied Biomedical Science, Faculty of Health Sciences, University of Malta; e.mail: joseph.borg@biotech.um.edu.mt

**Keywords**: Hereditary ataxia, Next-generation sequencing, Bioinformatics

**Abstract**

Ataxia is a disorder which presents with varying degrees of loss of control of normal bodily movements. Although acquired ataxia may be encountered, it is more commonly found as a hereditary condition. A wide array of genetic variants have been identified as the cause of each of the multiple subtypes of inherited ataxia. Although several members of a Maltese family have been diagnosed with hereditary ataxia, little is known about the nature of the causative variant/s. The family history suggests an X-linked recessive pattern of inheritance. This study undertook the identification and profiling of genetic variant/s causing this disorder in the affected family members by means of next-generation sequencing using a clinical exome panel coupled with bioinformatics analysis. The data are presently being followed up by PCR amplification of the identified variant/s or gene/s and subsequent Sanger sequencing of the PCR product. Once established, efforts can be made to understand the mechanisms underlying the causative variant/s such that a cure, or more likely a symptomatic treatment, may be provided. Additionally, genetic counselling can be provided to guide future generations of this family line with regard to aspects of family planning.

**Altered sense regulation and related health conditions: is a predictive modelling possible?**

Nadja Koschelew^1^ and Olga Golubnitschaja*^2,3,4^

^1^CEMBIO, Rheinische Friedrich-Wilhelms-Universität Bonn, Germany

^2^Radiological Clinic, Rheinische Friedrich-Wilhelms-Universität Bonn, Sigmund-Freud-Str 25, 53105 Bonn, Germany

^3^Breast Cancer Research Centre, Rheinische Friedrich-Wilhelms-Universität Bonn, Bonn, Germany

^4^Centre for Integrated Oncology, Cologne-Bonn, Rheinische Friedrich-Wilhelms-Universität Bonn, Bonn, Germany

*Correspondence: Prof. Dr. Olga Golubnitschaja, Radiological Clinic, Rheinische Friedrich-Wilhelms-Universität Bonn, Sigmund-Freud-Str 25, 53105 Bonn, Germany; e.mail: Olga.Golubnitschaja@ukbonn.de

**Keywords**: Predictive preventive personalised medicine, Sense regulation, Diabetes, Flammer syndrome, Multi-level diagnostics, Biomarker panel

**Abstract**

Daily entities such as feeling of thirst, pain sensitivity and smell perception are generally conditioned by individual genetic set-up and post-genomic/epigenetic regulation – strongly influenced by social and environmental factors. On one hand, an abnormal (substantially deviating from the average) sense regulation is basically neglected at the standard level of medical care. On the other hand, an accumulated knowledge in the area of sense regulation demonstrates its relevance for development and progression of severe pathologies. For example, a significantly reduced feeling of thirst leading to low water intake results in body dehydration that is a well-acknowledged risk factor for breast cancer, amongst others [1]. Altered sense regulation attracts attention in both – diabetes mellitus, leading to cascaded comorbidities [2], and Flammer syndrome, suboptimal health conditions relevant for several pathologies investigated [3-5] (see Fig. 1).

*Thirsty feeling*

Reduced in Flammer syndrome with possible adverse health effects:

- short-term: dehydration, headache, migraine, mood disturbances, amongst others
- long-term: association with breast cancer and aggressive metastatic disease [1,5];

Increased in type 2 diabetes mellitus, possibly linked to fatigue, nausea, cancer, preeclampsia, hip fractures, nephropathy, coronary heart diseases, hypertension, venous thromboembolism, cerebral infarct

*Pain sensitivity*

Increased in Flammer syndrome, possibly linked to multiple sclerosis, fibromyalgia, impaired wound healing [4];

Decreased in type 2 diabetes mellitus and linked to hypertension, neuropathy, impaired wound healing, amongst others

*Smell perception*

Increased in Flammer syndrome [4]; consequences are currently unclear

Decreased in type 2 diabetes mellitus, possibly linked to cognitive impairments and neurodegenerative processes, also reduced in Alzheimer´s disease, Parkinson's disease and multiple sclerosis [2]

**References**

1. Golubnitschaja O. Feeling cold and other underestimated symptoms in breast cancer: anecdotes or individual profiles for advanced patient stratification? EPMA J 2017 8(1):17-22. doi:10.1007/s13167-017-0086-6
2. Doty R. L. Olfactory dysfunction in neurodegenerative diseases: Is there a common pathological substrate? The Lancet Neurology 2017;16(6):478–488. doi:10.1016/S1474-4422(17)30123-0.
3. Konieczka K., Ritch R., Traverso C. E., Kim D. M., Kook M. S., Gallino A., Golubnitschaja O., Erb C., Reitsamer H. A., Kida T., Kurysheva N. and Yao K. Flammer syndrome. EPMA J 2014 5(1):11. doi:10.1186/1878-5085-5-11.
4. Konieczka K., Koch S., Binggeli T., Schoetzau A., Kesselring J. Multiple sclerosis and primary vascular dysregulation (Flammer syndrome). EPMA J 2016 15 (7):13. doi:10.1186/s13167-016-0062-6.
5. Bubnov R., Polivka J. Jr., Zubor P., Koniczka K., Golubnitschaja O. Pre-metastatic niches" in breast cancer: are they created by or prior to the tumour onset? "Flammer Syndrome" relevance to address the question. EPMA J 2017 37 (10):12941. doi:10.1007/s13167-017-0092-8

**Fig. 1** Altered sense regulation in Flammer syndrome and Diabetes mellitus patients: opposed phenotypes are associated with completely different pathological conditions
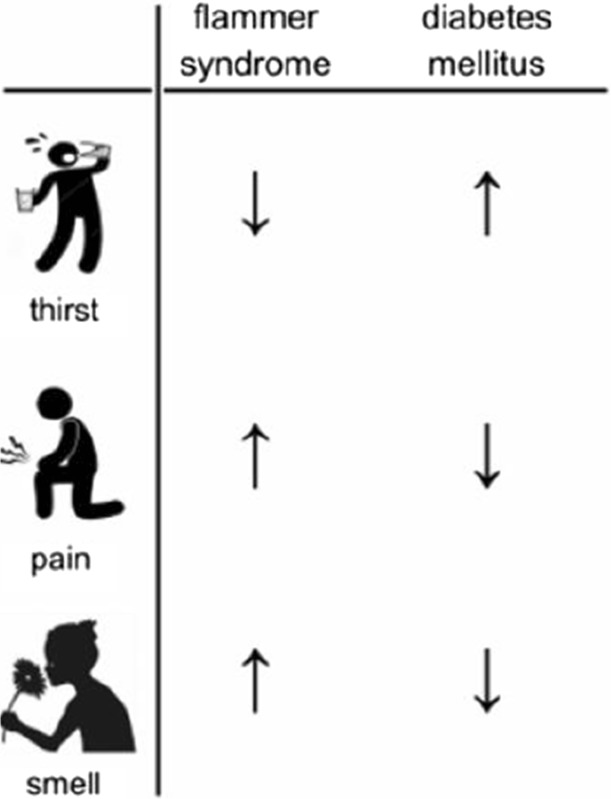


**PPPM STRATEGIES IN HEALTHCARE**

**Healthcare update from the “Golden State” (California): “Midas Medicine” marches to unpredictable but profitable and impersonal music**

Russell J Andrews^1,2^

^1^ World Federation of Neurosurgical Societies, Nyon, Switzerland

^2^ Nanotechnology & Smart Systems, NASA Ames Research Center, Moffett Field, CA, USA

*Correspondence: Russell J Andrews rja@russellljandrews.org

**Keywords:** Healthcare cost, Healthcare administration, Medical education, Universal healthcare

**Abstract**

The cost of healthcare in the United States (US) is 50–100% more than other developed countries. Indeed, one of its closest neighbors – Cuba – achieves an average lifespan equal to the US and lower infant mortality at one-tenth the cost.

California – nicknamed the “Golden State” for both its nineteenth- century gold mining and the color of its landscape – would be ranked roughly sixth in the world by GDP. California is frequently on the “cutting edge” of technology, climate change, and social policy. However, it is somewhat schizophrenic regarding healthcare.

In medical education, residents are increasingly burdened with ensuring maximize reimbursement for hospitals rather than learning their specific medical/surgical specialty and interacting with patients. Instruction on basic science and clinical medicine is being supplanted with instruction on enhancing the electronic medical record (e.g. maximizing the disease process “codes” for each patient) to ensure maximum reimbursement.

Another theme in both California and the US in general is the need for physicians to obtain advanced degrees such as the master of business administration (MBA) in order to participate in a healthcare organization. This highlights the difference between administering (i.e. enforcing a system’s rules) and managing (i.e. optimizing a system’s outcome), which is a fundamental difference between healthcare in the US and in the United Kingdom and elsewhere.

However, the California state legislature in 2017 has introduced the “Healthy California Act” – a proposal for universal single-payer healthcare coverage. In California it appears that legislators, no physicians, are playing “catch-up” with the rest of the developed world regarding healthcare!

**References**

1. Andrews RJ. Too Big to Succeed: Profiteering in American Medicine. iUniverse, Bloomington, IN, USA, 2013.
2. Martin WF, Long HW, Culbertson RA, Beyt E. The Master of Medical Management (MMM) degree: an analysis of alumni perceptions. J Health Admin Educ 24 (2007): 391-398.
3. Frenk J, Chen L, Bhutta ZA, et al. Health professionals for a new century: transforming education to strengthen health systems in an interdependent world. Lancet 376 (2010): 1923-1958.
4. Lara R, Atkins T. The Healthy California Act. California Senate Bill 562, introduced February 17, 2017, and amended March 29, 2017.

**Disaster response: global healthcare benefits of an integrated system**

Russell J Andrews^1,2^, Leonidas M Quintana^1,3^, Tariq M Khan^1,4^

^1^ World Federation of Neurosurgical Societies, Nyon, Switzerland

^2^ Nanotechnology & Smart Systems, NASA Ames Research Center, Moffett Field, CA, USA

^3^ Valparaiso University Medical School, Valparaiso, Chile

^4^ Northwest General Hospital and Medical School, Peshawar, Pakistan

*Correspondence: Russell J Andrews rja@russellljandrews.org

**Keywords:** Disaster response, Global surgery, Mobile surgical hospital, Telesurgery/telerobotics

**Abstract**

The United Nations (UN) estimates that disasters have cost over 1.3 million lives since 1995: following the 2010 Haiti earthquake, 20,000 people died each day from lack of basic surgery. To improve survival, disaster response (DR) must be on-site in 24 hours – not the current days/weeks of DR agencies (e.g., UN and Red Cross).

Trauma and stroke centers (TSCs) evolved based on evidence that "24/7" immediate treatment dramatically improves morbidity/mortality. Unlike DR, TSC equipment/personnel are seamlessly integrated into healthcare delivery/education systems. We propose that DR–like TSCs–be integrated into healthcare worldwide as disaster response centers (DRCs). A DRC includes a mobile operating room (including a car battery-powered CT, portable by helicopter) transported anywhere worldwide within hours. Telemedicine allows immediate subspecialty medical/surgical guidance; drones optimize utilization (e.g., identify the living buried in rubble, optimize resource triage).

Disasters evoke a humanitarian suspension of political, cultural and socioeconomic barriers that hinder response to other global crises. The DRC concept benefits from input/support by the World Federation of Neurosurgical Societies, the American College of Surgeons, UN disaster relief agencies, WHO emergency response, Aerospace Medical Association, Chilean Health Ministry, among others. Initial DRC sites are planned for Iquique (northern Chile) and Peshawar (northwest Pakistan).

This global "mega TSC system", with its multinational staff, will improve DR, establish global medical training standards, provide universal research platforms, and advance healthcare in developing countries. There are political, cultural and socioeconomic benefits – beyond the healthcare benefits – of integrating DR into the ongoing global healthcare system.

**References**

1. Andrews RJ, Quintana LM. Unpredictable, unpreventable and impersonal medicine: global disaster response in the 21st century. The EPMA Journal 2015; 6:1-12.
2. Meara JG, Leather AJM, Hagander L, et al. Global Surgery 2030: evidence and solutions for achieving health, wealth, and economic development. Lancet 2015; 386:569-624.
3. Murphy RR. Disaster Robotics. The MIT Press, 2014.
4. UNISDR + WMO: Disaster risk and resilience – thematic think piece. UN System Task Team on the Post-2015 UN Development Agenda, May, 2012. Available from: http://www.unisdr.org.
5. WHO Emergency Response Framework 2013: WHO performance standards. Available from: http://www.who.int.

**Cooperation: keynote for the future of health care**

Elisabeth van der Gulik

MaetisArdyn Arboservice, Utrecht, The Netherlands, retired

*Correspondance: Mrs. Elisabeth van der Gulik, MD, HonDL, Chirurgijn 21, 1188 DK Amstelveen, The Netherlands; e.mail: etm.vander.gulik@gmail.com

**Keywords:** Predictive preventive personalised medicine, International co-operation, Medical law, Multidisciplinary, Transparency, Quality codes, Continued education, Databases

**Abstract**

International co-operation has begun to guide the way that science and technical skills may contribute to a new global system of health care. Indeed, the trend in new research projects is one of working on a multidisciplinary basis. Physicians and scientists realize that collaboration offers a more effective approach to medical problems. Transparency in work and behaviour will bring colleagues and patients closer together. Quality and behaviour codes will be subject of special attention in the medical practice and of research as well. Medical law implementation and coaching will contribute to optimal medical working conditions. Education of health care workers will prepare them for their important roles, supported by continued education. People are aware of the need to care about the elements that the earth may be able to supply. Some antibiotics may become obsolete and will need to be replaced by other medicines. Databases must provide access to a patient’s medical history so that treatment can be individually tailored. Personalized, predictive, and preventive medicine is now a reality. Importantly, these efforts must all ultimately serve humankind through equal and honest distribution of health care to everyone.

**References**

1. Characiejus D, Hodzic J, Jacobs JJ. “First do no harm” and the importance of prediction in oncology, The EPMA J. 2010;1(3): 369-375. doi:10.1007/s13167-010-0042-1.
2. González, E.R., Mouttapa, M., Urban Revitlization and Health Justice: Questions and Recommendations, Californian Journal of Health Promotion 2013;11(2):iv-vii.
3. Golubnitschaja, O., Baban, B., Boniolo, G. et al. Medicine in the early twenty-first century: paradigm and anticipation - EPMA position paper 2016. EPMA J. 2016;7:23. doi:10.1186/s13167-016-0072-4.
4. Stewert, D.J., R. Kurzrock, Cancer:the Road to Amiens, J Clin Oncol. 2009;27(3):328-33. doi:10.1200/JCO.2008.
5. Wilhite, A.W., E.A. Fong, Coercive Citation in Academic Publishing, Science. 2012;335(6068):542-3. doi:10.1126/science.1212540.

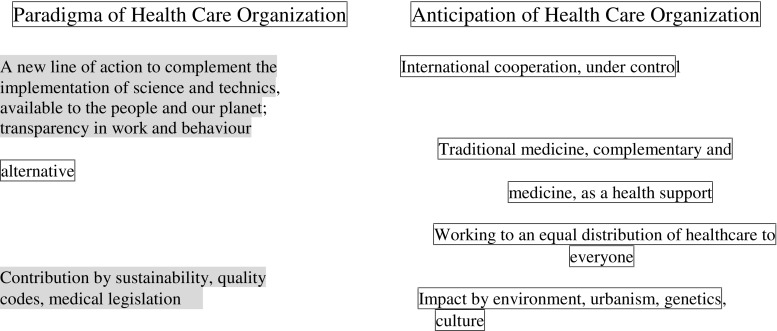


**Fig. 1** Future of health care, keynotes

**Innovative approaches to overcoming market access obstacles in predictive, preventive and personalized medicine (PPPM)**

Ildar Akhmetov^*,1^

^1^ Strategic Market Intelligence Department, Unicorn Consulting, Ukraine

*Correspondence: Ildar Akhmetov, Senior Market Access Analyst, Strategic Market Intelligence Dep., Unicorn Consulting, P.O.B. 91, Zhytomyr, 10020 Ukraine, email: ildar.export@gmail.com

**Keywords**: Predictive preventive personalized medicine, Market access, Healthcare economy, Stakeholder engagement, Value assessment, Risk-sharing schemes

**Abstract**

The continuously changing market access landscape affects prescription and reimbursement levels of medicines and diagnostics in PPPM. The preoccupation on the part of healthcare systems with developing robust cost-containment measures results in the emergence of new methods of value assessment, new forms of price setting, new customer structures, and changes in reimbursement and treatment guidelines. Although these changes facilitate access to existing medicines for certain patient groups, they create substantial barriers for new pharmaceutical, vaccine and diagnostic market entrants [2,4]. Thus, facing the lack of incentives in the early 2000s, several large biopharmaceutical companies abandoned antibiotics research and development (R&D) and investment, focusing instead on therapeutics in oncology and cardiology. As a consequence, resistance to existing medicines continued to increase, while the number of new antibiotics receiving marketing authorization declined [1].

This study *aims* to identify major patient access barriers in PPPM, and to understand how innovative companies overcome these market access restrictions. The presenter suggests that there exist a number of ways for producers to overcome market access barriers, for example, through early-phase convergence with key stakeholders, introduction of miscellaneous risk-sharing agreements, policy shaping, differentiating value-added services, and “tailoring” evidence generation to stakeholder requirements. [1,3,4].

**References**

1. Akhmetov, I. & Bubnov, R.V. Innovative Payer Engagement Strategies: Will the Convergence Lead to Better Value Creation in Personalized Medicine? EPMA J. 2017; 8 (17). doi:10.1007/s13167-017-0078-6
2. Akhmetov, I., Ramaswamy, R., Akhmetov, I., Thimmaraju, P.K. Market Access Advancements and Challenges in "Drug-Companion Diagnostic Test" Co-Development in Europe. J Pers Med. 2015; 5 (2). doi:10.3390/jpm5020213
3. Akhmetov, I. & Bubnov, R.V. Assessing Value of Innovative Molecular Diagnostic Tests in the Concept of Predictive, Preventive, and Personalized Medicine. EPMA J. 2015; 6 (19). doi:10.1186/s13167-015-0041-3
4. Moorkens, E., Jonker-Exler, C., Huys, I., Declerck, P., Simoens, S., Vulto, A.G. Overcoming Barriers to the Market Access of Biosimilars in the European Union: The Case of Biosimilar Monoclonal Antibodies. Front Pharmacol. 2016; 7 (193). doi:10.3389/fphar.2016.00193

**Christian Doppler – Laboratory for personalized medicine**

Kurt Krapfenbauer*^1^ and Olga Golubnitschaja^2,3,4^

^1^ Medical University Vienna, Austria

^2^Radiological Clinic, Rheinische Friedrich-Wilhelms-Universität Bonn, Germany

^3^Breast Cancer Research Centre, Rheinische Friedrich-Wilhelms-Universität Bonn, Germany

^4^Centre for Integrated Oncology, Cologne-Bonn, Rheinische Friedrich-Wilhelms-Universität Bonn, Germany

*Correspondence: Dr. Kurt Krapfenbauer, Medical University Vienna, Waehringer Guertel 18-20, 1090 Vienna, Austria; e.mail: Kurt_krapfenbauer@yahoo.de

**Keywords**: Predictive preventive personalised medicine, Diabetes, Innovative diagnostics technology, Biomarker panel

**Abstract**

**Innovative technology to be implemented:**

In personalized medicine, therapeutic intervention should be applied before the disease symptoms appear. This requires highly specific and highly sensitive biomarker arrays (BM) able to predict disease onset and therapeutic response [1]. Reliable, multiparametric semi-diagnostic and/or disease-predictive biomarker arrays are urgently needed for different diseases to improve early diagnosis or for monitoring and improving new and existing therapeutic strategies. The employment of biochip technologies for the simultaneous measurement of several biomarkers (multiplex assay) is necessary in biomedicine, since common assays (ELISA, Western blot, etc.) allow only the quantification of a single analyte at nano- to picogram levels, and are inadequate for quantifying low-abundance biomarkers in a complex matrix such as serum, urine or saliva [2].

**Aim of the institute:**

The proposed project, Christian Doppler – Laboratory for Personalized Medicine (CD-PM), is a multidisciplinary and collaborative effort aimed at performing innovative and novel research in the field of modern molecular diagnostics. The development and application of a novel, highly sensitive biochip will significantly enhance knowledge about biomarker detection in the early stage of diseases and thus their role in predictive medicine. The thinking behind this project includes both basic and oriented research, with a concrete commercial application.

**Application areas, examples:**

The proposed unique and novel, highly sensitive technique is currently being evaluated for the early detection of beta-cell dysfunction (diabetes mellitus) [3]. The CD-PM will validate other biomarker candidates already identified by key researchers from our cooperative partners.

**PPPM outlook:**

This novel technology has the capacity to significantly improve the sensitivity and specificity of existing analytical systems from femto to attogram levels. Once the assay platform is established, it can be retooled for nearly any diagnostic markers by replacing and optimizing the specific capture/detection conjugate, because the other components will be universal. Peripheral biomarkers for other metabolic disorders are currently under evaluation [4].

**References**

1. Golubnitschaja O, Baban B, Boniolo G, Wang W, Bubnov R, Kapalla M, Krapfenbauer K, Mozaffari MS, Costigliola V., Medicine in the early twenty-first century: paradigm and anticipation - EPMA position paper 2016. EPMA J. 2016;7:23. doi:10.1186/s13167-016-0072-4.
2. Drucker E, Krapfenbauer K., Pitfalls and limitations in translation from biomarker discovery to clinical utility in predictive and personalised medicine. EPMA J. 2013;4(1):7. doi:10.1186/1878-5085-4-7.
3. K. Krapfenbauer, Identification of beta cell dysfunction at the pre-symptomatic stage of diabetes mellitus by novel analytical system: liquid biopsy measurements in femtograms. EPMA J. 2017;8(1):35-41. doi:10.1007/s13167-017-0079-5.
4. Einhorn L, Krapfenbauer K. HTRF: a technology tailored for biomarker determination-novel analytical detection system suitable for detection of specific autoimmune antibodies as biomarkers in nanogram level in different body fluids. EPMA J. 2015;6:23. doi:10.1186/s13167-015-0046-y.

**The influence of environmental factors and stress on human health and chronic diseases: PPPM lessons from Antarctica**

Rostyslav V. Bubnov^1^*, Yevhen V. Moiseyenko^2,3^**, Mykola Ya Spivak^1^, NASC of Ukraine^2^

^1^ Zabolotny Institute of Microbiology and Virology, National Academy of Sciences of Ukraine, 154, Zabolotny Str., Kyiv 03680, Ukraine

^2^ National Antarctic Scientific Center of Ministry of Education of Ukraine, 16, Taras Shevchenko Boulevard, Kyiv 01601, Ukraine (http://www.uac.gov.ua/en/)

^3^ Bogomoletz Institute of Physiology, National Academy of Sciences of Ukraine, 4, Bogomoletz str., Kyiv 01024, Ukraine

*,**Correspondence: e-mail: rostbubnov@gmail.com **e-mail: moiseyenkoev@gmail.com

**Keywords:** Predictive, Preventive; Personalized medicine, Antarctica; Antarctic Akademik Vernadsky station, Microbiome, "Sterile" environment, Physiology research, Dysadaptation, Pain; Anxiety, Vascular dysregulation

**Abstract**

**Background.** Antarctica is a unique place to study health conditions under the influence of environmental factors on the organism in pure form [1]. Medical research in Antarctica opens up new horizons of understanding across a spectrum of individual health conditions under stress and unusual environmental conditions.

*Aim.* To analyze and discuss the retrospective medical data of long-term monitoring and observations at the Ukrainian Akademik Vernadsky Antarctica station and initial data from prospective studies.

**Methods.** We analyzed the multi-scale information of retrospective and prospective medical and biological studies, performed with the participation of all 22 Ukrainian wintering expeditions, based on surveys of over 220 men aged 20–60 years (mean age 37 years). Extensive medical examinations were carried out before the expedition, during the selection of candidates, and after returning, and particular functions were monitored during the entire stay in Antarctica. Medical records were analyzed to investigate microbiome, vascular dysfunction, hypoxia, mitochondrial dysfunction, and genetic polymorphism (hypoxia-inducible factor 1 (HIF-1) parameters.

**Results.** The examinations showed multi-level symptoms of the processes of dysregulation and dysadaptation, especially during adaptation to the Antarctic (1-month stay at the station) and, to a lesser extent, after returning from an expedition to Ukraine. A long stay in Antarctica evoked adaptive reactions associated with hypoxia and mitochondrial dysfunction, determined by a set of molecular-genetic mechanisms that trigger the expression of the corresponding genes and alter the mitochondria ultrastructure, leading to the death of organelles, and subsequently the cells, and are associated with pronounced oxidative stress [data in press].

Preliminary microbiome studies in the Antarctic setting have shown an increase in fungi on the skin of expedition participants, believed to be due to interference with local immunity and dysbiosis of the normal skin microbiome due to stress, recycled air, and antiseptic agents [1,2]. This region permits the study of conditions such as Flammer syndrome, amongst many others. Preliminary analysis demonstrated changes in responses on the Flammer syndrome questionnaire [3] carried out before and after the expedition; medical examinations showed stress dysadaptation via alteration the thyroid, gut function, and pain sensation [4].

**Conclusions.** The series of the retrospective and prospective studies demonstrated the strong influence of extreme environmental factors and stress in the absence of man-made factors on health parameters, with clearly defined individually patterned reactions.

The translation of obtained results enables the determination of the a “pre-pathology” condition for patient stratification that have a crucial impact on the management of chronic diseases in routine life by modifying stress-related conditions such as lifestyle, diet, and microbiome. In addition, since the region is essentially sterile, it permits unbiased research on the microbiome that may not be possible elsewhere.

**References**

1. Moiseyenko Y, Sukhorukov V, Pyshnov G, Mankovska I, Rozova K, Miroshnychenko O, Kovalevska O, Madjar S, Bubnov R, Gorbach A, Danylenko K, Moiseyenko O. Antarctica challenges the new horizons in predictive, preventive, personalized medicine: preliminary results and attractive hypothesis for multi-disciplinary prospective studies in the Ukrainian `Akademik Vernadsky` Station EPMA J. 2016; 7(1):11. doi:10.1186/s13167-016-0060-8
2. Reid G, Abrahamsson T, Bailey M, Bindels LB, Bubnov R, Ganguli K, Martoni C, O’Neill C, Savignac H, Stanton C, Ship N, Surette M, Tuohy K, van Hemert S. How do probiotics and prebiotics function at distant sites? Beneficial Microbes 2017, 8. In Press.
3. Bubnov R, Polivka J Jr, Zubor P, Koniczka K, Golubnitschaja O. “Pre-metastatic niches” in breast cancer: are they created by or prior to the tumour onset? “Flammer syndrome” relevance to address the question. EPMA J. 2017;8(2):141-157. doi:10.1007/s13167-017-0092-8.
4. Bubnov RV Evidence-based pain management: is the concept of integrative medicine applicable? EPMA J. 2012;3(1):13. doi:10.1186/1878-5085-3-13.

**Extension of laboratory medicine to provide complex health assessment in the context of PPPM**

Marko Kapalla^1,2^, Juraj Kubáň^1,2^

^1^ European Association for Predictive, Preventive and Personalised Medicine (EPMA), Brussels, Belgium

^2^ Negentropic Systems, s.r.o., Ružomberok, Slovak Republic

*Correspondence: Dr. Marko Kapalla, E-mail: marko.kapalla@gmail.com

**Keywords:** Predictive Preventive Personalised Medicine, Health policy, Laboratory medicine, Risk factors, Individualised patient profile, Vision

**Abstract**

Laboratory medicine is among the essential sources of reliable clinical information, providing the basis for objective disease/health assessment in individuals [1]. Predictive, preventive and personalized medicine (PPPM) emphasizes complex characteristics of health as a phenomenon [2]. In this context of complexity, laboratory medicine should not only significantly extend the range of tests and markers, but it should also significantly expand the range of distinct analyses, reflecting four principal categories of factors/materials related to health as outlined previously [3]. These principal categories comprise physical, chemical, biological and psychosocial factors, each of which can be of an internal and external nature. Complex clinical laboratory diagnostics/medicine should analyze factors/materials that have a potential impact on health and/or consistently reflect changes in health status. All data should then be interlinked with the patient’s standard medical record in order to prepare a complex report on his/her health/disease state in the context of PPPM (Fig. 1).

Such an extension of laboratory medicine, effectively supported by sophisticated information technology, will clearly represent a qualitative leap in healthcare and, in the long term, have the potential to significantly reduce healthcare costs through early prediction that is built on comprehensive, computer-assisted processing of reliable data acquired by complex analysis of various factors/materials, thus significantly reducing health deterioration in each individual.

The vision of EPMA is to build such a pilot complex clinical laboratory dedicated to the personal health assessment of apparently healthy individuals [4].

**References**

1. Golubnitschaja O, Watson ID, Topic E, Sandberg S, Ferrari M, Costigliola V. Position paper of the EPMA and EFLM: a global vision of the consolidated promotion of an integrative medical approach to advance health care. EPMA J. 2013;4(1):12. doi:10.1186/1878-5085-4-12.
2. Golubnitschaja O., Baban B., Boniolo G., Wang W., Bubnov R., Kapalla M., Krapfenbauer K., Mozaffari M.S. and Costigliola V.: Medicine in the early twenty-first century: paradigm and anticipation - EPMA position paper 2016, EPMA J. 2016;7:23. doi:10.1186/s13167-016-0072-4
3. Kapalla M, Kubáň J. How to estimate health-related costs: Economic aspect of healthy life style and its importance for PPPM, EPMA J. 2014; 5 (Suppl 1): A103. doi:10.1186/1878-5085-5-S1-A103
4. Kapalla M, Kubáň J, Costigliola V, Golubnitschaja O. Vision of the first EPMA center for predictive, preventive and personalized medicine in Europe, EPMA J. 2014; 5 (Suppl 1): A153. doi:10.1186/1878-5085-5-S1-A153

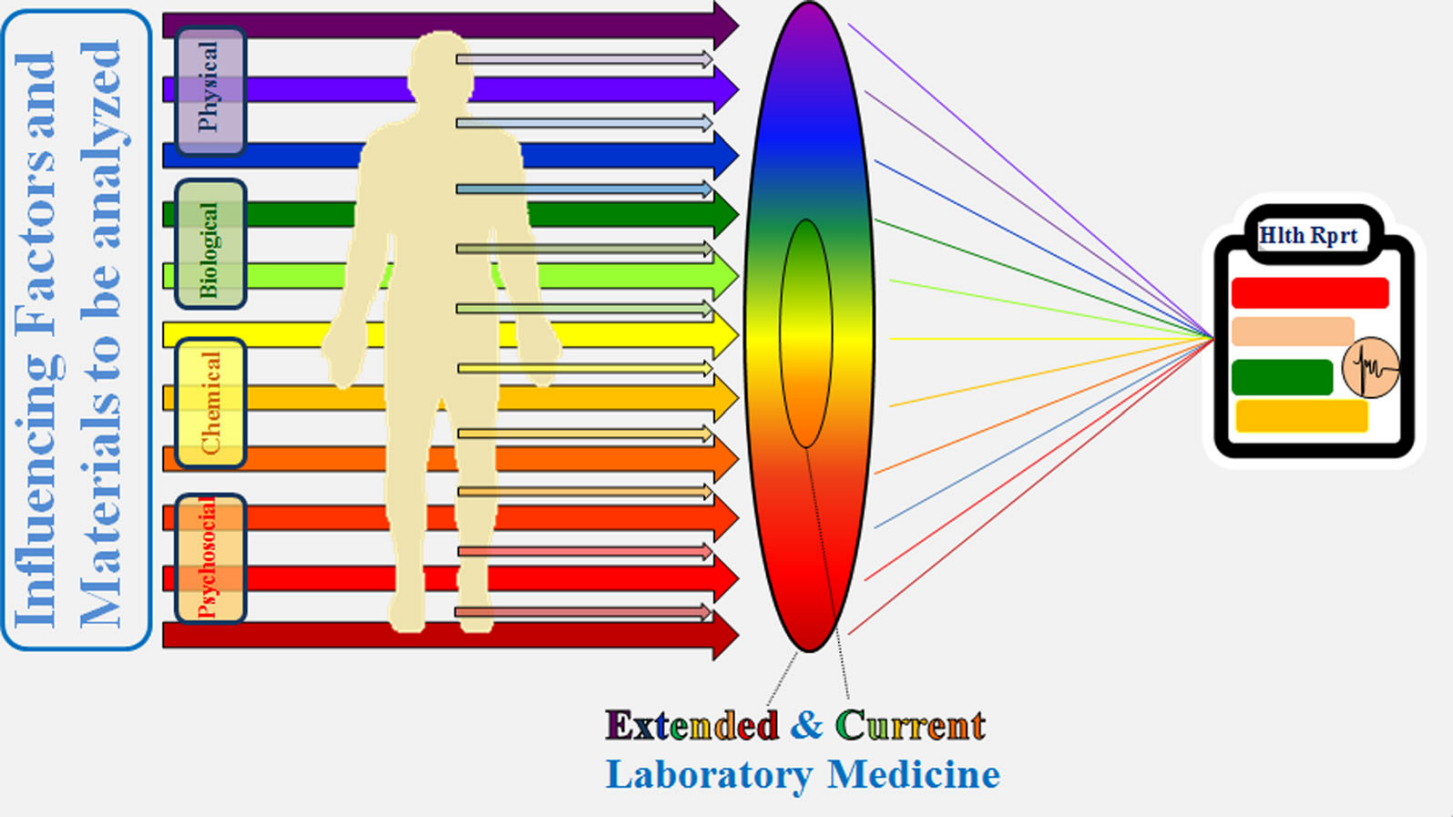


**Fig. 1** Four principal categories of external and internal factors and/or materials which extended laboratory medicine, in the context of PPPM, should take into consideration when assessing the health of an individual.

**Communication strategies for developing health awareness in preventive medicine**

Anna O. Stebletsova^1^, Anna V. Karpova^2^, Eugene V. Dorokhov^3^

^1^Head of Foreign Language Department,

^2^Dean of International Institute,

^3^Head Normal Physiology Department,

*Correspondence: PhD., Anna O. Stebletsova, Voronezh N.N. Burdenko State Medical University, Voronezh, Russia, Avenue of Revolution 14, 394036 Voronezh, Russia; e-mail: micvsma@yandex.ru

**Keywords:** Health awareness, Communication strategies, Preventive medicine

**Abstract**

Health awareness as a concept of health-related knowledge about disease prevention and treatment remains underutilized by Russian healthcare, whereas the English medical community demonstrates a persistent use of communication strategies for health education of the population. **The objective** was to identify communication strategies employed by Russian and English medical websites for health awareness development.

**Material and methods**. Ten official websites of English and Russian healthcare organizations and executive bodies were analyzed. Total corpus (*n*=78) included English (*n*=38) and Russian (*n*=40) texts. Contextual, descriptive and qualitative analysis was performed, which revealed 3three categories of strategies: informative, imperative, and mixed (informative + imperative).

**Results.** The English corpus (EC) demonstrated all strategies employed by the texts for the general public: informative (32%), imperative (25%), mixed (43%). The Russian corpus (RC) demonstrated a predominant use of informative strategies (57%) and significantly lower use of imperative (18%) and mixed (25%) ones.

**Conclusion.** The proportional involvement of all strategies in EC determines their effective contribution to the development of health awareness; the lower use of imperative and mixed strategies in RC may be an obstacle to developing health awareness in the population.

**A comprehensive and personalized imaging approach in emergency and limited-resource subsets**

Francesca M. Trovato, MD^1-2^

^1^University of Catania, Department of Clinical and Experimental Medicine, Catania, Italy

^2^Accident and Emergency Department, University Hospital “Policlinico-Vittorio Emanuele”, Catania, Italy

*Correspondence: Francesca M Trovato, MD; University of Catania, Department of Clinical and Experimental Medicine, via Santa Sofia 78, 95123, Catania, Italy; email:trovatofrancesca@gmail.com

**Keywords**: Ultrasound imaging, Emergency medicine, Limited resources, Predictive medicine

**Abstract**

Emergency and limited-resource subsets need sustainable, comprehensive and reliable diagnostic approaches [1-3]. Emergency physicians with imaging expertise can focus on further procedures, skipping time-consuming and expensive steps [4-5].

Emergency clinical ultrasound, as an adjunct to other reliable biomarkers, enables early predictive information, preventive management of impending or worsening conditions, and personalized therapy. Acute abdominal emergency (appendicitis, small bowel obstruction, gallstones, cholecystitis, aortic aneurysm), and particularly acute chest disease (pleural-pericardial effusion, pneumonitis, pleura-pulmonary consolidation), may present discordant information between ultrasound (US) and chest-X-ray (CXR). An ongoing study is addressing some new concepts in clinical and ultrasound stratification of respiratory disease in 73 consecutive patients referred to the same emergency facility. Key symptoms (dyspnea, chest pain, cough), laboratory assays (CRP, total leukocyte and neutrophilic counts) were considered, and the relative odds of finding concordant or discordant US and CXR imaging in pleural effusion and lung consolidation are displayed. In general, the predictive accuracy of combined conventional non-imaging clues (symptoms and laboratory assays) is insufficient for lung density and pleural effusion. Accuracy in the detection of pleural effusion is exceedingly higher for US (98.5 vs. 77.5), while the combined use of US and CXR, with one and/or both procedures positive, provides greater accuracy for lung density detection.

The implementation of a comprehensive culture of ultrasound imaging in medical school curricula and in continuous medical education is a sustainable advancement for the quality of our emergency departments and of the mobile medical units used in rescue activity during disaster or war crisis.

**References**

1. Interrigi MC, Trovato FM, Catalano D, Trovato GM. Emergency thoracic ultrasound and clinical risk management. Ther Clin Risk Manag. 2017 Feb 9;13:151-160. doi: 10.2147/TCRM.S126770
2. Sperandeo M, Rea G, Grimaldi MA, Trovato F, Dimitri LM, Carnevale V. Contrast-enhanced ultrasound does not discriminate between community acquired pneumonia and lung cancer. Thorax. 2017; 72(2):178-180. doi: 10.1136/thoraxjnl-2016-208913
3. Trovato FM, Catalano D, Trovato GM. Thoracic ultrasound: An adjunctive and valuable imaging tool in emergency, resource-limited settings and for a sustainable monitoring of patients. World J Radiol. 2016; 8(9):775-784.
4. Trovato FM, Catalano D. Diagnosis of Pneumonia by Lung Ultrasound in Children and Limited Resources Subsets: A Valuable Medical Breakthrough. Chest. 2016; 150(1):258-60. doi: 10.1016/j.chest.2016.04.032.
5. Sperandeo M, De Cata A, Molinaro F, Trovato FM, Catalano D, Simeone A,Varriale A, Martines GF, Trovato G. Ultrasound signs of pulmonary fibrosis in systemic sclerosis as timely indicators for chest computed tomography. Scand J Rheumatol. 2015; 44(5):389-98. doi: 10.3109/03009742.2015.1011228

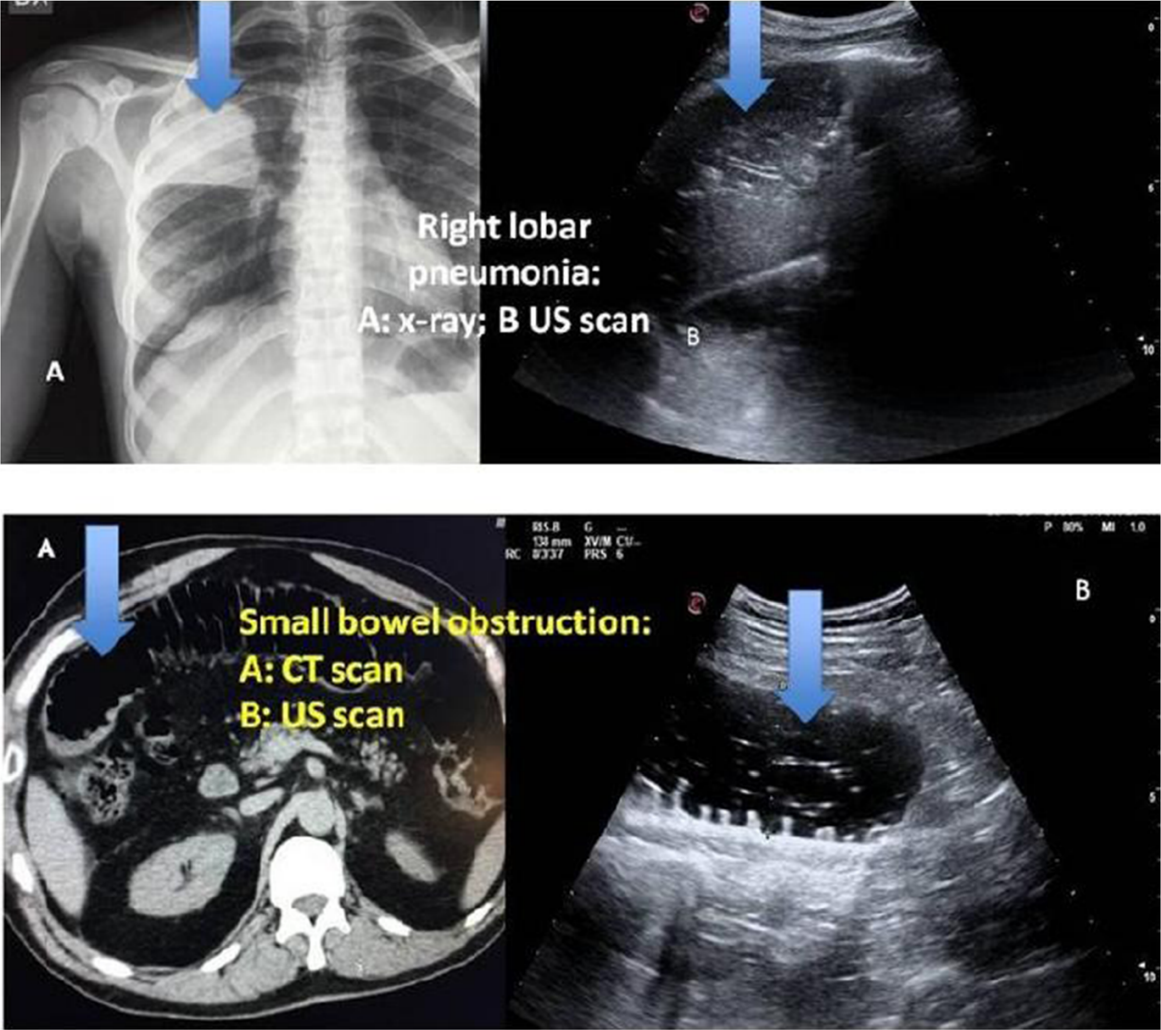


**Fig. 1** Top, right lobar pneumonia (A: x-ray; B: US scan). Bottom, small bowel obstruction (A: CT scan, B : US scan)

**The introduction of molecular blood transfusion in Malta**

^1^Giordmaina A, ^1^Debono J, ^2^Sutton G, ^2^Sant G, ^2^Spiteri R, ^2^Xuereb K, ^2^Niemistö E, ^3^Seria E, ^2^Borg J.

^1^Hospital Blood Bank, Mater Dei Hospital, Malta

^2^Department of Applied Biomedical Science, Faculty of Health Sciences, University of Malta, Msida, MSD2080 MALTA

^3^Centre for Molecular Medicine and BioBanking, University of Malta, Msida, MSD2080 MALTA

*Correspondence: Joseph Borg, Department of Applied Biomedical Science, Faculty of Health Sciences, University of Malta; e.mail: joseph.borg@biotech.um.edu.mt

**Keywords**: Blood group, Genotyping, Transfusion practice

**Abstract**

**Background.** The human blood group system is a complex protein-based system that consists of multiple antigens, including 54 antigens with more than 200 different alleles for RHD alone, and 50 different RHCE alleles. The five most important antigens from this blood group system are the D, C/c and E/e, since they are the cause of most alloimmunity.

**Aim**. Blood group genotyping is the method of choice when serological techniques cannot be used. In order to successfully use techniques such as PCR and DNA sequencing, one must first determine the frequency of said blood group antigen genotypes in the Maltese population. Thus far, the molecular transfusion group of Malta has investigated the Kidd, Cartwright, Colton and the major RHD and RHCE antigens.

**Materials and methods.** Four hundred blood donor samples were enrolled in this study. An allele-specific oligonucleotide polymerase chain reaction (ASO-PCR) was used to determine the presence of RhD, E and e, while multiplex PCR was used to test for RhCc, Cartwright and Colton genotypes. PCR RFLP was the method of choice for the Kidd genotypes.

**Results.** We present all current molecular data obtained thus far for the abovementioned blood group genotypes. In brief, out of 400 donor samples studied, the incidence of RhD was 90.75%, and 9.25% were negative for the D antigen. The incidence of the other Rh antigens was 76.75% for C and 73.75% for c, 25.25% for RhE and 97.75% for Rhe. The most common phenotype in RhD-positive samples was CcDee (36.50%), and in RhD negative it was ccdee (8%). The Colton Co(a+b-) exhibited the highest percentage occurrence (88.23%), whilst the heterozygous form of the phenotype, Co(a+b+) demonstrated a lower prevalence (5.88%). Co(a-b+) was not encountered in this study. Other data from different blood group antigens will also be presented.

**Conclusion**. These blood group systems have a vital role in population genetic studies and, most importantly, in resolving medical issues in transfusion practice, thus favouring the personalisation of health care in public health.

**Progress and challenges in controlling Kashin–Beck disease: epidemiological trends in China**

Kewei Wang^1, 2. 3^ and Dianjun Sun*^1, 2. 3^

^1^Center for Endemic Disease Control, Chinese Center for Disease Control and Prevention, Harbin Medical University, Harbin, China

^2^Key Laboratory of Etiology and Epidemiology (23618504), National Health and Family Planning Commission of the People's Republic of China, Harbin, China

^3^China and Russia Medical Research Center, Harbin, China

*Correspondence: Prof. Dr. Dianjun Sun, 157 Baojian Road, Harbin 150081, China; Email: hrbmusdj@163.com

**Keywords:** Kashin–Beck disease, Endemic disease, Incidence, Prevalence, National surveillance, Case–control study, Intervention trial, Multivariate linear regression

**Abstract**

Kashin–Beck disease (KBD) as an endemic disorder is clinically characterized by thickened and deformed joints. The etiology of KBD needs to be determined. The present report shows epidemiological characteristics of KBD via cross-sectional survey, national surveillance, case-control study, and intervention trial. X-ray images of the right hands of school children ages 7–12 were utilized to diagnose new KBD patients and disease severity. The national incidence of KBD was 22.1% in 1990, 16.0% in 1995, 12.3% in 2000, 5.5% in 2005, 0.38% in 2010, and 0.18 in 2015. In a series of valleys and villages of Tibet, Qinghai, Shaanxi, and Inner Mongolia, historical records show KBD prevalence of nearly 50%. In 2002, there were 819,666 individuals with KBD among 37,908,700 residents in endemic areas across China. By 2014, 611,187 patients out of 22,567,600 inhabitants were living in KBD-endemic areas. Typical features of KBD include early onset, gender equality, agricultural region, and uneven regional distribution. Epidemiological data show that KBD is associated with multiple risk factors such as fungal contamination of food grains, selenium deficiency, and food protein imbalance. Preventive measures include health funding in endemic areas, food diversification, food protein consumption, selenium dietary supplement, grain exchange, and relocation out of KBD areas. Comprehensive measures have been implemented in the prevention of KBD for a long period and so far a satisfactory result has been achieved. It is entirely possible to eradicate KBD from the earth.

**Preventive assessment of risk factors among students during their education in the university**

Maria Evsevyeva *^1^, Evgenij Shchetinin ^2^, Elena Italyantseva^1^, Anjelika Rusidi^1^

Stavropol State Medical University, Stavropol

^1^Student Health, Stavropol State Medical University, Russian Federation*

^2^Department of Pathological Physiology, Stavropol State Medical University, Russian Federation

*Correspondence: Prof. Dr. Maria Evsevyeva, Centre for Student Health, Stavropol State Medical University, Mira Str. 310, 355017, Stavropol, Russian Federation; e-mail: evsevieva@mail.ru

**Keywords**: Predictive preventive personalised medicine, Students, Risk factors, Hypertension, Pre-hypertension, Obesity, Smoking

**Abstract**

**Background.** Socially significant diseases have their roots in early years of life, as consolidation of negative behavioral stereotypes begins in youth [1].

**Objective.** To study the dynamics of the main risk factors (RF) in students over six years of their training at the medical university.

**Materials and methods.** Presented prospective analysis of a sample of 125 students enrolled in the first year in 2011, which was re-examined on the sixth course [2]. Fulfilled questionnaire survey aimed at identifying RF of type of arterial hypertension (AH)/pre-hypertensive (PH) [3], obesity, smoking, hypodynamia, and irrational nutrition [4].

**Results.** AH was found in 4.6% of freshmen, including 7.9% of boys and 3.4% of girls. It was recorded for 8.0% of graduates, with 13.2% of boys and 5.7% of girls. PH with blood pressure of 130/80 to 139/89 mm Hg was detected in 6.4% of school leavers, with 10.5% of boys and 4.6% of girls, and in 15.2% of graduates, including 23.6% of boys and 11.4% of girls. Cases of obesity and increased body mass index were identified in 9.0% during the first year of study, with 12.9% of boys and 7.7% of girls, and 22.2% in the sixth year, including 41.8% of boys and 15.5% of girls. The presence of physical inactivity was found in 14.8% of first-year students, including 9.6% of boys and 16.6% of girls, and id 38.8% of graduates, with 48.3% of boys and 35.5% of girls. Poor nutrition was present in 44.6% of first-year students, with 22.5% of boys and 52.2% of girls, and was found in 45.4% of graduates, including 48.3% of boys and 44.4% of girls.

**Conclusion.** The negative dynamics of behavioral and biological RF revealed among students during their study at university argues for early establishment of youth health examinations [5], which must include relevant preventive educational modules [4].

**References**

1. European Cardiovascular Disease Statistics. British Heart Foundation and European Heart Network 2005. http://www.herc.ox.ac.uk/research/cvd
2. Evsevyeva M.E., Eremin V.A., Eremin M.V., et al: Center for student's health: the main directions of work at the present stage. Preventive Medicine 2013, 16(1): 8-12.
3. Evsevyeva M.E., Mishchenko E.A., Rostovtsev M.V., Gal'kova I. Yu., et al: Circadian profile of arterial pressure at persons of young age with signs of prehypertension. Arterial hypertension. 2013. Vol. 19. No. 3. P. 263-269.
4. The ESC Textbook of Preventive Cardiology. Ed. by S. Gielen, G. De Backer, M. Piepoli, D. Wood, C. Jennings, I. Graham. Oxford University Press, 2016.
5. Di Chiara A and Vanuzzo D: Does surveillance impact on cardiovascular prevention? Eur Heart J 2009; 30: 1027-1029.

**Sleep quality and stress resistance among students related to additional work**

Maria Evsevyeva * ^1^, Vladimir Baturin^2^, Elena Mishchenko^1^, Victoria Jenenko^1^

^1^Centre for Student Health, Stavropol State Medical University, Russian Federation

^2^Research and Innovation Division, Stavropol State Medical University, Russian Federation

*Correspondence: Prof. Dr. Maria Evsevyeva, Centre for Student Health, Stavropol State Medical University, Mira Str. 310, 355017, Stavropol, Russian Federation; e-mail: evsevieva@mail.ru

**Keywords:** Students, Sleep quality, Stress resistance, Risk factors

**Abstract**

**Background.** Poor sleep contributes to the development of stress [1], which in turn is recognised as a risk factor for developing cardiovascular (CV) pathology [2,3].

**Objective.** Evaluation of sleep and anxiety/depressive disorders (ADD) of medical students based on their additional work in hospitals.

**Material and methods.** Forty-nine students of five or six courses of medical faculty of StGMU were surveyed with a questionnaire regarding subjective sleep characteristics (Russian Somnological Centre) using the Epworth daytime sleepiness scale, Stanford and Berlin questionnaires, and the Hospital Anxiety and Depression Scale (HADS), in order to determine the presence of ADD. Students were divided into two groups: working students (23) and non-working students (26). Statistical analysis was conducted using Microsoft Excel.

**Results.** Working students slept on average 6.0 ± 1,1 hours at night, and non-working students 7.06 ± 1.2 hours. Severe/moderate daytime drowsiness was found in 34% and 65% of students from the working group, but only 7% and 23% of the non-working group, respectively. Representation of apnea was similar between the two groups, but the structural characteristics of sleep quality differed significantly. The occurrence of pronounced/borderline values of ADD for the working students were 31% and 42%, and among the non-working group were 15% and 23%, respectively.

**Conclusion.** Additional work among medical students is associated with a decline in quality of sleep and development of ADD, which are in turn associated with reduced stress resistance. Working students must be included in the cardiovascular risk group [4] for timely implementation of preventive measures [5] as part of medical school student health centres. The number of such centres in Russia has been steadily increasing.

**References**

1. McEwen BS: Sleep deprivation as a neurobiologic and physiologic stressor: allostasis and allostatic load. Metabolism 2006, 55: S20-S23.
2. 2013 ESH/ESC Guidelines for the management of arterial hypertension: The Task Force for the management of arterial hypertension of the European Society of Hypertension (ESH) and of the European Society of Cardiology (ESC). Eur Heart J 2013, 34 (28): 2159-2219. doi:10.1093/eurheartj/eht151
3. Evsevyeva M.E. Stress-induced rearrangement of the myocardium: time course of structural changes in various types of stress. Bull Expl Biol Med 2000, 130(4): 937-939.
4. Hoevenaar-Blom MP, Spijkerman AM, Kromhout D, et al. Sleep duration and sleep quality in relation to 12-year cardiovascular disease incidence: MORGEN Study. Sleep 2011,34(11):1487-92.
5. Evsevyeva M. E., Eremin V.A., Eremin M.V., et al. Center for student's health: The main directions of work at the present stage. Preventive Medicine 2013, 16(1): 8-12.

**Improvement in preventive examinations among young people in the context of vascular age assessment**

Maria Evsevyeva* ^1^, Vladimir Koshel^2^ , Elena Fursova ^1^, Michail Eremin^3^ , Anjelika Rusidi^1^

^1^Centre for Student Health, Stavropol State Medical University, Russian Federation

^2^ Department of Otorhinolaryngology, Stavropol State Medical University, Russian Federation

^3^ Department of Otorhinolaryngology, Stavropol regional clinical hospital, Russian Federation

*Correspondence: Prof. Dr. Maria Evsevyeva, Centre for Student Health, Stavropol State Medical University, Mira Str. 310, 355017, Stavropol, Russian Federation; e-mail: evsevieva@mail.ru

**Keywords:** Predictive preventive personalised medicine, Young men, Risk factors, Vascular ageing, Cardio-ankle vascular index

**Abstract**

**Background.** Modern lifestyles promote the development of atherogenesis [1]. Therefore, it is advisable to intensify implementation of diagnostic screening for early vascular ageing (VA) among young men [2].

**Objective.** To identify cases of increased VA in younger men in relation to different risk factors (RF).

**Material and methods.** Fifty-five boys were examined at the Centre for Student Health of StSMU as part of the project "The University – Health Area". Analysis was performed on the occurrence of major RF and the state of the vascular wall in terms of the cardio-ankle vascular index (CAVI) and VA using the VaSera-1500 diagnostic software system Fukuda Denshi, Japan).

**Results.** The number of boys at the 95th percentile for R-CAVI and L-CAVI was 7.1 and 7.2, respectively. Of the 55 students, a CAVI level equal to or greater than it appeared was found for six people for R-CAVI (10.9%) and seven for CAVI-L 7 (12.7%). These young men differed in VA by 40–44 years. One or more RF were found in four of seven boys were distinguished. One student was characterised by dysfunction of connective tissue (DCT) [3], with the presence of corresponding phenotype, propensity for keloid scars, history of pneumothoraces, and mitral valve prolapse. Another young man suffered tonsillitis and pyelonephritis, and a third student had no RF.

**Conclusion.** The introduction of angiologic screening in the process of student examination can serve as a complement to traditional screening of groups with cardiovascular risk factors [4,5], as early VA can occur in the absence of other RF.

**References**

1. 2016 ESC Guidelines on cardiovascular disease prevention in clinical practice: The Sixth Joint Task Force of the European Society of Cardiology and Other Societies on Cardiovascular Disease Prevention in Clinical Practice (constituted by representatives of 10 societies and by invited experts). *Eur Heart J* Advance Access published June 8, 2016. doi:10.1093/eurheartj/ehw106
2. McGill H., McMahan C. Pathology of Atherosclerosis in Youth and the Cardiovascular Risk Factors. In: Pediatric Prevention of Atherosclerotic Cardiovascular Disease. Eds. R.M. Lauer, T.L. Burns, S.R. Daniels. Oxford, 2006: 3–26.
3. Eremin M.V., Evsevyeva M. E., Koshel V.I. Dysfunction of connective tissue and chronic tonsillitis. Monograph. Stavropol State Medical University. Stavropol, 2008 – 183p.
4. World Health Organization (WHO). Global strategy for prevention and control of non-communicable diseases Geneva 2014. http://www.health.gov.au/internet/main/publishing.nsf/Content/waon
5. Evsevyeva M.E., Eremin V.A., Eremin M.V., et al. Center for student's health: the main directions of work at the present stage. *Preventive Medicine* 2013, 16(1): 8-12.

**Prevalence and control of IDD in China**

Dianjun Sun*^1, 2. 3^ and Peng Liu^1, 2. 3^

^1^Center for Endemic Disease Control, Chinese Center for Disease Control and Prevention, Harbin Medical University, Harbin, China

^2^Key Laboratory of Etiology and Epidemiology(23618504), National Health and Family Planning Commission of the People's Republic of China, Harbin, China

^3^China and Russia Medical Research Center, Harbin, China

*Correspondence: Prof. Dr. Dianjun Sun, 157 Baojian Road, Harbin 150081, China; Email: hrbmusdj@163.com

**Keywords**: Iodine deficiency disorders (IDD), Universal salt iodisation (USI), Urinary iodine, Goiter rate, Cretinism, Surveillance, Information system

**Abstract**

Iodine deficiency disorders (IDD) have been recognised as a severe public health problem in China since the 1950s. Data recorded in the 1970s show 370 million people, and in 1993, 720 million people, at risk of IDD. Thirty out of 31 provinces and 1778 of 2835 counties were classified as endemic. In 1994, universal salt iodisation (USI) was launched to control IDD, and has achieved considerable success. As of 2005, China had eliminated IDD nationally [1], with children’s urinary iodine above adequate and goiter rate of 5%. However, since 2007, 342 new cases of cretinism have been found in Xinjiang and other provinces [2].

Over the last decade, through the implementation of measures of surveillance, investigation and evaluation, adjusting iodine content in salt and supplying iodised oil pills or free iodized salt, and the establishment of an IDD information system, China has maintained sustainable IDD elimination on a national basis [3]. The results of a national IDD survey in 2014 show a goiter rate of 2.6% among children’s, no deficient or excess urinary iodine was found among children in all provinces, and the coverage rate of qualified iodized salt had increased to 91.3%. No new cretinism cases have emerged since 2010.

China has built a solid base of working experiences over the 20 years of its USI programme, including a mechanism of “government leadership, sectoral collaboration and participation”, and a strategy of “locally appropriate and evidence-based iodine supplementation for various population groups”. Evaluations have shown enormous success achieved by China’s USI programme in meeting global and national IDD targets [4].

1. Su XH, Liu SJ, Shen HM, Zhang SB, Wei HL, Yu J, et al. National iodine deficiency disorder surveillance: a sum up of data in2005 and analysis. Chin J Endemiol. 2007; (26)1, 67-69. (In Chinese)
2. Li QL, Su XH, Yu J, Zhang SB, Liu P, Ji X, et al. Analysis of field survey results for iodine deficiency disorders in high-risk areas of China. Chin J Endemiol. 2009; 28(2),197-201. doi:10.3760/cma.j.issn.1000-4955.2009.02.023. (In Chinese)
3. Ministry of Health of the People's Republic of China, the National Standardization Management Committee. Criteria for elimination of iodine deficiency disorders. GB16006-2008. Beijing: Standards Press of China. (In Chinese)
4. Sun DJ. Review and recognition of key prevention and control issues of national key endemic diseases. Chin J Endemiol, 2014; 33(2), 121-124. doi:10.3760/cma.j.issn.2095-4255.2014.02.002 (In Chinese)

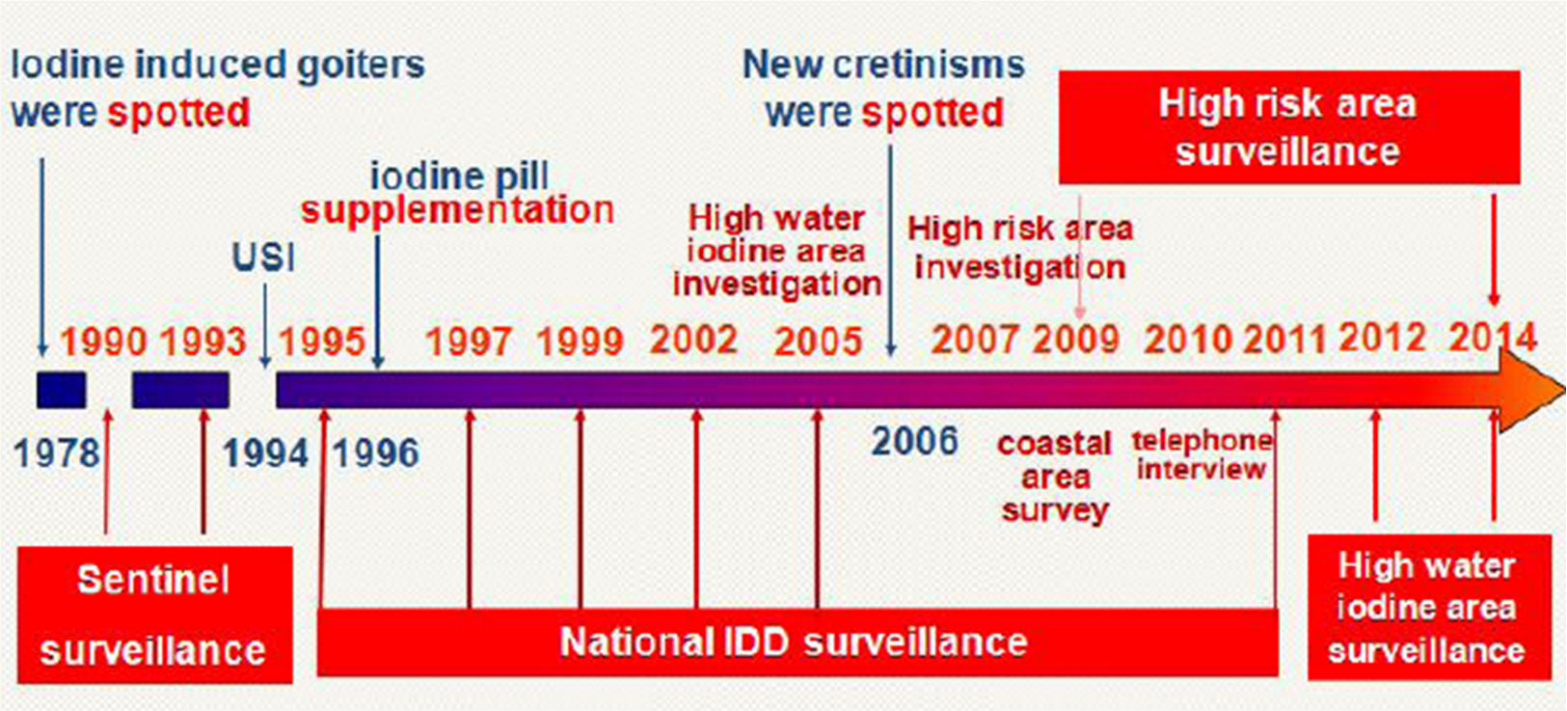


**Fig. 1** Surveillance and investigation of IDD in China

**Challenges of screening plant extracts for therapeutic development: lessons learned from a case in practice**

Frank Heemskerk

Research & Innovation Management Services bvba (RIMS), Bollestraat 75, 3090 Overijse, Belgium, e.mail: frank.heemskerk@telenet.be; T = +32 16 47 4092 (office), +32 47 999 4336 (mobile), skype <frankheemskerk>

**Abstract**

Natural extracts (plant metabolites, fungi, micro-organisms, etc.) are an extremely rich and diverse source of active therapeutic ingredients, or at the very least, contribute to preventive health and well-being. A majority of our current therapeutics are based on an original natural discovery or are still derived from natural sources. New medicine development is becoming prohibitively expensive, especially for chronic and rare diseases, and now, even for common viral and bacterial infections, (multi-)drug resistance is a widely occurring problem.

Global traditional medicine has maintained centuries of knowledge about effective healing with these extracts. Large collections of plants and extracts with metabolites have been well-characterized, often with traditional use, but beyond initial scientific interest, nothing is done with this knowledge base.

However, there are serious challenges to upscaling of production, including quality control concerns and inconsistent clinical evidence, posing difficulties in regulatory registration and obtaining market authorization. Lessons learned will be presented from an actual rich R&D program that we performed in a bio-pharmaceutical company to screen for and further clinically develop a therapy against leishmaniasis.

In fact, Research & Innovation Management Services (RIMS) is now one of three founding organizations of a new legal entity behind a European research infrastructure platform for natural extract screening with renowned institutes across Europe (www.euplantcropp.eu), for applications in medicine and nutraceuticals (protecting people) and agro-biotech (protecting plants). With this new structured approach, and with the most modern technologies through an European platform, an open discussion with EPMA members on potential targets (and indications for which diseases) would be valuable.

**Tackling socioeconomic disparities to revitalize the Mediterranean diet: a priority challenge at a time of economic crisis**

Licia Iacoviello*^1, 2^, Marialaura Bonaccio^1^, Giovanni de Gaetano^1^

^1^Department of Epidemiology and Prevention, IRCCS Istituto Neurologico Mediterraneo Neuromed, Pozzilli (Isernia), Italy

^2^Department of Medicine and Surgery. University of Insubria, Varese, Italy

*Correspondence: Prof. Licia Iacoviello, IRCCS Istituto Neurologico Mediterraneo Neuromed, via dell’Elettroncia, 86077 Pozzilli (IS), Italy; e-mail: licia.iacoviello@neuromed.it

**Keywords**: Mediterranean diet, Socioeconomic status, Health disparities, Economic crisis

**Abstract**

The Mediterranean diet has been associated with a reduced risk of developing major chronic diseases and with mortality [1]. Late evidence suggests, however, that people from Mediterranean areas are drifting away from this traditional diet, and has revealed emerging socioeconomic disparities in dietary habits even in epidemiological settings characterized by a relatively high socioeconomic homogeneity [2]. In this context, the economic crisis that started about 10 years ago poses a serious threat that the disparities in access to healthy foods will continue to grow. The decline in the Mediterranean diet began long ago, but a dramatic drop-off has been recorded in the most recent years. Data from the Moli-sani study showed that, beginning in 2007, material resources such as household income have become a strong determinant of the adherence to the Mediterranean diet, whereas they were not associated with diet in earlier years [3]. Although the shift was documented among the whole population, the decline is likely to be more evident across vulnerable groups and the elderly.

Indeed, evidence from a recent Italian national survey (INHES study) revealed that undesirable dietary modifications possibly linked to the current economic crisis were mainly reported by lower socioeconomic groups, and that subjects perceiving a negative impact of the recession on their diet also showed lower adherence to the Mediterranean diet and reduced quality of grocery products.

In light of this, a fruitful reasoning on the ways to revitalize the Mediterranean diet should necessarily deal with two key issues: First, the Mediterranean diet has become socioeconomically patterned, as had already been established for other quality eating models in non-Mediterranean settings, and the prominent role of financial over cultural resources in determining the adherence to this pattern should be taken into account. Second, the current economic crisis represents a major health threat for the general population, but in particular for the most vulnerable socioeconomic groups, possibly leading to wider gaps in terms of risk/protective factors across groups.

**References**

1. Sofi F, Abbate R, Gensini GF, Casini A. Accruing evidence on benefits of adherence to the Mediterranean diet on health: an updated systematic review and meta-analysis. Am J Clin Nutr. 2010;92(5), 1189-96. doi:10.3945/ajcn.2010.29673
2. Bonaccio M, Bonanni AE, Di Castelnuovo A, De Lucia F, Donati MB, de Gaetano G, et al. Low income is associated with poor adherence to a Mediterranean diet and a higher prevalence of obesity: cross-sectional results from the Moli-sani study. BMJ Open. 2012 19;2(6). doi:10.1136/bmjopen-2012-001685
3. Bonaccio M, Di Castelnuovo A, Bonanni A, Costanzo S, De Lucia F, Persichillo M, et al. Decline of the Mediterranean diet at a time of economic crisis. Results from the Moli-sani study. Nutr Metab Cardiovasc Dis. 2014;24(8), 853-60. doi:10.1016/j.numecd.2014.02.014

**Longevity – good luck or reasonable lifelong healthcare strategy?**

Ekaterina Silantyeva^1^ and Olga Golubnitschaja*^2,3,4^

^1^CEMBIO, Rheinische Friedrich-Wilhelms-Universität Bonn, Germany

^2^Radiological Clinic, Rheinische Friedrich-Wilhelms-Universität Bonn, Germany

^3^Breast Cancer Research Centre, Rheinische Friedrich-Wilhelms-Universität Bonn, Germany

^4^Centre for Integrated Oncology, Cologne-Bonn, Rheinische Friedrich-Wilhelms-Universität Bonn, Germany

*Correspondence: Prof. Dr. Olga Golubnitschaja, Radiological Clinic, Rheinische Friedrich-Wilhelms-Universität Bonn, Sigmund-Freud-Str 25, 53105 Bonn, Germany; e.mail: Olga.Golubnitschaja@ukbonn.de

**Keywords**: Predictive preventive personalised medicine, Longevity, Ageing, Multi-level diagnostics, Biomarker panel, laboratory medicine, Gerontology, Healthcare economy

**Abstract**

The mystery of longevity is attracting particular interest in the area of biomedical sciences. Why do some populations live longer and healthier than others (see Fig. 1)? Ageing as the antipode to longevity is known as the main risk factor for chronic/severe disorders such as impaired wound healing, cardiovascular disease, cognitive pathologies, cancer, type 2 diabetes mellitus and comorbidities with particularly poor outcomes (e.g. cancer patients with diabetic history [1]). Is it possible to slow ageing processes and consequently prevent the cascade of related pathologies? In order to reply to the question, it is crucial to understand which specific mechanisms are characteristic of longevity in contrast to accelerated ageing.

Accelerated ageing – definition

Rapidly decreasing capacity of the human body to effectively defend against internal and external damagers (extensive oxidative stress), leading to clinical onset of the age-related chronic pathologies

Longevity – definition

Stable capacity to effectively defend against damagers, remaining in excellent physical and mental health condition over plenty of life-decades

Potential biomarker panels for longevity include markers of lipidome (APOE, LPO, CETP), oxidation protection (FOXO3A, SOD, HSP70), genetic stability (*parp*, telomeres), and inflammation (IIS-system, DHEAS, SIRT1) [2].

Individualised patient profiles

*Longevity:* intact genetic information, mitochondrial stability, active reproductive function, efficient detoxification pathways, competent DNA-repair machinery, amongst others

*Accelerated ageing:* genomic instability, telomere attrition, mitochondrial dysfunction, cellular senescence, intercellular communication failure, impaired metabolic pathway [3]

**References**

1. Cebioglu M, Schild H, Golubnitschaja O. Cancer Predisposition in Diabetics: Risk Factors Considered for Predictive Diagnostics and Targeted Preventive Measures. EPMA J. 2010; 1 (1), 130-137. doi:10.1007/s13167-010-0015-4
2. Garatachea N, Emanuele E, Calero M, Fuku N, Arai Y, Abe Y, et al. ApoE Gene and Exceptional Longevity: Insights from Three Independent Cohorts. Exp Gerontol. 2014; 53, 16-23. doi:10.1016/j.exger.2014.02.004
3. López-Otín C, Blasco MA, Partridge L, Serrano M, Kroemer G. The Hallmarks of Aging. Cell. 2013; 153(6), 1194–217. doi:10.1016/j.cell.2013.05.039
4. Poulain M, Herm A, Pes G. The Blue Zones : Areas of Exceptional Longevity around the World. Vienna Yearbook of Population Research. 2013; 11, 87-108.
5. Kim IJ. Social Factors Associated with Centenarian Rate (CR) in 32 OECD Countries. BMC in Health Hum Rights. 2013; 13, 16. doi: 10.1186/1472-698X-13-16

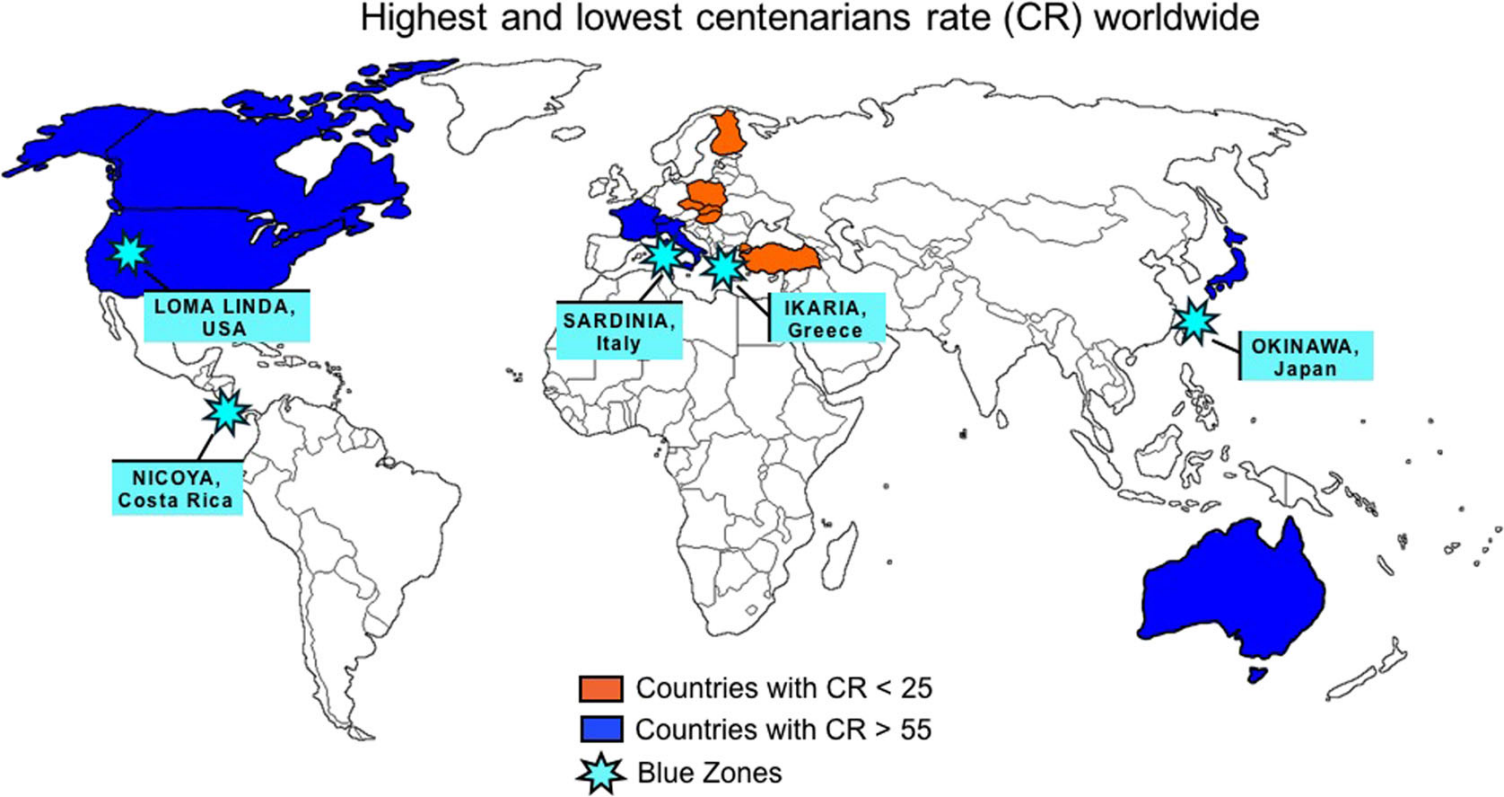


**Fig. 1** Centenarian/longevity map. Centenarian rate (CR) – number of people who survived to 100 years per 10000 persons alive at 60 years in the corresponding region 40 years ago; countries are divided into 2 groups: CR < 25 and CR > 55. Stars – 5 (blue zones) – areas with exceptionally high longevity, the populations of which share similar lifestyle and environmental factors [4, 5]

**PPPM IN DIABETES MELLITUS**

**The promise of whole body vibration for curtailing inflammation associated with obesity/type 2 diabetes: role of myeloid-to-lymphoid skewing**

Babak Baban, Jack Yu, Lin Yin, Aneeq Malik, Dorothea Rowe and Mahmood Mozaffari

Department of Oral Biology and Surgery,

Augusta University, Augusta, Georgia 30912-1128, USA.

*Correspondence: Babak Baban, Department of Oral Biology and Surgery, Augusta University, Augusta, Georgia 30912-1128, USA. bbaban@augusta.edu

**Keywords**: Diabetes, Type 2 diabetes, Myeloid, Lymphoid, Skewing, Inflammation

**Abstract**

The global epidemic of obesity and type 2 diabetes mellitus remains a pressing challenge for the scientific community and health care professionals (1). Chronic low-grade inflammation is believed to contribute significantly to the pathogenesis of target organ dysfunction/injury (e.g., nephropathy) in these conditions (1). Lifestyle interventions (e.g., diet and physical activity) are known to exert beneficial effects on glycemic control and general health in obese/type 2 diabetic individuals (2). Nonetheless, standard exercise programs are strenuous and time-consuming, and thus have low long-term compliance. In recent years, there has been a surge of interest in whole-body vibration (WBV) as a preventive and/or interventional modality that can be applied to populations at risk of obesity/type 2 diabetes and associated sequelae. Indeed, utilizing the db/db mouse model of obesity/type 2 diabetes, we recently showed beneficial effects of WBV on glycemic status in association with changes in several indices of inflammatory response, suggestive of a reduction in inflammation (3). Importantly, skewing toward myeloid cell production is often observed in chronic inflammation and autoimmune diseases (4). In this context, myeloid-to-lymphoid skewing is defined by an increased number of granulocyte/macrophage lineage, associated with a decrease in the frequency of cells with lymphoid lineage. Thus, our recent studies examined the impact of WBV in db/db mice and their db/m controls in the context of assessing changes in myeloid and lymphoid cells in the blood, bone marrow, and kidney. The results indicate increased myeloid potential associated with obesity/type 2 diabetes and a developmental skewing in favor of myeloid precursors in diabetic bone marrow. Importantly, WBV altered myeloid skewing in blood, kidney, and bone marrow of db/db mice suggestive of anti-inflammatory effects. Collectively, our observations are consistent with the immunomodulatory capability of WBV. Thus, WBV offers the promise of curtailing inflammation associated with obesity/type 2 diabetes, thereby positively influencing the outcome of these disorders.

**References**

1. Calle MC, Fernandez ML., Inflammation and type 2 diabetes., Diabetes Metab. 2012 Jun;38(3):183-91.
2. Merone L, McDermott R., Nutritional anti-inflammatories in the treatment and prevention of type 2 diabetes mellitus and the metabolic syndrome., Diabetes Res Clin Pract. 2017 May;127:238-253.
3. Yin H, Berdel HO, Moore D, Davis F, Liu J, Mozaffari M, Yu JC, Baban B., Whole body vibration therapy: a novel potential treatment for type 2 diabetes mellitus., Springerplus. 2015 Oct 6;4:578.
4. Oduro KA Jr1, Liu F, Tan Q, Kim CK, Lubman O, Fremont D, Mills JC, Choi K., Myeloid skewing in murine autoimmune arthritis occurs in hematopoietic stem and primitive progenitor cells., Blood. 2012 Sep 13;120(11):2203-13.

**A multilevel integrative approach for the prediction of the development of metabolic syndrome**

Estelle Pujos-Guillot^1,2^, Julien Bertrand^1^, Mathieu Rambeau^1^, Mélanie Pétéra^2^, Marion Brandolini^2^, Antony Fernandes^2^, Joanne Matta^4^, Claire Lévy-Marchal^3^, Sébastien Czernichow^4,5^, Blandine Comte^*1^

^1^INRA, UMR1019, Mapping, Clermont-Ferrand, France;

^2^INRA, UMR1019, PFEM, Clermont-Ferrand, France;

^3^INSERM, Institute of Public Health, Paris, France;

^4^UMS11 Cohortes en population, INSERM/Université Versailles Saint-Quentin, Villejuif, France;

^5^Department of Nutrition, Ambroise Paré University Hospital, Boulogne-Billancourt, France

*Correspondence: Dr Blandine Comte, INRA Research Centre of Clermont-Ferrand/Theix, F-63122 St Genès-Champanelle, France; e.mail: blandine.comte@inra.fr

**Keywords**: Multilevel prediction, Metabolic syndrome, Metabolomics, Proteomics, Multidimensional biomarkers, Patient stratification

**Abstract**

The rising prevalence of metabolic syndrome (MetS), a cluster of cardiometabolic risk factors predictive of type 2 diabetes, relates largely to increasing obesity and sedentary lifestyle, but also to early metabolic life events. The objective of the study was to build predictive models of evolution toward MetS 8 years later, and to bring new knowledge about this pathological state using a multilevel approach in an at-risk population (subjects with low birth weight). A case–control study (subjects free from MetS at baseline (*n*=92 born small vs. *n*=76 born adequate for gestational age) was designed within the French community-based Haguenau cohort. Serum signatures were determined and compared at baseline (age 20 years) to determine predictive biomarkers using both un-targeted metabolomics and targeted proteomics. Individual predictive models were first built using linear logistic regressions from the omics datasets and epidemiological data. All data were then integrated to determine whether multidimensional models improved prediction. Univariate statistical analyses enabled the identification of 93 discriminant metabolites and 37 proteins between cases and controls at baseline, with gender differences. The resulting models based on either four metabolites or four proteins showed good performance: 18% misclassification (19% cross-validation error rate) vs. 11% misclassification (19% cross-validation), respectively. Multi-omic data integration improved the performance and robustness of the prediction (9% misclassification, 16% cross-validation). Correlation analyses also contributed to a better understanding of the role of these biomarkers in the pathological processes. These results should provide new tools for better stratification of at-risk populations. This project was supported by the Fondation Francophone de Recherche sur le Diabète.

**Input of multidimensional phenotyping in the metabolic syndrome stratification**

Estelle Pujos-Guillot^*1,2^, Stéphanie Monnerie^1^, Etienne Thévenot^3^, Christophe Junot^4,^ José Morais^5^, Hélène Payette^6^, Pierrette Gaudreau^7^, Blandine Comte^2^

^1^INRA, UMR1019, Mapping, Clermont-Ferrand, France

^2^INRA, UMR1019, PFEM, Clermont-Ferrand, France

^3^Plateforme MetabolomeIDF, CEA, LIST, Saclay, DRT/LIST Laboratory for Data Analysis and Systems’ Intelligence, Gif sur Yvette, France

^4^Plateforme MetabolomeIDF, CEA Saclay, Laboratoire d'Etude du Métabolisme des Médicaments, DRF / Institut Joliot / SPI, Gif sur Yvette, France

^5^Division of Geriatric Medicine McGill University, Montreal, Canada

^6^Faculté de médecine et des sciences de la santé Université de Sherbrooke, Sherbrooke, Canada ; Centre de recherche sur le vieillissement Centre intégré universitaire de santé et de services sociaux de Sherbrooke, Sherbrooke, Canada

^7^Centre de Recherche du Centre hospitalier de l’Université de Montréal, Montreal, Canada ; Département de médecine, Université de Montréal, Montreal, Canada

*Correspondence: Dr Estelle Pujos-Guillot, INRA Research Centre of Clermont-Ferrand/Theix, F-63122 St Genès-Champanelle, France; e.mail: estelle.pujos-guillot@inra.fr

**Keywords**: Personalized medicine/nutrition, Multidimensional phenotyping, Data integration, Metabolic syndrome, Patient stratification

**Abstract**

Metabolic syndrome (MetS) is defined by a cluster of cardiometabolic factors including obesity, hypertension, dysglycemia, and dyslipidemia. It affects a growing number of persons, in particular older adults often suffering from multiple chronic diseases, and its prevalence is now a public health challenge. In the context of personalized medicine/nutrition, new tools are necessary to bring additional knowledge about MetS etiology, better stratify populations, and customize strategies for prevention.

A nested case–control study on MetS was designed within the Quebec Longitudinal Study on Nutrition and Successful Aging (NuAge). It includes 61 cases and 62 controls of similar age (68–82 years), selected among the 853 men. Both targeted and un-targeted metabolomic/lipidomic approaches, available within the MetaboHUB French infrastructure [1], will be performed on serum samples collected at recruitment during 2003–2005 (T1) and 3 years later (T4). Data analysis will be performed using reproducible online Galaxy workflows [2].

The metabolomic/lipidomic data will be processed to identify specific signatures of MetS and its components, and to study their stability over time. These data will then be analysed for evaluation of a molecular reclassification of the MetS phenotype. Finally, they will be integrated with available phenotypic and detailed nutritional data in order to better characterize sub-phenotypes (Fig. 1).

The approach developed here will open a door for a more comprehensive understanding of the metabolic phenotype resulting from the complex interplay between intrinsic and extrinsic factors. Thus, this project will allow an improved description of MetS-associated characteristics and will offer new tools for better patient stratification in elderly populations.

**References**

1. http://www.metabohub.fr
2. http://workflow4metabolomics.org

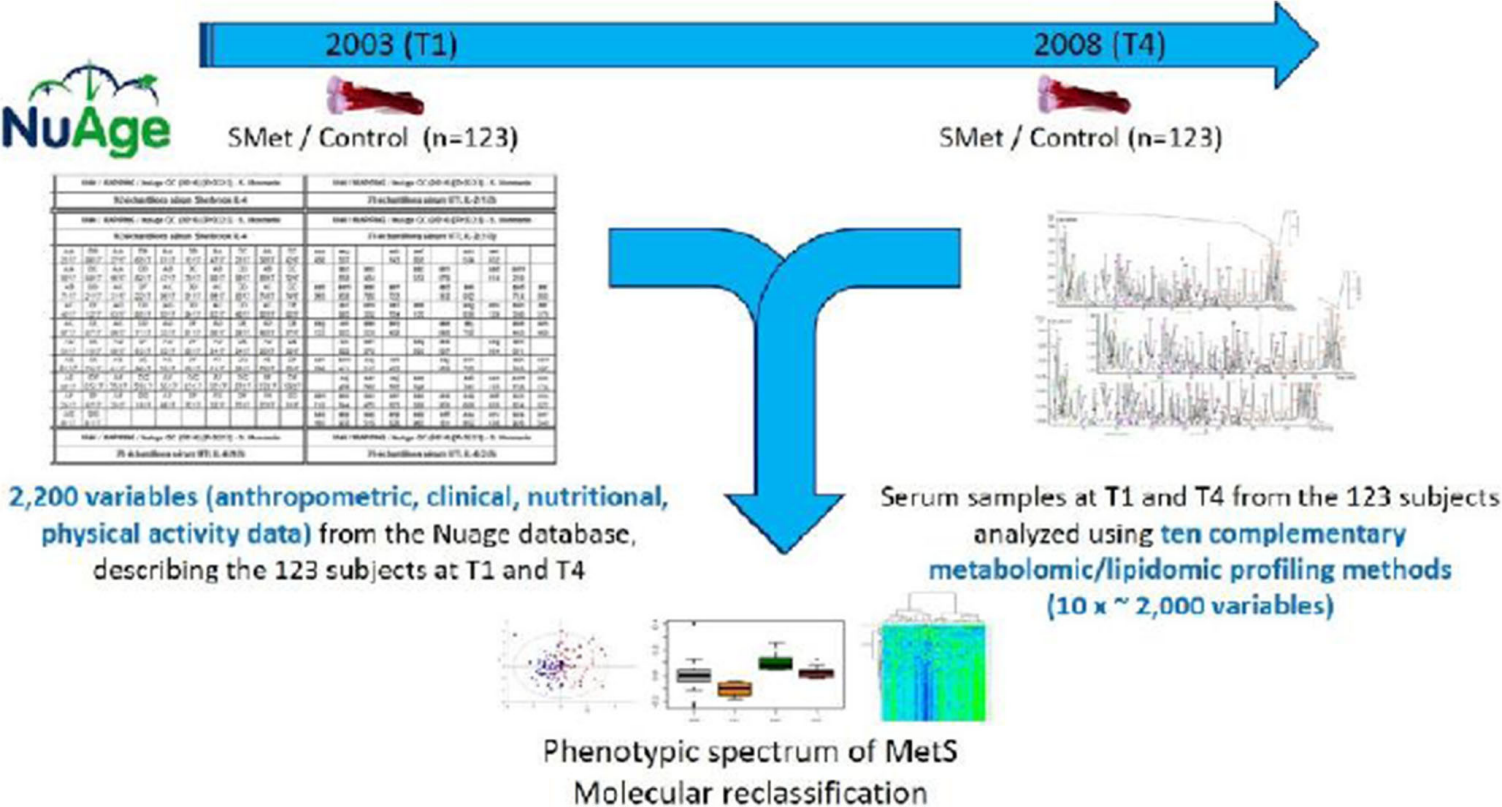


**Fig. 1** Description of the overall study strategy.

**Predicting cardiovascular risk in diabetic patients: are we all on the same side?**

Ivica Smokovski*^1,2^ and Tatjana Milenkovic^1,3^

^1^ University Clinic of Endocrinology, Diabetes and Metabolic Disroders, Skopje, Macedonia

^2^ Faculty of Medical Sciences, University Goce Delcev, Stip, Macedonia

^3^ Medical Faculty, University Sts Ciryllus and Methodius, Skopje, Macedonia

*Correspondence: Assoc Prof Ivica Smokovski, MD, PhD, University Clinic of Endocrinology, Diabetes and Metabolic Disorders, Vodnjanska 17, MK-1000 Skopje, Macedonia; e.mail: ivica.smokovski@ugd.edu.mk

**Keywords:** Diabetes, Cardiovascular disease, Predictive models, Cardiovascular risk, IDF, ESC, EASD, ACC, AHA, ADA, NICE

**Abstract**

Cardiovascular diseases are the main reason for morbidity and mortality in diabetic patients, and cardiovascular risk is increased at least twofold in men and at least fourfold in women with diabetes compared to non-diabetic populations. Predictive medicine is of the utmost importance in the clinical care of diabetic patients, since predicting cardiovascular risk is essential for modification of risk factors aimed at prevention or delay of future cardiovascular events. The prediction of cardiovascular risk is a valuable tool within the context of patient-centered care, as it includes active participation of diabetic patients in the decision-making process, resulting in higher compliance with the treatments agreed. However, there are differences among the current guidelines of various international authorities, such as the International Diabetes Federation (IDF), European Society of Cardiology (ESC) / European Association for Study of Diabetes (EASD), American College of Cardiology (ACC) / American Heart Association (AHA), American Diabetes Association (ADA), and National Institute for Health and Care Excellence (NICE), for the prediction of cardiovascular risk in diabetic patients. Furthermore, the clinical use of models with classic risk factors and novel biomarkers that would predict cardiovascular risk in diabetic patients from various populations with acceptable precision poses a challenge. Taking into consideration the global diabetes pandemic and its close association with cardiovascular diseases, there is an urgent need for streamlining of current guidelines on the prediction of cardiovascular risk and its use in clinical practice.

**References**

1. International Diabetes Federation, Clinical Guidelines Task Force, Global Guideline for Type 2 Diabetes, 2012.
2. The Sixth Joint Task Force of the European Society of Cardiology and Other Societies on Cardiovascular Disease Prevention in Clinical Practice. 2016 European Guidelines on cardiovascular disease prevention in clinical practice. European Heart Journal, 2016; 37:2315–2381.
3. Goff DC Jr, et al. 2013 ACC/AHA Cardiovascular Risk Guideline, 2013 ACC / AHA Guideline on the Assessment of Cardiovascular Risk A Report of the American College of Cardiology / American Heart Association Task Force on Practice Guidelines. Circulation, 2013; doi: 10.1161/01.cir.0000437741.48606.98.
4. American Diabetes Association, Standards of Medical Care in Diabetes 2017 - Cardiovascular Disease and Risk Management. Diabetes Care 2017; 40(Suppl. 1):S75–S87.
5. National Institute for Health and Care Excellence. Cardiovascular disease: risk assessment and reduction, including lipid modification. Available at: nice.org.uk/guidance/cg181. Accessed 20-May-2017.

**Insulin resistance and its concomitant effect on vitamin D status among type 2 diabetics**

Samuel Asamoah Sakyi^1^*, Linda Ahenkorah Fondjo^1^, William K. B. A. Owiredu^1^, Richard Ephraim Dadzie^3^, Edwin Ferguson Laing^1^, Michael Acquaye Adotey-Kwofie^2^,

^**1**^ Department of Molecular Medicine, School of Medical Sciences, College of Health Sciences, Kwame Nkrumah University of Science and Technology, Kumasi, Ghana, ^**2**^ Nkawie Government Hospital, Kumasi, Ghana. Department of Medical Laboratory Technology, University of Cape Coast, Ghana.

*Correspondence: Dr Samuel Asamoah Sakyi, Dept. of Molecular Medicine, SMS-KNUST, Kumasi, Ghana sasakyi.chs@knust.edu.gh

**Keywords:** Insulin resistance, Type 2 diabetes, Vitamin D, Glucose homeostasis

**Abstract**

Vitamin D plays a pivotal role in functional processes that control immune function and mineral metabolism, as well as several chronic conditions [1, 2]. Emerging data indicate a possible influence of vitamin D on glucose homeostasis [3, 4, 5]. This study sought to provide initial information on vitamin D status among a Ghanaian population with type 2 diabetes and its association with glucose homeostasis. This case–control study was conducted among 118 patients with clinically diagnosed type 2 diabetes mellitus (T2DM) attending diabetic clinic at the Nkawie Government Hospital between October and December 2015. One hundred healthy non-diabetics living in Nkawie district were selected as controls. Questionnaires were administered to obtain sociodemographic data. Venous blood samples were taken from both cases and controls to estimate their fasting blood glucose (FBG) and lipid profile by spectrophotometry and iPTH and 25(OH)D by enzyme-linked immunosorbent assay (ELISA). Statistical analyses were performed using SPSS 20.0 software. The average age of the study participants was 58.81 years for cases and 57.79 year for controls. Vitamin D deficiency was found in 92.4% of T2DM cases and 60.2% of the non-diabetic controls. Vitamin D deficiency was not significantly associated with HOMA-**β** [T2DM: *r* ^2^ = 0.0209, *p* = 0.1338 and Control: *r* ^2^ = 0.0213, *p* = 0.2703] and HOMA-IR [T2DM: *r* ^2^ = 0.0233, *p* = 0.1132 and Control: *r* ^2^ = 0.0214, *p* = 0.2690] in either the controls or cases. These results show that vitamin D deficiency was prevalent in both those with and without T2DM. There was no association between vitamin D deficiency and insulin resistance among our study population. However, vitamin D supplementation in T2DM patients is recommended.

1. Holick MF. Vitamin D deficiency. N Engl J Med. 2007;357(3):266-81.
2. Holick MF, Chen TC. Vitamin D deficiency: a worldwide problem with health consequences. Am J Clin Nutr. 2008;87(4):1080S-6S.
3. Anderson JL, May HT, Horne BD, Bair TL, Hall NL, Carlquist JF, et al. Relation of vitamin D deficiency to cardiovascular risk factors, disease status, and incident events in a general healthcare population. Am J Cardiol. 2010;106(7):963-8. doi:10.1016/j.amjcard.2010.05.027.
4. Pittas AG, Harris SS, Stark PC, Dawson-Hughes B. The effects of calcium and vitamin D supplementation on blood glucose and markers of inflammation in nondiabetic adults. Diabetes Care. 2007;30(4):980-6.
5. Talaei A, Mohamadi M, Adgi Z. The effect of vitamin D on insulin resistance in patients with type 2 diabetes. Diabetol Metab Syndr. 2013;5(1):8. doi:10.1186/1758-5996-5-8.

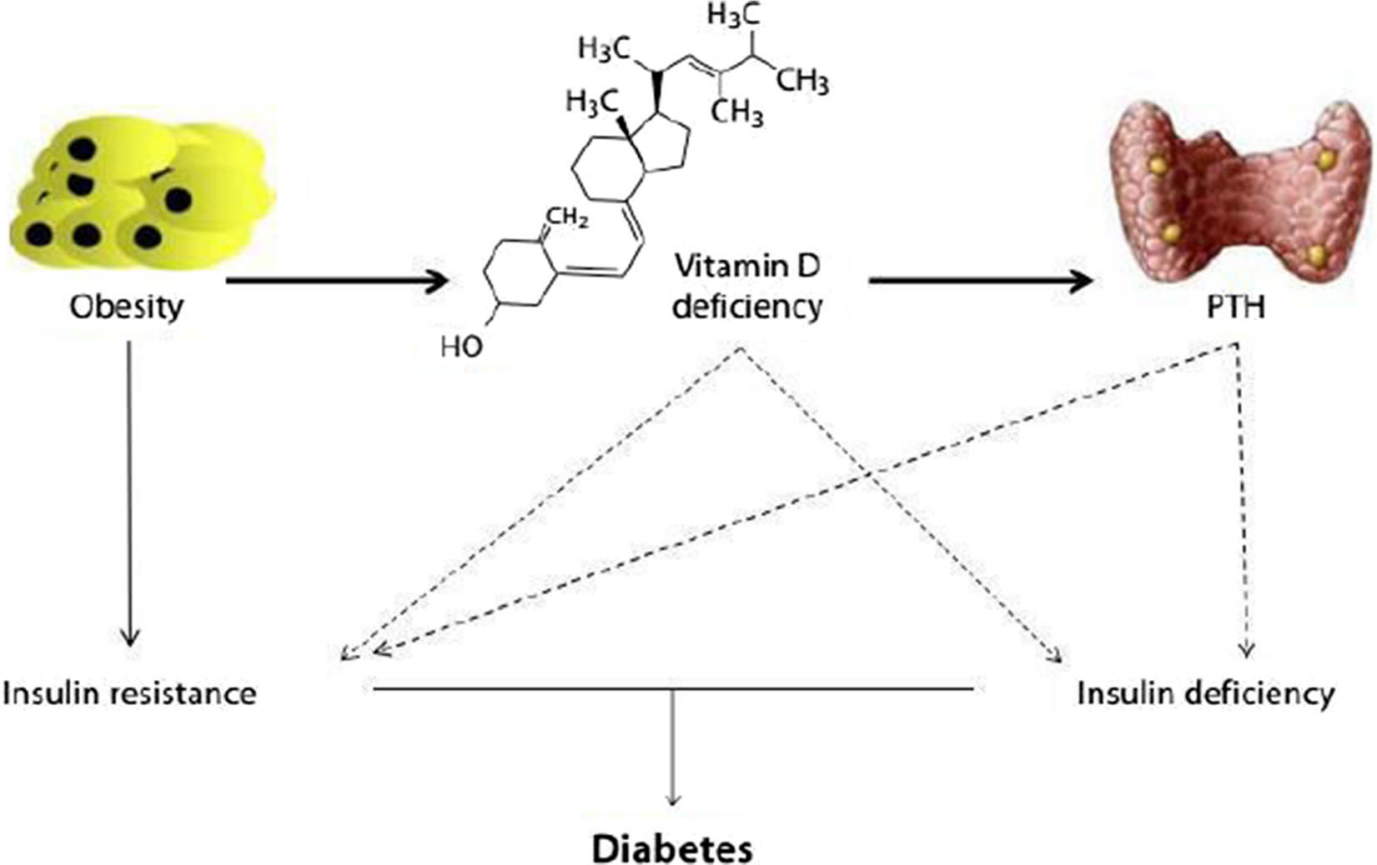


**Fig. 1** Biochemical and physiological relationship between insulin resistance, T2DM and vitamin D

**Homocysteine: risk factor for coronary artery disease in patients with type 2 diabetes mellitus**

Marija Krstevska*^1^, Ksenija Bogoeva Kostovska^2^, Katerina Tosheska Tajkovska^1^, Irena Kostovska^1^

^1^University “Ss Cyril and Methodius” Medical Faculty, Department of Medical Experimental Biochemistry, Skopje R of Macedonia

^2^Clinic of Endocrinology, Clinical Centre, Skopje, R of Macedonia

*Correspondence: Prof. Marija Krstevska, ^1^University “Ss Cyril and Methodius” Medical Faculty, Department of Medical Experimental Biochemistry, 50 Divizija 6, Skopje R of Macedonia; e-mail: krstevskam52@gmail.com

**Keywords**: Homocysteine, Diabetes mellitus, Coronary artery disease, HbA1c

**Abstract**

In resent years, plasma homocysteine (Hcy) level has been reported to be associated with vascular complications for patients with type 2 diabetes mellitus (DM2). In such patients, elevated Hcy levels were associated with insulin resistance and nephropathy.

**Objective**

To investigate the association of hyperhomocysteinemia with macro- and microvascular complications in patients with DM2 in a Macedonian population.

**Materials and methods**

In this study, 80 DM2 patients were enrolled for study and were classified into two groups: 30 patients with no associated complications (control), and 50 patients with complications, with diagnosed CAD. and with microalbuminuria. Homocysteine levels and HbA1c were determined in both investigated groups.

**Results**

Hcy levels were significantly higher (**p**<0.000) in DM2 with complications, CAD (*p*<0.000), and patients with microalbuminuria (*p*<0.000) compared with controls. There was a positive correlation between elevated HbA1c (*r* = 0.475) levels and Hcy concentration in DM2 patients.

**Conclusion**

The results show that homocysteine levels were higher in patients with micro- or macrovascular complications, and they were strongly positive correlated with HbA1c. The results also reveal that hyperhomocysteinemia is a risk factor in the etiology of vascular complications in DM2 patients.

**Integrative selection of oral hygiene complex for pregnant women with diabetes mellitus and periodontal disease**

Orekhova Liudmila^1,2^, Musaeva Ramilya^1^, Aleksandrova Anna ^1^, Posokhova Eleonora ^2^.

^1^ FSBEI HE I.P.PavlovSPbSMU MOH

^2^ City Periodontal Center “Paks” Ltd.

*Correspondence: doctor Posokhova Eleonora, City Periodontal Center “Paks” Ltd., Russia, Saint-Petersburg, 197198 Dobrolubova prospect, 27, lit.A, 3H., e-mail: posokhova_eleonora@mail.ru

**Keywords:** Individual oral hygiene, Pregnancy, Diabetes mellitus, Periodontal disease

**Abstract**

**Introduction.** It is scientifically proven that pregnant women with diabetes mellitus have a high risk of developing periodontal diseases, but prevention and treatment is difficult in this group of patients.

*Objective:* To investigate and analyze dental status of pregnant women with diabetes mellitus, to estimate the effect of individual oral hygiene complex on periodontal tissues in this group of patients.

*Materials and methods:* A clinical and laboratory examination of the oral cavity was carried out among110 patients with different types of diabetes mellitus and those without it. Women were randomly separated into three groups depending on the proposed complex of individual oral hygiene. Patients of groups 1 and 2 were trained in cleaning teeth and using extra oral hygiene products. The first group used toothpaste with herbal extracts; the second group used toothpaste with herbal extracts, sodium bicarbonate and sodium fluoride. The third group continued oral care as always. After 2 weeks the results were evaluated.

*Results.* The study revealed a correlation between values of dental indices in these groups of patients. The highest intensity of caries, values of hygiene and periodontal indices were found in women with diabetes, especially with type 2 diabetes (TDI: 17.5%, OHI-S: 1.67%, PMA: 37.4%). The proposed complexes of individual oral hygiene demonstrated their effectiveness in groups 1 and 2.

*Conclusion:* The results of the study dictate a need for developing schemes of individual oral hygiene complex based on the results of a full dental examination, including clinical and laboratory research.

**PPPM IN CARDIOVASCULAR DISEASE**

**Role of endothelial IkB kinase 2 in atherosclerosis**

Marion Mussbacher^1^, Manuel Salzmann^1^, José Basílio^1^, Mario Kuttke^1^, Hans Volek^1^, Bastian Hösel^1^, Ulrike Resch^1^ Alice Assinger^1^ Johannes Schmid^1^*

^1^Institute for Vascular Biology and Thrombosis Research, Medical University of Vienna, Vienna, Austria

*Correspondence: Ao.Univ.Prof. DI Dr. Johannes A. Schmid, Institute for Vascular Biology and Thrombosis Research, Medical University of Vienna, Vienna, Austria; e-mail: jose.basilio@meduniwien.ac.at

**Keywords**: Atherosclerosis, NF-κB, Inflammation

**Abstract**

Transcription factor NF-κB plays a key role in inflammation and is also an important regulator of genes involved in coagulation and thrombosis. NF-κB is activated by a number of signaling pathways that converge, in the majority of cases, at the level of IκB kinase 2 (IKK2). Our study uses constitutive active IKK2 (caIKK2) in a conditional transgene mouse model to mimic chronic inflammation specifically in endothelial cells and to test a potential aggravating impact on the onset of atherosclerosis.

Mice bearing inducible, aortic-EC-specific Cre recombinase on an ApoE-deficient background were crossed with a strain expressing caIKK2 downstream of a loxP-flanked stop cassette and fed a cholesterol-rich high-fat diet for 12 weeks. RNA sequencing analysis of the aortic transcriptome was performed 10 days after induction of Cre recombinase by tamoxifen, before starting the western-type diet, and revealed activation of inflammatory networks (involving TNF, IFNγ and IL1β), which were accompanied by increased immune cell infiltration. Flow cytometric analysis of lymph nodes and splenic tissue was used to detect infiltration and activation of B and T cells.

In summary, we conclude that endothelial NF-κB signaling orchestrates immune cell responses within the arterial wall and therefore plays an important role in the pathogenesis of atherosclerosis. Furthermore, our data indicate that inflammatory endothelial cell activation causes early B-cell and T-cell responses in the pathogenesis of atherosclerosis before the occurrence of cholesterol-rich lesions.

**References**

1. Hoesel B, Schmid JA. The complexity of NF-kappaB signaling in inflammation and cancer. Molecular cancer 2013, 12:86.
2. Hansson GK, Hermansson A. The immune system in atherosclerosis. Nat Immunol 2011, 12(3):204-212.
3. Tabas I, Garcia-Cardena G, Owens GK. Recent insights into the cellular biology of atherosclerosis. J Cell Biol 2015, 209(1):13-22.
4. Mohanta SK, Yin C, Peng L, Srikakulapu P, Bontha V, Hu D, Weih F, Weber C, Gerdes N, Habenicht AJ. Artery tertiary lymphoid organs contribute to innate and adaptive immune responses in advanced mouse atherosclerosis. Circ Res 2014, 114(11):1772-1787.
5. Yin C, Mohanta SK, Srikakulapu P, Weber C, Habenicht AJ. Artery Tertiary Lymphoid Organs: Powerhouses of Atherosclerosis Immunity. Frontiers in immunology 2016, 7:387

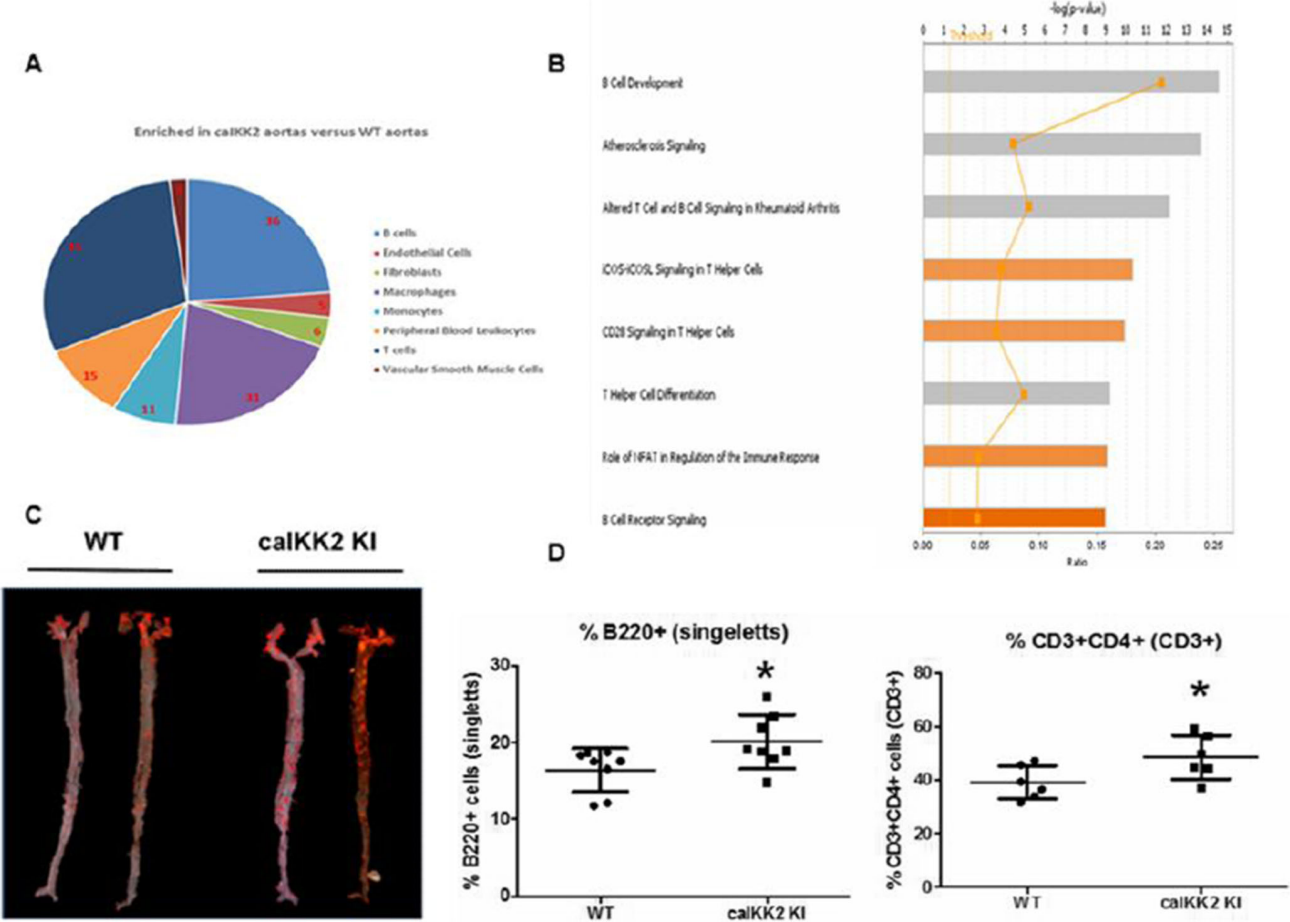


**Fig. 1** RNA-Seq analysis was performed 10 days after induction of gene expression in aortas of BMX caIKK2 mice. Clustering of cell type-specific genes using Ingenuity Pathway Analysis (IPA) revealed massive infiltration of T lymphocytes, macrophages, and B lymphocytes. Also depicted are the number of genes in each cell type (A). To get a better molecular understanding of the effect of caIKK2 in ECs, IPA was used to identify canonical pathways (B). BMX caIKK2 KI mice were crossed on APOE-deficient background and treated with a western-type diet for 12 weeks. En face preparation of isolated aortas revealed increased plaque areas in caIKK2 KI mice (C). Draining lymph nodes of the aortas of caIKK2 KI mice showed increased percentages of CD3+CD4+ and B220+ cells (D).

**Relationship between single-nucleotide polymorphisms of** ***NOTCH1*** **gene target sequence and congenital heart disease**

Dong Li*, Long Ji, Haifeng Hou, Kai Zhu, Qian Wang

School of Public Health, Taishan Medical University, China

*Correspondence: Prof. Dr. Dong Li, 619 Changcheng Road, Taian, 271000, China; email: dli@tsmc.edu.cn

**Keywords**: Congenital heart disease, single-nucleotide polymorphism, NOTCH1

**Abstract**

**Objective:** This study aimed to evaluate the relationship between the target sequence SNPs in a 3′ untranslated region (3′ UTR) of the *NOTCHI1* gene and susceptibility to congenital heart disease (CHD), and to explore the functional role of the *NOTCHI1* gene in CHD.

**Methods:** Functional SNPs in a *NOTCH1* target sequence were screened using bioinformatics databases. A case–control study of 350 children with CHD and 430 controls was conducted to analyse the association between rs6563 SNPs and CHDs. *NOTCH1* mild and mutant recombinant expression vectors were constructed by a luciferase reporter gene system. The effects of relevant miRNA on gene regulatory effects were also analysed.

**Results:** The allelic or genotypic distributions at the locus of rs6563 showed statistically significant susceptibility to CHD (odds ratio [OR] = 1.502, 95% CI: 1.209–1.866, *P*<0.001). Compared to subjects of G/G genotype, individuals with G/A or A/A genotype showed the ORs of 1.414 (95% CI: 1.047–1.908, *P*=0.02) and 2.366 (95% CI: 1.430–3.914, *P*<0.001), respectively. miR-3691-3p reduced the luciferase activity of the A allele, but had no effect on the activity of the G allele. This result indicates that the mutation from G to A enhances the negative regulation of miR-3691-3p to *NOTCH1*, contributing to the development of CHD.

**Conclusions:** The polymorphism of rs6563G>A is related to hereditary susceptibility to CHD. The mutation of G>A may affect the regulation of miR-3691-3p to the *NOTCH1* gene and alter CHD susceptibility.

**References**

1. Yang Z, Kaye DM. Mechanistic insights into the link between a polymorphism of the 3′ UTR of the SLC7A1 gene and hypertension. Human mutation. 2009, 30(3):328-333.
2. Moore MJ. From birth to death: the complex lives of eukaryotic mRNAs. Science. 2005, 309(5740):1514-1518.
3. Kratsios P, Catela C, Salimova E, et al. Distinct roles for cell-autonomous Notch signaling in cardiomyocytes of the embryonic and adult heart. Circulation research. 2010, 106(3):559-572.
4. Yamakuchi M, Ferlito M, Lowenstein C J. miR-34a repression of SIRT1 regulates apoptosis. Proceedings of the National Academy of Sciences. 2008, 105(36):13421-13426.
5. Zhao JY, Qiao B, Duan WY, et al. Genetic variants reducing MTR gene expression increase the risk of congenital heart disease in Han Chinese populations. European heart journal. 2014, 35(11):733-742.

**Association between obstructive sleep apnea and hypertension: a systematic review and meta-analysis**

Haifeng Hou^1, 2*^, Yange Zhao^3^, Wenqing Yu^3^, Hualei Dong^4^, Xiaotong Xue^4^, Jian Ding^4^, Wijia Xing^1^, Wei Wang^1, 2*^

^1^ School of Public Health, Taishan Medical University, Taian, China

^2^ School of Medical and Health Sciences, Edith Cowan University, Perth, Australia

^3^ School of Basic Medical Science, Taishan Medical University, Taian, China

^4^ Taishan Hospital of Shandong Province, Taian, China

*Correspondence: Dr. Haifeng Hou, School of Public Health, Taishan Medical University, 619 Changcheng Road, Taian, 271000, China; email: hfhou@tsmc.edu.cn

Prof. Wei Wang, School of Medical and Health Sciences, Edith Cowan University, Perth, WA 6027, Australia; email: wei.wang@ecu.edu.au

**Keywords**: Obstructive sleep apnea, Hypertension, Systematic review, Meta-analysis

**Abstract**

**Background:** Obstructive sleep apnea (OSA) is a sleep disorder characterized by complete or partial upper airflow cessation during sleep, which is reported to affect approximately 17% of American adults [1-3]. Although it has been widely accepted that OSA is a risk factor for the development of hypertension, studies focusing on this topic have revealed inconsistent results [2-4]. We aimed to clarify the association between OSA and hypertension, including essential and medication-resistant hypertension.

**Methods:** The Preferred Reporting Items for Systematic Reviews and Meta-Analyses (PRISMA) was followed [5]. PubMed and Embase databases were used to search for relevant studies published up to December 31, 2016. A quantitative approach using meta-analysis was employed to estimate the pooled odds ratio (OR) and 95% confidence interval (CI).

**Results:** Twenty-six studies with a total of 51,623 participants (28, 314 male/ 23, 309 female; mean age 51.8 years) met the inclusion criteria and were included in this study. Among these, six studies showed a significant association between OSA and resistant hypertension (pooled OR = 2.842, 95% CI = 1.703–3.980, *P*<0.05). In addition, a combination of 20 original studies on the association between OSA and essential hypertension presented significant results, with pooled ORs of 1.184 (95% CI = 1.093–1.274, *P*<0.05) for mild OSA, 1.316 (95% CI: 1.197–1.433, *P*<0.05) for moderate OSA and 1.561 (95% CI: 1.287–1.835, *P*<0.05) for severe OSA.

**Conclusions:** Our findings indicated that OSA is related to an increased risk of resistant hypertension. Mild, moderate and severe OSA are associated with essential hypertension, which manifests a dose–response relationship. The associations are stronger among whites and male OSA patients.

**References**

1. Walia HK, Li H, Rueschman M, Bhatt DL, Patel SR, Quan SF, et al. Association of severe obstructive sleep apnea and elevated blood pressure despite antihypertensive medication use. J Clin Sleep Med. 2014; 10: 835 - 43.
2. Nieto FJ, Young TB, Lind BK, Shahar E, Samet JM, Redline S, et al. Association of sleep-disordered breathing, sleep apnea, and hypertension in a large community-based study. Sleep Heart Health Study. JAMA 2000; 283: 1829-36.
3. Marin JM, Agusti A, Villar I, Forner M, Nieto D, Carrizo SJ, et al. Association between treated and untreated obstructive sleep apnea and risk of hypertension. JAMA.2012 307 (20):2169-76.
4. Appleton SL, Vakulin A, Martin SA, Lang CJ, Wittert GA, Taylor AW, et al. Hypertension is associated with undiagnosed obstructive sleep apnea during rapid eye movement (REM) sleep. Chest. 2016; 150:495-05.
5. Hou H, Sun T, Li C, Li Y, Guo Z, Wang W, et al. An overall and dose-response meta-analysis of red blood cell distribution width and CVD outcomes. Sci Rep. 2017; 7:43420.

**Quality of life and cold extremities in Korea**

Kwang-Ho Bae^1*^, Ki-Hyun Park^1^, Su-Jung Kim^1^, and Si-woo Lee^1^

^1^ Mibyeong Research Center, Korea Institute of Oriental Medicine, Republic of Korea

*Correspondence: Dr. Kwang-Ho Bae, Mibyeong Research Center, Korea Institute of Oriental Medicine, 1672 Yuseong-daero, Yuseong-gu, Daejeon, 34054, Republic of Korea; e.mail: solarhuman@kiom.re.kr

**Keywords:** Cold hypersensitivity in the hands and feet, Korean medicine, Cold extremities, Quality of life, Cold disorder

**Abstract**

**Introduction:** Cold extremities (cold hypersensitivity in the hands and feet, CE) is a common symptom in East Asia, including Korea [1, 2]. In Korean medicine, CE is used for pattern identification and is believed to be associated with various symptoms and diseases [3, 4]. This study aimed to investigate whether quality of life (QOL) differs depending on the presence or absence of CE.

**Methods:** In 2013, a national survey was conducted among the general population through a specialized survey institute. Subjects were selected randomly using a multistage stratified sampling method based on distribution by region, gender, and age (95% confidence level, sample error ± 3.0%). A total of 1101 subjects aged 20–84 years were asked to complete a questionnaire evaluating QOL (SF-12) and the extent of cold extremities [5]. Multiple regression analysis was used to evaluate the correlation between CE and QOL score, with adjustment for age, gender, and body mass index (BMI).

**Results:** Among participants, 18.6% (*n* = 208) demonstrated CE and 72.7% (*n* = 800) no CE. CE was more prevalent in women than men, and those with warm hands and feet had higher BMI than those with cold hands and feet, regardless of gender or age. In multiple regression analysis, CE showed a significant independent effect on the SF-12 physical component summary (β = −0.11, *p* < 0.001) and mental component summary (β = −0.10, *p* < 0.01) when we adjusted for gender, age, and BMI.

**Conclusion:** This study showed that CE demonstrated significant independent effects on health-related QOL indicators.

**References**

1. Hur Y-M, Chae JH, Chung KW, Kim JJ, Jeong HU, Kim JW, Seo SY, Kim KS: Feeling of Cold Hands and Feet is a Highly Heritable Phenotype. Twin Res Hum Genet. 2012;15:166-169. doi: 10.1375/twin.15.2.166
2. Yoshino T, Katayama K, Munakata K, Horiba Y, Yamaguchi R, Imoto S, Miyano S, Watanabe K: Statistical analysis of hie (cold sensation) and hiesho (cold disorder) in kampo clinic. Evid Based Complement Alternat Med. 2013;398458. doi: 10.1155/2013/398458
3. Bae KH, Lee JA, Park KH, Yoo JH, Lee Y, Lee S: Cold Hypersensitivity in the Hands and Feet May Be Associated with Functional Dyspepsia: Results of a Multicenter Survey Study. Evid Based Complement Alternat Med. 2016;8948690. doi: 10.1155/2016/8948690
4. Konieczka K, Ritch R, Traverso CE, Kim DM, Kook MS, Gallino A, Golubnitschaja O, Erb C, Reitsamer HA, Kida T et al: Flammer syndrome. EPMA J. 2014;5:11. doi: 10.1186/1878-5085-5-11
5. Ware Jr JE, Kosinski M, Keller SD: A 12-Item Short-Form Health Survey: construction of scales and preliminary tests of reliability and validity. Med care. 1996;34(3):220-233.

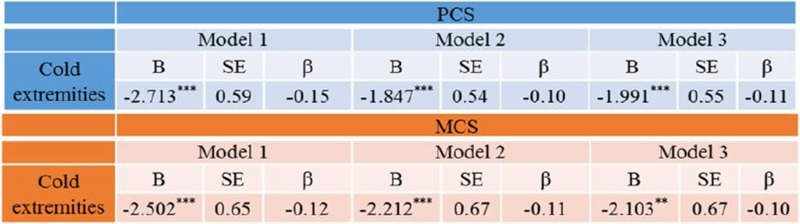


**Fig. 1** Multiple regression analysis for the association between cold extremities and QOL

Model 1: unadjusted. Model 2: adjusted for gender and age. Model 3: adjusted for gender, age, body mass index. B, unstandardized regression coefficients; SE, standard error of unstandardized coefficients; β, standardized regression coefficients; QOL, quality of life; PCS, SF-12 Physical Component Summary; MCS, SF-12 Mental Component Summary. *** *p* < 0.001; ** *p* < 0.01

**Cardiologic aspects of preventive management during pregnancy: assessment of central pressure**

Maria Evsevyeva* ^1^, Oksana Sergeeva^1^ , Irina Prokhorenko -Kolomoytsev^2^, Kirill Pavlov^3^

^1^Department of Internal Diseases, Stavropol State Medical University, Russian Federation ^2^Stavropol Regional Endocrinological Dispensary, Russian Federation

^3^Stavropol Regional Perinatal Center, Russian Federation

*Correspondence: Maria Evsevyeva, e.mail: evsevieva@mail.ru 

**Keywords:** Pregnancy, Central pressure, Preventive management, Arterial hypertension

**Abstract**

**Background.** Pregnancy can function as a load test for identifying women at high cardiovascular (CV) risk [1]. Improvement is needed in the system of preclinical diagnosis of CV disorders in pregnants [2].

**Objective.** To assess the significance of the parameters of central aortic pressure (CAP) and aortic pulse wave in the overall cardiac examination of pregnants with respect to preeclampsia (PE).

**Material and methods.** A total of 253 pregnant women were surveyed, in whom risk factors and CV activity was evaluated using the BPLab diagnostic complex (Petr Telegin, Russia) with Vasotens Office software [3]. A comparison of central and peripheral pressure made it possible to identify various options for hypertension (H) – hidden, false and systemic. The results were processed using Statistica 8.0 software.

**Results.** The occurrence of false H was seen in 2.75%, systemic H in 2.75%, and hidden H in 7.5% of cases. The augmentation index in women with hidden H did not differ from those with systemic H. The incidence of PE in normotensive women was 0.9%, with systemic H in 14.3% and hidden H in 10.5%. In patients with false H, such cases were generally not encountered.

**Conclusion.** The data indicate the feasibility of including an assessment of CAP in CV examination protocols for pregnants [4,5]: first, the high prognostic value of this index was confirmed, and second, hidden AG was discovered more frequently in pregnant women than systemic and false forms of pressure buildup. The significance of this problem is evidenced by the increased aortic rigidity and more frequent development of PE.

**References**

1. Heart Disease in Pregnancy. Ed. by Adamson D., Dhanjal M., Nelson-Piercy C. Oxford University Press, 2011.
2. Compendium for the Antenatal Care of High-Risk Pregnancies. Ed. by Narayan H. Oxford University Press, 2015
3. Evsevyeva M., Eremin M., Rostovtseva M., Koshel V., Shchetinin E.: Evaluation of the central pressure and preventive examination of student youth. European Journal of Preventive Cardiology 2017, 24(S1С): S170.
4. Treating Women with Substance Use Disorders During Pregnancy. A Comprehensive Approach to Caring for Mother and Child. Ed. by Jones H.E., Kaltenbach K.2013
5. Evsevyeva M., Sergeeva O., Pavlov K., Prokhorenko-Kolomoytseva I. Pregnancy and detection of latent hypertension. Journal of Hypertension 2016, 34(S1С): e308

**Prevention of cardiovascular diseases in patients with somatoform dysfunction of the vegetative nervous system**

Е.Y. Esina ^1^ *, V.V. Lyutov ^2^, V.N. Tsigan ^3^, A.A. Zuykova ^1^

^1^ Voronezh State Medical University named after N.N. Burdenko Health Ministry of Russia, Voronezh, Russia

^2^ 442 Military Clinical Hospital, Ministry of Defence of the Russian Federation, St. Petersburg, Russia

^3^ Military - Medical Academy named after S.M. Kirov, Ministry of Defence of the Russian Federation, St. Petersburg, Russia

*Correspondence: PhD., Е.Y. Esina, Voronezh N.N. Burdenko State Medical University, Voronezh, Russia, Avenue of Revolution 14, 394036 Voronezh, Russia; e-mail: micvsma@yandex.ru

**Keywords:** Prevention of cardiovascular diseases, Somatoform dysfunction of the vegetative nervous system

**Abstract**

Studies are lacking as to whether somatoform vegetative nervous system dysfunction (SVNSD) is a premorbid condition for cardiovascular disease (CVD). The present study involved 259 patients with SVNSD and CVD risk factors (RF), mean age 22.8 ± 1.6 years. Dispersion mapping of the electrocardiogram was used. A factor analysis was carried out using the principal components method. The criterion for the adequacy of the Kaiser-Meier-Olkin sample was 0.664. The Bartlett sphericity criterion was (χ2 (45) = 826.555, *p* <0.001). Three factors with proper values greater than 1 were extracted. The first factor (F1) – metabolic – combined systolic blood pressure 0.866, diastolic arterial pressure 0.871, and excess body weight and obesity 0.792. The second factor (F2) – psychoemotional – combined psychosocial stress 0.855, anxiety 0.827, and depression 0.868. The third factor (F3) – the prenozological changes in the electrophysiological state of the myocardium (EPhSM) – combined the variables: office heart rate 0.820; the total value of the dispersion deviations from the norm after an exercise load of 0.434. The duration of RRNN after 4 minutes of orthostasis was −0.869; the total value of the dispersion deviations from the norm after 4 minutes of orthostasis was 0.576. Factor analysis made it possible to establish biopsychosocial interrelationships among the psychosocial, biological RF of the CVD and the EPhSM.

**The importance of routinely reporting non-HDL cholesterol by the clinical laboratory**

Katerina Tosheska-Trajkovska^1^, Irena Kostovska^1^ and Gordana Bosilkova^1^

^1^Department of Medical and Experimental Biochemistry, Medical Faculty, Skopje, Macedonia

*Correspondence: eng. chem. Gordana Bosilkova, ^1^Department of Medical and Experimental Biochemistry, Medical Faculty, 50 divizija 6, 1000 Skopje, Macedonia; e-mail: gbosilkova@yahoo.com

**Keywords:** Non-HDL cholesterol, Predictor, Blood lipids, Heart disease, Laboratory medicine report

**Abstract**

Background. It is well known that a standard lipid profile measures total cholesterol, triglycerides, and HDL cholesterol (HDL-c), while LDL cholesterol is estimated. Subtracting HDL-c from total cholesterol, we can measure the amount of cholesterol carried by all lipoproteins except HDL. This simple math will give the amount of non-HDL cholesterol (non-HDL-c) within all atherogenic lipoproteins. However, although it is so easy, biochemical laboratories rarely report the value of non-HDL-c.

Aim of the study. The aim of our study is to highlight the results of several studies that clearly establish the clinical use of non–HDL cholesterol as a common predictor of blood lipid patterns associated with increased risk of heart disease.

Material. Recent literary evidence and clinical studies.

Results. The most important document regarding non-HDL-c was delivered by the National Cholesterol Education Program Adult Treatment Panel III, where non–HDL-c was highlighted as a key secondary goal of therapy in lipid treatment and lipoprotein management. Other studies suggest that non-HDL-c shows a better correlation with small dense LDL particles than do other lipid parameters, including LDL-c**.** The treatment goal for non-HDL-c is usually 30 mg/dL above the LDL-c treatment target.

Conclusion. Knowing that non–HDL-c is superior to LDL-c for the prediction of cardiovascular events, it is very important for each clinical chemistry laboratory to report non–HDL-c as a part of routine lipid status, with no added expense. Doctors could use it for optimal prevention of atherosclerosis and cardiovascular disease.

**Nephrin: marker for early detection of hypertensive nephropathy**

*Irena Kostovska ^1,^ Katerina Tosheska Trajkovska^1^ , Svetlana Cekovska^1^ , Goce Spasovski^2^ , Danica Labudovik^1^

^1^ Department of medical and experimental biochemistry, Ss. Cyril and Methodius University, Skopje, Macedonia

^2^ Department of Nephrology, Ss. Cyril and Methodius University, Skopje, Macedonia

*Correspondence: MD, PhD student Irena Kostovska, Specialist in medical biochemistry, Department of medical and experimental biochemistry, Medical faculty, Ss. Cyril and Methodius University, 50 Divizija 6, 1000 Skopje, Macedonia

e-mail:irenakostovska22@yahoo.com

**Keywords:** Nephrin, Microalbumin, Chronic hypertension, Hypertensive nephropathy

**Abstract**

**Introduction:** Hypertensive nephropathy (HN) is a medical condition characterized by damage to the kidney due to chronic high blood pressure, and it is second common cause of end-stage renal disease. Podocytopathies have a role in pathogenesis of HN, thus the podocyte proteins may have significance in early detection of disease. The purpose of this paper is to test the significance of nephrin as a marker for early detection of HN.

**Material and methods:** This study included 64 people with chronic hypertension (CH; 33 male/31 female) with an average age of 54.1 ± 5.1 years. Thirty healthy subjects (10 male/20 female) with an average age of 48.7 ± 9.4 years were included as a control group. All patients were divided into three groups: patients with normoalbuminuria, microalbuminuria and macroalbuminuria, according to urinary microalbumin/creatinine ratios. As material, we used fresh urine and venous blood. In urine we measured nephrin by the ELISA method, creatinine photometrically and microalbumin turbidimetrically. In blood we measured urea, creatinine, albumin and total protein using a photometric method.

**Results:** In 69.7% of normoalbuminuric subjects with CH, we found elevated urinary nephrin. Nephrin was significantly elevated in all groups of participants with HN compared with the control group (*p*<0.05). Nephrin was significantly elevated in patients with normoalbuminuria compared with the control group (*p*<0.05). We found a positive correlation between urinary concentration of nephrin and serum creatinine and a negative correlation between urinary nephrin and eGFR.

**Conclusion:** Nephrin can be a useful marker for early and non-invasive detection of HN.

**References**

1. Endlich N, Kress KR, Reiser J et al. Podocytes respond to mechanical stress in vitro. J Am Soc Nephrol. 2001;12(3):413–422.
2. Seccia TM, Caroccia B, Calò LA. Hypertensive nephropathy. Moving from classic to emerging pathogenetic mechanisms. J Hypertens. 2017;35(2):205-212.
3. Fogo A, Breyer JA, Smith MC et al. Accuracy of the diagnosis of hypertensive nephrosclerosis in African Americans: a report from the African American Study of Kidney Disease (AASK) trial. AASK pilot study investigators. Kidney Int 1997; 51: 244–252.
4. Wang G Lai, FM Kwan, BC Lai et al. Podocyte loss in human hypertensive nephrosclerosis. Am J Hypertens 2009;22:300–306.
5. Kretzler M, Koeppen-Hagemann I, Kriz W. Podocyte damage is a critical step in the development of glomerulosclerosis in the uninephrectomised-desoxycorticosterone hypertensive rat. Virchows Arch. 1994; 425: 181–193.

**INNOVATIVE TECHNOLOGIES IN PPPM**

**A pilot study on the investigation of tear biomarkers as an indicator of human health**

Stephen Morton^1^, Brian Crucian^1^, Susan Steinberg^2^, Suzanne Hagan^3,^ Bethany Tucker^4^

^1^ NASA Johnson Space Center, NASA Parkway, Houston, Texas, 77058 USA

^2^ KBRwyle, NASA Parkway, Houston, Texas, 77058 USA

^3^ Vision Sciences, Glasgow Caledonian University, Scotland, UK;

^4^ 10700 Santa Monica BLVD. Suite 300, Los Angeles, Ca. 90025 USA

*Correspondence: Stephen Morton, Mail Code: SD, 2101 NASA Parkway, Houston, Texas 77058, email address: steven.g.morton@nasa.gov

**Keywords:** Tear fluid, Biomarkers, Diagnosis, Inflammation, Ocular, Brain health

**Abstract**

The scientific literature suggests that tear biomarkers can be used as a guide towards clinical diagnosis of human health [1]. This study will investigate whether tear biomarkers represent a research and clinical opportunity to assess human health prior to, during, and after exposure to the spaceflight environment. The focus of this study is to compare biomarkers previously identified as potentially relevant to both ocular and brain health against unique physiological outcomes of exposure to the spaceflight environment.

Study subjects suffering from terrestrial conditions thought to be similar to spaceflight-associated neuro-ocular syndrome (SANS: formerly VIIP), e.g. patients with idiopathic intracranial hypertension (IIH) and optic neuritis, may be relevant to conditions associated with spaceflight. This study will review methodologies, tear biomarkers related to the state of ocular and brain health, and the strengths and weakness of using tear fluid biomarkers versus other body fluid samples, and will survey current tear fluid biomarker knowledge in research and clinical practice.

A strength of using tear biomarkers is that sampling is non-invasive and is used as a guide in understanding pathologies, including ocular and systemic inflammatory conditions [2]. Moreover, tear biomarkers may reflect diseases affecting the central nervous system (CNS) [3]. For example, in multiple sclerosis (MS), the concordance rate between tear biomarkers and cerebrospinal fluid (CSF) is approximately 83%, indicating that in the majority of cases, tears are at least as effective as CSF in potentially identifying novel MS biomarkers [4].

**References**

1. Hagan S, Martin E, Enríquez-de-Salamanca A. Tear fluid biomarkers in ocular and systemic disease: potential use for predictive, preventive and personalised medicine. EPMA J. 2016;7:15. doi:10.1186/s13167-016-0065-3.
2. Cocho L, Fernández I, Calonge M, Martínez V, González-García MJ, Caballero D, López-Corral L, García-Vázquez C, Vázquez L, Stern ME, Enríquez-de-Salamanca A. Biomarkers in Ocular Chronic Graft Versus Host Disease: Tear Cytokine- and Chemokine-Based Predictive Model. Invest Ophthalmol Vis Sci. 2016; 57(2):746-58.
3. Salvisberg C, Tajouri N, Hainard A, Burkhard PR, Lalive PH, Turck N. Exploring the human tear fluid: discovery of new biomarkers in multiple sclerosis. Proteomics-Clinical Applications, 2014; 8(3-4):185-194.
4. Devos D, Forzy G, de Seze J, Caillez S, Louchart P, Gallois P, Hautecoeur P. Silver stained isoelectrophoresis of tears and cerebrospinal fluid in multiple sclerosis. J. Neurology.> 2001; 248(8):672-675.

**Identification of biomarkers of ocular surface disease using impression cytology**

Suzanne Hagan^1^, Boatemaa Omotayo^1^, Katherine Oliver^1^, Michael Doughty^1^, Claire Walshe^2^.

^1^Vision Sciences, School of Health and Life Sciences, Glasgow Caledonian University (GCU), Scotland, UK; ^2^Topivert Pharma Ltd., London, UK.

*Correspondence *Dr Suzanne Hagan, Room A020, Vision Sciences, Dept of Life Sciences, GCU, G4 0BA, UK; email: suzanne.hagan@gcu.ac.uk

**Keywords:** Biomarkers, Dry eye disease, Ocular surface, Laboratory medicine, Diagnostic, Therapeutic targets

**Abstract**

Impression cytology (IC) is a method which has been used to assess ocular surface cells from patients, e.g. conjunctival and corneal epithelial cells. IC is a minimally invasive technique which utilises biopore, cellulose acetate or polyethersulfone (PES) filters to sample cells, and has traditionally been used to ascertain morphological changes to aid in the diagnosis of squamous metaplasia and dry eye disease (DED).^1,2^

More recently, IC has been utilised for the investigation of gene expression of ocular surface cells.^3^ We demonstrate the use of IC to identify gene and protein expression changes between DED subjects and healthy controls (HC).

Conjunctival epithelial cells from DED patients (*n*=8) and HC (*n*=7) were harvested by IC using the Eyeprim™ device. Quantitative PCR (qPCR) was performed to quantify differences in target gene expression: p38-α, IL-1β, IL-8, MCP-1 and MMP-9. In addition, the spatial localisation of proteins p38-α and phospho p38 were probed in immunofluorescence assays.

Using the Eyeprim device, the average yield of conjunctival epithelial cells (CEC) retrieved was 1.1 x 10^5^ cells/mm^2^. qPCR analysis demonstrated significantly higher p38-α, IL-1β, IL-8, MCP-1 (all *p*<0.0001) and MMP-9 (*p*<0.002) expression in CE from DED patients versus HC. Immunofluorescence studies significantly increased p38-α and phospho p38-α protein levels in DED subjects versus HC (*p*<0.0001 and *p*<0.01, respectively).

As IC retrieval procedures have improved and the sensitivity of detection has increased,^4^ gene and protein analysis of IC-retrieved CEC may be used to identify novel biomarkers of ocular surface diseases.

**References**

1. Lopin E, Deveney T, Asbell PA. Impression cytology: recent advances and applications in dry eye disease. Ocul Surf. 2009;7(2):93-110.
2. Doughty MJ. A Grid-Based Nucleus Counting Method for Estimates of the Density of Superficial Conjunctival Cells from Impression Cytology Samples Taken from Normal Healthy Human Eyes. Curr Eye Res. 2017; 30:1-7.
3. López-Miguel A, Gutiérrez-Gutiérrez S, García-Vázquez C, Enríquez-de-Salamanca A. RNA Collection From Human Conjunctival Epithelial Cells Obtained With a New Device for Impression Cytology. Cornea. 2017;36(1):59-63.
4. Cocho L, Fernández I, Calonge M, Martínez V, González-García MJ, Caballero D, López-Corral L, García-Vázquez C, Vázquez L, Stern ME, Enríquez-de-Salamanca A. Gene Expression-Based Predictive Models of Graft Versus Host Disease-Associated Dry Eye. Invest Ophthalmol Vis Sci. 2015;56(8):4570-81.

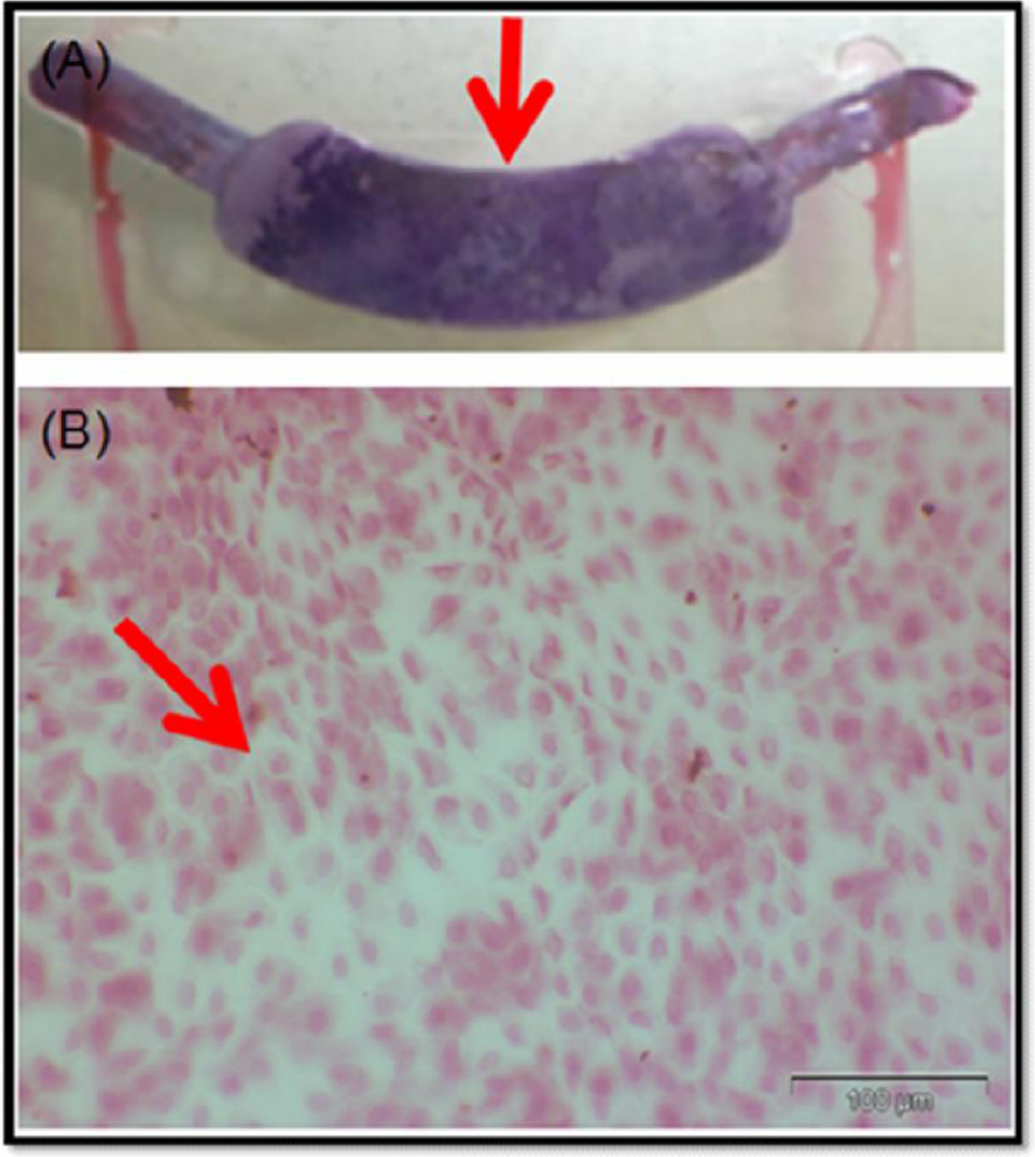


**Fig 1**. H & E staining of normal human conjunctival cells following IC (Eyeprim® membrane), where (A) shows overall coverage with normal human conjunctival cells and (B) shows the magnified structure of conjunctival cells.

**The roles of proteomic and metabolomic variations in predictive, preventive, and personalized medicine**

Xianquan Zhan^1, 2, 3, 4*^, Ying Long^1, 2, 3^, Miaolong Lu^1, 2, 3^

^1^ Key Laboratory of Cancer Proteomics of Chinese Ministry of Health, Xiangya Hospital, Central South University, 87 Xiangya Road, Changsha, Hunan 410008 P. R. China

^2^ Human Engineering Laboratory for Structural Biology and Drug Design, Xiangya Hospital, Central South University, 87 Xiangya Road, Changsha, Hunan 410008 P. R. China

^3^ State Local Joint Engineering Laboratory for Anticancer Drugs, Xiangya Hospital, Central South University, 87 Xiangya Road, Changsha, Hunan 410008 P. R. China

^4^ The State Key Laboratory of Medical Genetics, Central South University, 88 Xiangya Road, Changsha, Hunan 410008 P. R. China

*Correspondence: Xianquan Zhan, Key Laboratory of Cancer Proteomics of Chinese Ministry of Health, Xiangya Hospital, Central South University, 87 Xiangya Road, Changsha, Hunan 410008 P. R. China. Tel: 86-731-84327905; Fax: 86-731-84327905; E-mail: yjzhan2011@gmail.com.

**Keywords**: Proteomic variation, Metabolomic variation, Phenome, Omics, Predictive, preventive, and personalized medicine

**Abstract**

Omics and systems biology have driven the rapid development of predictive, preventive, and personalized medicine (PPPM). Variations exist in the entire process of healthcare. Extensive genomic, transcriptomic, peptidomic, proteomic, and metabolomic data have been achieved in the field of human omics. Of these, proteomic variations are the final presentation of the genomic and transcriptomic variations. Metabolomic variations are the comprehensive results originating from proteins, nucleic acids, sugars, and lipids. Proteins and metabolites are two essential elements to differentiate the phenotypes. Moreover, many aspects in the proteome including splicing, post-translational modifications, translocation, conformation, redistributions, and variants, and in metabolome including entire component of metabolites, pattern variations, and disease-specific metabolomic variations, remain unknown. Clarification of variations in the phenome is a key step toward realizing PPPM practice. Proteome and metabolome are important components that contribute to the phenome. Therefore, studies of variations in proteomes and metabolomes have important scientific value in PPPM, including resolution of disease molecular mechanisms, determination of effective and reliable biomarkers for personalized prediction/prevention, diagnosis/therapy, and prognostic assessment, and discovery of effective therapeutic targets to achieve precision medicine.

**References**

1. Hu R, Wang X, Zhan X: Multi-parameter systematic strategies for predictive, preventive and personalised medicine in cancer. EPMA J 2013, 4: 2.
2. Zhan X, Desiderio DM: The use of variations in proteomes to predict, prevent, and personalize treatment for clinically nonfunctional pituitary adenomas. EPMA J. 20101(3):439-59. doi:10.1007/s13167-010-0028-z.
3. Cheng T, Zhan X: Pattern recognition for predictive, preventive, and personalized medicine in cancer. EPMA J. 2017;8(1):51-60. doi:10.1007/s13167-017-0083-9.
4. Grech G, Zhan X, Yoo BC, Bubnov R, Hagan S, Danesi R, Vittadini G, Desiderio D: EPMA Position Paper in Cancer: Current overview and future perspectives. EPMA J. 2015;6(1):9. doi:10.1186/s13167-015-0030-6.
5. Golubnitschaja O, Costigliola V, EPMA: General report & recommendations in predictive, preventive and personalised medicine 2012: white paper of the European Association for Predictive, Preventive and Personalised Medicine. EPMA J. 2012;3(1):14. doi:10.1186/1878-5085-3-14.

**Profiling IgG N-glycans as biomarkers of the ageing process**

Wei Wang*^1,2^

^1^School of Medical and Health Sciences, Edith Cowan University, Perth, Australia

^2^Beijing Key Laboratory of Clinical Epidemiology, School of Public Health, Capital Medical University, Beijing, China

*Correspondence: Prof. Wei Wang, School of Medical and Health Sciences, Edith Cowan University, Perth, Australia; e.mail: wei.wang@ecu.edu.au

**Keywords**: N-glycans, Predictive biomarkers, Ageing

**Abstract**

IgG N-glycan profiles can serve as dynamic indicators of the ageing process and are able to discriminate between normal and accelerated ageing by highlighting a discrepancy between a body’s age in years of life (chronological age) and its age in terms of health status (biological age). Therefore, IgG N-glycome represents a real-time indicator of the interaction between genetic/epigenetic predisposition and the environment. IgG N-glycans serve as predictive biomarkers when health is evaluated in the context of age, based on the proposition that specific IgG N-glycans i) identify variations in responses to maturation, ageing, and environment across the life course at a population level; ii) facilitate early risk prediction of chronic disease development; and iii) serve as prognostic indicators for the benefit of targeted preventive and disease treatment interventions. A new challenge, the ability to develop an “ageing biomarker kit” to be used in a (pre)clinical setting as a tool of predictive, preventive and personalised medicine, is presented here.

**References**

1. Wang Y, Adua E, Russell AC, Roberts P, Ge S, Zeng Q et al. Glycomics and its application potential in precision medicine. Science supplement: Precision medicine in China. 2016;354(6319):36-9.
2. Yu X, Wang Y, Kristic J, Dong J, Chu X, Ge S et al. Profiling IgG N-glycans as potential biomarker of chronological and biological ages: A community-based study in a Han Chinese population. Medicine (Baltimore). 2016;95(28):e4112. doi:10.1097/MD.0000000000004112.
3. Wang Y, Klaric L, Yu X, Thaqi K, Dong J, Novokmet M et al. The Association Between Glycosylation of Immunoglobulin G and Hypertension: A Multiple Ethnic Cross-Sectional Study. Medicine (Baltimore). 2016;95(17):e3379. doi:10.1097/MD.0000000000003379
4. Vuckovic F, Kristic J, Gudelj I, Teruel M, Keser T, Pezer M et al. Association of systemic lupus erythematosus with decreased immunosuppressive potential of the IgG glycome. Arthritis Rheumatol. 2015;67(11):2978-89. doi:10.1002/art.39273.
5. Lu JP, Knezevic A, Wang YX, Rudan I, Campbell H, Zou ZK et al. Screening novel biomarkers for metabolic syndrome by profiling human plasma N-glycans in Chinese Han and Croatian populations. J Proteome Res. 2011;10(11):4959-69. doi:10.1021/pr2004067.

**Novel approach for preventing implant failure: programming of anti-inflammatory macrophages using self-standing release systems with a phenotype-fixing cytokine cocktail formulation**

Julia Kzhyshkowska ^1,2,3^ , Vladimir Riabov^1,3^, Fabián Salazar^4^, SuSu Htwe^4^, Alexandru Gudima^1^, , Julien Barthes^5^, Helena Knopf-Marques^*6,7*^, Harald Klüter^1,2^, Amir M Ghaemmaghami^4^, Nihal Engin Vrana^5,6^.

^1^Institute for Transfusion Medicine and Immunology, Medical Faculty Mannheim, University of Heidelberg, Theodor-Kutzer Ufer 1-3, 68167 Mannheim, Germany

^2^Red Cross Blood Service Baden-Württemberg–Hessen, Friedrich-Ebert Str. 107

D-68167 Mannheim, Germany

^3^Laboratory for translational cellular and molecular biomedicine, Tomsk State University, 36 Lenin Prospekt, Tomsk, 634050, Russia

^4^Division of Immunology, Queen's Medical Centre, School of Life Sciences, Faculty of Medicine and Health Sciences, University of Nottingham, Nottingham NG7 2UH, UK

^5^Protip Medical, 8 Place de l’Hopital, 67000, Strasbourg, France

^6^INSERM UMR 1121, Biomaterials and Bioengineering”, 11 rue Humann, 67000, Strasbourg, France ^7^Faculté de Chirurgie Dentaire, Université de Strasbourg, 3 rue Sainte Elisabeth, 67000 Strasbourg, France

*Correspondence: Prof. Dr. Julia Kzhyshkowska, Institute of Transfusion Medicine and Immunology, Medical Faculty Mannheim, Heidelberg University; Ludolf-Krehl Strasse 13-17, D-68167 Mannheim; e.mail: julia.kzhyshkowska@medma.uni-heidelberg.de

**Abstract**

Nondegradable metallic and polymeric implants are used as mainstream solutions in regenerative medicine. Chronic inflammation is the most common complication leading to implant intolerance and failure. Key cells that control local inflammation and foreign body response are tissue macrophages (1). After implantation, macrophages induce acute inflammatory reactions to trauma and foreign material, which must be followed by the resolution of inflammation, wound healing phase and restoration of homeostasis. Personalized therapeutic solutions are required to improve implant integration and acceptance. Macrophages are highly plastic cells that define the reactions of the immediate tissue microenvironment to the implanted material. However, frustrated phagocytosis and inability of macrophages to resolve inflammation and restore tissue-specific homeostatic balance can lead to the chronic inflammation around implants. The prevention of implant-induced chronic inflammation by programming of the macrophage phenotype is a promising strategy for improving implant acceptance. The aims of such programming include controlling the phenotype of local macrophages by long-term fixation of their healing activities and suppression of inflammatory reactions. We have developed a cytokine cocktail formula (M2Ct) that induces a stable M2-type phenotype in human primary macrophages characterized by significant suppression of induced pro-inflammatory reactions and increased secretion of anti-inflammatory cytokines (M2Ct) (2). The positive effect of the M2Ct was demonstrated in an in vitro wound healing model. Using this model for induction of inflammation by LPS, we demonstrated that the M2Ct phenotype was stable for at least 12 days. However, in the absence of M2Ct components in the medium, macrophages underwent rapid pro-inflammatory reprogramming upon IFNg stimulation. In order to overcome the plasticity of macrophages and stabilize their phenotype, for direct application of this cocktail on implants and in tissue engineering in vivo, the loading and release of the cytokine cocktail from a self-standing, transferable gelatin/tyraminated hyaluronic acid based release system was developed. The cytokine cocktail demonstrated its anti-inflammatory activity in controlled-release conditions. Our data suggest that the direct application of a potent M2-inducing cytokine cocktail in a transferable release system can be used as a personalized solution to improve the long-term acceptance of implanted materials.

Funding: EU FP7 for research and technological development and demonstration (Grant number 606294, IMMODGEL).

**References**

1. Kzhyshkowska J, Gudima A, Riabov V, Dollinger C, Lavalle P, Vrana NE. Macrophage responses to implants: prospects for personalized medicine. J Leukoc Biol. 2015 Dec;98(6):953-62. doi:10.1189/jlb.5VMR0415-166R.
2. Riabov V, Salazar F, Htwe SS, Gudima A, Schmuttermaier C, Barthes J, Knopf-Marques H, Klüter H, Ghaemmaghami AM, Vrana NE. Kzhyshkowska J. Generation of anti-inflammatory macrophages for implants and regenerative medicine using self-standing release systems with a phenotype-fixing cytokine cocktail formulation. Acta Biomater. 2017 Apr 15;53:389-398. doi:10.1016/j.actbio.2017.01.071.

**Mini-encyclopaedia of wound healing: lessons for predictive, preventive and personalised medicine**

Lara Stolzenburg-Veeser^1^ and Olga Golubnitschaja*^2,3,4^

^1^ Polytechnic University of Madrid, Spain

^2^Radiological Clinic, Rheinische Friedrich-Wilhelms-Universität Bonn, Sigmund-Freud-Str 25, 53105 Bonn, Germany

^3^Breast Cancer Research Centre, Rheinische Friedrich-Wilhelms-Universität Bonn, Bonn, Germany

^4^Centre for Integrated Oncology, Cologne-Bonn, Rheinische Friedrich-Wilhelms-Universität Bonn, Bonn, Germany

*Correspondence: Prof. Dr. Olga Golubnitschaja, Radiological Clinic, Rheinische Friedrich-Wilhelms-Universität Bonn, Sigmund-Freud-Str 25, 53105 Bonn, Germany; e.mail: Olga.Golubnitschaja@ukbonn.de

**Keywords**: Predictive preventive personalised medicine, Multi-level diagnostics, Biomarker panel, laboratory medicine, Surgery, Aesthetic medicine, Psychology, Gerontology, Diabetology, Endocrinology, Oncology, Cardiovascular disease, Radiology, Healthcare economy

**Abstract**

Main phases of the physiologic wound healing (WH) are the “early wound” (hemostasis characterised by fibrin clot formation, bleeding stoppage and creation of the hypoxic microenvironment) followed by the inflammatory phase well controlled by the wound healing mechanisms. That proceeds into the “late wound” featuring the proliferative phase comprising stem cell recruitment, angiogenesis/neovascularisation and wound contraction, followed by tissue remodelling and scar formation, finalising the physiologic WH [1].

Major players in the physiologic WH are the cellular stakeholders operating the repair processes, namely platelets, DECTs, neutrophils, macrophages, T lymphocytes, fibroblasts, myofibroblasts, keratinocytes, epithelial cells, endothelial cells, endothelial progenitor cells, stem cells, peripheral neurons, and extracellular matrix [2].

Potential biomarker panels includes growth factors (PDFGF, VEGF, TGF-ß, FGF protein core, EGF, IGF), cytokines/chemokines and transcription factors (IL-1, IL-6, TNF-α, IL-10, IL-4, IL-13, SDF-1α, HIF-1), neuropeptides (substance P, NGF, NPY, CGRP), matrix-proteases/inhibitors (MMPs/TIMPs), hormones and nutrients, amongst others [3, 4].

Impaired WH leads to post-surgical complications frequently observed in the elderly, chronic ulcers in diabetic patients, hindered and ineffective pain management, etc. However, these well-acknowledged examples are just the “tip of the iceberg”. The entire spectrum of potential consequences of the impaired WH is much mor broader, further extended by the chronic inflammation and cell/tissue transformation processes causing tissue dysfunction and calcification as well as (neuro)degeneration, progressive formation of pre/cancerous lesions and pre-metastatic niches. That is a subject for predictive diagnostics, targeted prevention and personalised medical approaches [5].

**References**

1. Guo S, Dipietro LA. Factors affecting wound healing. J Dent Res. 2010;89(3):219-29. doi:10.1177/0022034509359125.
2. Velnar T, Bailey T, Smrkolj V. The wound healing process: an overview of the cellular and molecular mechanisms. J Int Med Res. 2009;37(5):1528-42.
3. Werner S, Grose R. Regulation of wound healing by growth factors and cytokines. Physiol Rev. 2003;83(3):835-70.
4. Lara Stolzenburg-Veeser L, Golubnitschaja O. Mini-encyclopaedia of the wound healing repertoire for multiprofessional considerations. Journal of Proteomics, 2017. 0.1016/j.jprot.2017.07.017.
5. Avishai E, Yeghiazaryan K, Golubnitschaja O. Impaired wound healing: facts and hypotheses for multi-professional considerations in predictive, preventive and personalised medicine. EPMA J. 2017;8(1), 23-33. doi:10.1007/s13167-017-0081-y.

**Patient stratification, prediction, prevention and prognosis in wound healing: dream or reality?**

Eden Avishai^1^ and Olga Golubnitschaja^2,3,4^

^1^The Ruth and Bruce Rappaport Faculty of Medicine, Technion-Israel Institute of Technology, Haifa, 31096, Israel

^2^Radiological Clinic, Rheinische Friedrich-Wilhelms-Universität Bonn, Sigmund-Freud-Str 25, 53105 Bonn, Germany

^3^Breast Cancer Research Centre, Rheinische Friedrich-Wilhelms-Universität Bonn, Bonn, Germany

^4^Centre for Integrated Oncology, Cologne-Bonn, Rheinische Friedrich-Wilhelms-Universität Bonn, Bonn, Germany

*Correspondence: Prof. Dr. Olga Golubnitschaja, Radiological Clinic, Rheinische Friedrich-Wilhelms-Universität Bonn, Sigmund-Freud-Str 25, 53105 Bonn, Germany; e.mail: Olga.Golubnitschaja@ukbonn.de

**Keywords**: Predictive preventive personalised medicine, Systems medicine, Individualised patient profile, Multi-level diagnostics, Biomarker panel, Wound, Healing, Cardiovascular disease, Diabetes, Cancer, Health policy

**Abstract**

Physiologic wound healing (WH) is a well-controlled, highly orchestrated process involved in numerous ordinary (sporadic finger cut during meal preparation, regular manicure, etc.) and extraordinary (e.g. acute or planned surgery) conditions. WH is life-important. Physiologic WH is initiated by tissue injury and resolved within a reasonable time frame by the restoration of tissue integrity. The entire process follows the essential sequence of attributed phases: hemostasis, inflammation, proliferation, and tissue remodelling (Fig. 1) [1]. In contrast, impaired WH is characterised by low quality of phase-performance, stagnation (such as chronic inflammation), prolonged WH process or even non-healing wounds [2]. The quality of WH is highly individual, resulting from both non-modifiable (genetic predisposition, age) and modifiable (e.g. nutrition, lifestyle, preventable collateral pathologies such as type 2 diabetes) risk factors. Contextually, the quality of WH is considered n essential parameter in individualised patient profiling and the subject for advanced approaches by systems medicine, predictive diagnostics, targeted preventive measures, and treatments tailored to the person – altogether innovative approaches by predictive, preventive and personalised medicine as the “medicine of the future”. For that, multi-level diagnostics utilising comprehensive biomarker panels is proposed. The potential application is multifaceted: early diagnosis of related pathologies and disease predisposition, effective prevention and treatment of collateral pathologies, prediction and prognosis of post-surgical complications, improved individual outcomes in plastic surgery, amongst others [3].

**References**

1. Mathieu D, Linke JC, Wattel F. Non-healing wounds. In: Mathieu D, editor. Handbook on hyperbaric medicine. Dordrecht: Springer Netherlands; 2006. p. 401–28.
2. Demidova-Rice TN, Hamblin MR, Herman IM. Acute and impaired wound healing: pathophysiology and current methods for drug delivery, part 1: normal and chronic wounds: biology, causes, and approaches to care. Adv Skin Wound Care. 2012;25(7):304–14.
3. Avishai E, Yeghiazaryan K, Golubnitschaja O. Impaired wound healing: facts and hypotheses for multi-professional considerations in predictive, preventive and personalised medicine. EPMA J. 2017;8(1), 23-33. doi:10.1007/s13167-017-0081-y

**Fig. 1** The process of WH is characterised by an essential sequence of attributed phases; however, the quality of WH is highly individual
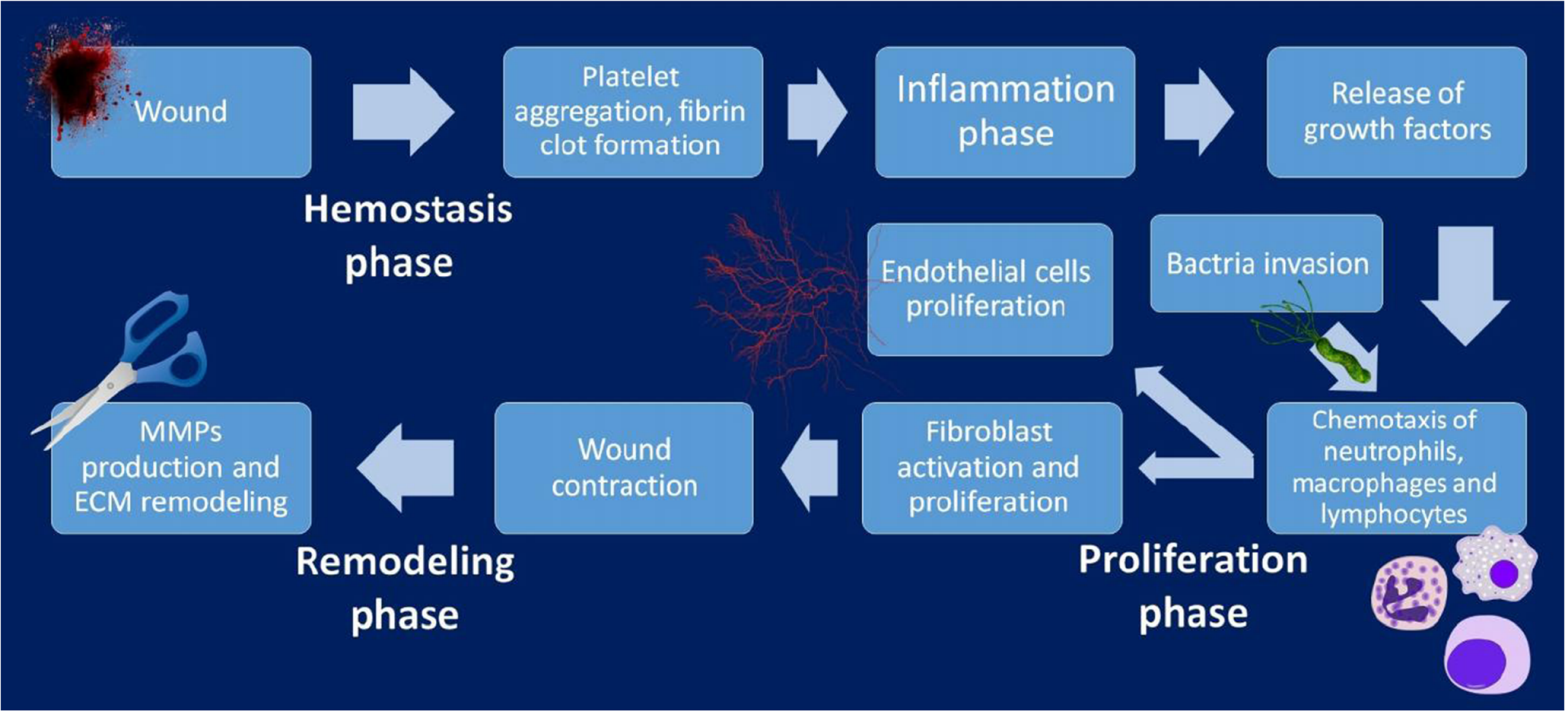


**Human saliva as a powerful source of information: multi-omics biomarker panels**

Dana Mileguir^1^ and Olga Golubnitschaja^2,3,4^

Institute of Dental Sciences, Faculty of Dental Medicine, The Hebrew University of Jerusalem, Israel.

^1^Faculty of Dental Medicine, Hadassah School of Dental Medicine, The Hebrew University of Jerusalem, 91120, Israel

^2^Radiological Clinic, Rheinische Friedrich-Wilhelms-Universität Bonn, Sigmund-Freud-Str 25, 53105 Bonn, Germany

^3^Breast Cancer Research Centre, Rheinische Friedrich-Wilhelms-Universität Bonn, Bonn, Germany

^4^Centre for Integrated Oncology, Cologne-Bonn, Rheinische Friedrich-Wilhelms-Universität Bonn, Bonn, Germany

*Correspondence: Prof. Dr. Olga Golubnitschaja, Radiological Clinic, Rheinische Friedrich-Wilhelms-Universität Bonn, Sigmund-Freud-Str 25, 53105 Bonn, Germany; e.mail: Olga.Golubnitschaja@ukbonn.de

**Keywords**: Predictive preventive personalised medicine, Systems medicine, Individualised patient profile, Multi-level diagnostics, Biomarker panel, Whole saliva, Multi-omics

**Abstract**

Multi-omics of saliva is garnering exponentially increasing attention as a preferred non-invasive diagnostic tool considered for a spectrum of health conditions, with strong potential for clinical utility. However, the association between specific patient profile and saliva components (omics signature) should be well understood [1]. Due to the complexity of its components deriving from different sources, whole saliva is particularly attractive for the biomarker set-up, reflecting systematic diseases such as cancer and metabolic syndrome [2,3]. The use of saliva for early and predictive diagnostics demonstrates many advantages, such as simple collection, which can be easily performed by non-specialised medical units or even by patients at home. Saliva collection is a non-invasive approach, in contrast to drawing blood or biopsies; it is inexpensive and presents no potential health adverse effects typical for any invasive sampling and/or radiological approaches utilised for molecular profiling such as magnetic resonance spectroscopy [4].

Multi-omics of saliva is a diagnostic tool based on the proteome, transcriptome, micro-RNA, metabolome, and microbiome, which is utilised in many biomedical fields. Recent advances have broadened the salivary diagnostic approach from the oral cavity to the whole physiological system, and thus point towards a promising future of salivary diagnostics for personalized individual medicine applications, including clinical decisions and post-treatment outcome prediction [5].

Biomarker panels created for specific diagnostic purposes include markers of periodontitis (SNPs), smoking (NGAL, CF058), oral carcinomas (TSCC), orthodontically induced inflammatory root resorption (DMP1, DSP, DPP), pancreatic cancer (KRAS, MBD3L2, ACRV, DPM1), and gastric cancer (CSTB, CSTB, CSTB) [1-5].

**References**

1. Cafiero C, Matarasso S. Predictive, preventive, personalised and participatory periodontology: 'the 5Ps age' has already started. EPMA J. 2013;4(1). doi:10.1186/1878-5085-4-16.
2. Murr A, Pink C, Hammer E, Michalik S, Dhople VM, Holtfreter B, et al. Cross-Sectional Association of Salivary Proteins with Age, Sex, Body Mass Index, Smoking, and Education. Journal of Proteome Research. 2017;(5)18. doi:10.1021/acs.jproteome.7b00133
3. Salazar MG, Jehmlich N, Murr A, Dhople VM, Holtfreter B, Hammer E, Volker U, Kocher T. Identification of periodontitis associated changes in the proteome of whole human saliva by mass spectrometric analysis. J. Clin. Periodontol. 2013;6(23), 40, 825 – 832. doi:10.1111/jcpe.12130
4. Kaczor-Urbanowicz KE, Deutsch O, Zaks B, Krief G, Chaushu S, Palmon A. Identification of salivary protein biomarkers for orthodontically induced inflammatory root resorption. Proteomics Clin Appl. 2017;(3)29. doi:10.1002/prca.201600119
5. Duz MB, Karatas OF, Guzel E, et al. Identification of miR-139-5p as a saliva biomarker for tongue squamous cell carcinoma: a pilot study. Cell Oncol. 2016; (39)187. doi:10.1007/s13402-015-0259-z

**New aspects of preventive endoscopic hemostasis in the treatment of peptic ulcer bleeding in the experimental condition**

E.F. Cherednikov*, S.V. Barannikov, M.N. Romantsov, A.V. Popov

Voronezh N.N. Burdenko State Medical University, Voronezh, Russia

The Department of Faculty Surgery

*Correspondence: Cherednikov E.F, Voronezh N.N. Burdenko State Medical University, Voronezh, Russia, Avenue of Revolution 14, 394036 Voronezh, Russia; e-mail: micvsma@yandex.ru

**Keywords**: Preventive treatment, Endoscopic hemostasis, Gastroduodenal bleeding

**Abstract**

The problem of endoscopic hemostasis merits a special place in the treatment of gastroduodenal bleeding [1].

We conducted a pilot study in 27 dogs, in which we caused two bleeding stomach ulcers in each animal (one was pilot, the other was control). In 15 pilot ulcers, hemostasis was performed with Asepticob-D insufflations in combination with Zhelplastan. In 12 pilot ulcers the bleeding was stopped with Asepticob-A and platelet-rich plasma [2]. Endoscopic treatment of the control ulcers was not exposed. The results of the study were evaluated according to the following criteria: hemostasis time, re-hemorrhages, defects healing time.

The experiments showed that in the pilot ulcers, hemostasis occurred in 3.92 ± 0.32 s (*p*<0.01). However, there were no re-hemorrhages. In the control ulcers, spontaneous bleeding occurred in 27.46 ± 1.12 s (*p*<0.01), and there were four episodes of re-hemorrhage. The pilot ulcers were healed in 8. 6± 0.18 days (*p*<0.01), the controls in 15.50 ± 0.31days (*p*<0.01).

Conclusion: Granular sorbents in combination with hemostatic agents perform three functions: permanent hemostasis, re-hemorrhage prevention, reparative regeneration acceleration.

**References**

1. E. F. Cherednikov, A.A. Kunin, E. E. Cherednikov, N. S. Moiseeva. The Role of Etiopathogenetic Aspects in Prediction and Prevention of Discontinuous-Hemorrhagic (Mallory-Weiss) Syndrome // The EPMA Journal, 2016.

**A new opinion on gastroduodenal bleeding prevention in patients with somatic pathology**

E. F. Cherednikov, A.V. Budnevsky, A.V. Popov, K.O. Fursov

Voronezh N.N. Burdenko State Medical University, Voronezh, Russia

The Department of Faculty Surgery

*Correspondence: Cherednikov E.F, Voronezh N.N. Burdenko State Medical University, Voronezh, Russia, Avenue of Revolution 14, 394036 Voronezh, Russia; e-mail: micvsma@yandex.ru

**Keywords**: Preventive therapy, Gastroduodenal bleeding, Patients with therapeutic pathology

**Abstract**

The problem of symptomatic erosive or ulcerative gastroduodenal bleeding in patients with therapeutic pathology is very relevant now [1].

We developed a curative program for prevention of gastroduodenal bleeding in patients with therapeutic pathology. This program is based on a multidisciplinary approach, diagnostic algorithm improvement, early preclinical endoscopic diagnosis on the second to third day, and well-timed administration of innovative technologies in local preventive treatment of symptomatic gastroduodenal erosions and ulcers with biologically active granular sorbents. A total of 144 patients with somatic pathology were involved in the clinical study. The patients were treated in a multi-field inpatient department and had signs of acute erosions and gastroduodenal ulcers. Clinical observations showed that early diagnosis by therapeutic endoscopy helped to reveal acute gastroduodenal erosions and ulcers without bleeding in 70.7% of patients (*p*<0.05). The well-timed local treatment of acute gastroduodenal erosions and ulcers with the help of biologically active granular sorbents enabled the prevention of hemorrhagic complications and extra operations, with threefold lower mortality.

**Reference**

1. E.F. Cherednikov, A.A. Kunin, E. E. Cherednikov, N.S. Moiseeva. The Role of Etiopathogenetic Aspects in Prediction and Prevention of Discontinuous-Hemorrhagic (Mallory-Weiss) Syndrome. EPMA J, 2016.

**Application of thermal imaging for personalized diagnosis of lipodystrophy**

Joanna Bauer^1^*, Ewelina Dereń^1^, Agnieszka Migasiewicz^2^, Halina Podbielska^**1**^

^1^Department of Biomedical Engineering, Faculty of Fundamental Problems of Technology, Wrocław University of Science and Technology, Wrocław, Poland

^2^Department of Cosmetology, Faculty of Physiotherapy, Wrocław University School of Physical Education, Wrocław, Poland

*Correspondence: Prof. Dr. Halina Podbielska, Department of Biomedical Engineering, Faculty of Fundamental Problems of Technology, Wrocław University of Science and Technology, 50-370 Wrocław, Wybrzeże Wyspiańskiego 27, Poland E-mail: halina.podbielska@pwr.edu.pl

**Keywords:** Infrared thermography, Thermographic analysis, Skin imperfection, Cellulite

**Abstract**

Lipodystrophy or panniculopathy (edematous fibrosclerotic panniculopathy), often called cellulite, is a dimpling appearance of the skin that can lower quality of life in many individuals, especially the female populations. Reliable and objective diagnosis of the stage (degree) of panniculopathy is difficult, since methods such as the Nürnberger-Müller or Elson scales are not very accurate and are subjective. The more advanced techniques such as high-frequency ultrasonography, ultrasound elastography or photometric measurements are also not well adapted for diagnosing this skin condition.

We propose exploiting thermal imaging for cellulite diagnosis and monitoring of anti-cellulite therapy. The superficial skin temperature is influenced by many factors, among them changes in the microcirculation, as well as uneven fat cell and water distribution.

The study was performed using a database of 100 thermal images of female volunteers, aged 19–22, with different stages of cellulite, first diagnosed by the Nürnberger-Müller scale. The study was conducted with the approval of the Senate Ethics Committee for Scientific Research at the University School of Physical Education in Wrocław. All recommendations of the European Association of Thermology for thermographic measurements in medical applications, including preparation of the attendees, exclusion criteria, environmental conditions and imaging system operational requirements, were strictly followed. A thermal image of the back thigh of each volunteer was recorded using a FLIR T335 thermographic camera.Next, all thermal images were analyzed using software specially developed by our group, called ThermaAnalyzer 2.0. This software allows one to determine the number and total area of skin irregularities, as well as the distribution in the examined region of interest (ROI). Asymmetrical and inhomogeneous superficial temperature distribution, seen as spots of different sizes and shapes on the thermal image, was observed in persons suffering from cellulite. Using quantitative parameters including total area of the blemishes and the extent of the area of irregularities on the thermal image enabled diagnosis of the cellulite stage with more than 80% accuracy. Thus, computer-aided thermal imaging analysis provides an objective method of diagnosis and monitoring of therapy results.

**References**

1. Goldman MP, Hexsel D. Cellulite: pathophysiology and treatment. 2nd ed. New York: CRC Press; 2010,
2. Hexsel D, Weber MB, Taborda ML, Dal'Forno T, Zechmeister-Prado D. A quality of life measurement for patients with cellulite. Surg Cosmet Dermatol. 2011;3(2):96–101.
3. Nürnberger F, Müller G. So-called cellulite: An invented disease. J Dermatol Surg Oncol. 1978;4:221-29.
4. Migasiewicz A, Pelleter M, Bauer J, Dereń E, Podbielska H. Influence of the skin imperfection on the personal quality of life and possible tools for objective diagnosis. EPMA J, 2016; 7(1):23-4.
5. European Association of Thermology, 2016 http://www.europeanthermology.com/cms32/index.php?GUIDELINES. Accessed 4 Oct 2016.

**Personalized telecare in pregnancy by means of remote cardiotocography**

Anna Skotny^1^, Halina Podbielska^2^* ^1^ Nestmedic S.A, Wrocław

^2^Department of Biomedical Engineering, Faculty of Fundamental Problems of Technology, Wrocław University of Science and Technology, Wrocław, Poland

*Correspondence: Prof. Dr. Halina Podbielska, Department of Biomedical Engineering, Faculty of Fundamental Problems of Technology, Wrocław University of Science and Technology, 50-370 Wrocław, Wybrzeże Wyspiańskiego 27, Poland E-mail: halina.podbielska@pwr.edu.pl

**Keywords:** Pregnancy, Cardiotocography, Telemonitoring

**Abstract**

Cardiotocography (CTG) is used during pregnancy to monitor fetal well-being. It enables early detection of fetal distress by monitoring the fetal heartbeat along with uterine contractions. More advanced systems record fetal movements as well. Diagnosis by means of CTG is usually performed only in the third trimester, mainly in stationary health care centers (hospitals, ambulatory units or well-equipped private practices). Thus, pregnant women are required to travel to the corresponding location, and wait for the test and then the diagnosis.

In the case of high-risk pregnancy, CTG is a very useful diagnostics tool. An abnormal CTG indicates serious abnormalities, and in late pregnancy may lead to emergency caesarian section. However, even in low-risk pregnancy, it gives the comfort of the proper fetal care what undoubtedly influences a well-being of a future mother. There are some attempts to allow the in situ CTG diagnosis without the necessity to visit health care unit.

Here, we report on an innovative integrated device based on a microprocessor and a dedicated operating system for in-home use by pregnant women. GSM/GPRS/3G-based communication enables a territorially almost unlimited CTG signal transfer from the woman to the telecare center.

The pregnant woman is instructed by the doctor or nurse on how to use the device and how to transmit the recordings. She then receives the recorded signals in the form of diagrams, along with explanations relating to the fetal heart rate, fetal movements, mother's HR, and warning of (if present) decelerations, signal variability, etc. She is also instructed what to do, and in the case of emergency, she can be transferred to the nearest hospital as quickly as possible.

**References**

1. Daly N, Brennan D, Foley M, O'Herlihy C., Cardiotocography as a predictor of fetal outcome in women presenting with reduced fetal movement, Eur J Obstet Gynecol Reprod Biol. 2011;159(1):57-61. doi:10.1016/j.ejogrb.2011.07.002. Epub 2011 Sep 6.
2. Fanelli A, Signorini MG, Ferrario M, Perego P, Piccini L, Andreoni G, Magenes G., Telefetalcare: a first prototype of a wearable fetal electrocardiograph. Conf Proc IEEE Eng Med Biol Soc. 2011;2011:6899-902. doi:10.1109/IEMBS.2011.6091737.
3. Thellesen L, Sorensen JL, Hedegaard M, Rosthoej S, Colov NP, Andersen KS, Bergholt T. Cardiotocography interpretation skills and the association with size of maternity unit, years of obstetric work experience and healthcare professional background: a national cross-sectional study. Acta Obstet Gynecol Scand. 2017. doi:10.1111/aogs.13171.

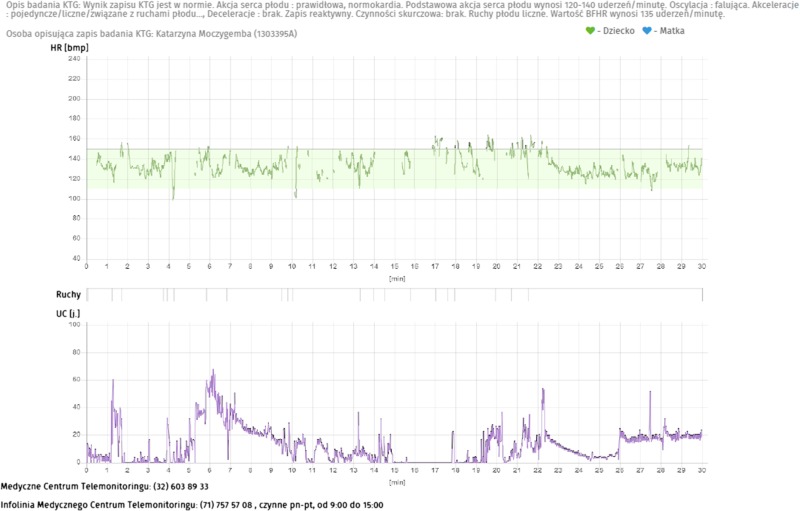


**Fig. 1** Exemplary CTG diagrams received by pregnant women after sending the recorded signals to the telemonitoring center

**PPPM IN DENTISTRY**

**Stem cells and tooth regeneration: an overview**

Mahmood S. Mozaffari, Department of Oral Biology/Pharmacology & Toxicology, Dental College of Medicine, Augusta University, Augusta, Georgia 30912-1128

*Correspondence: Dr. Mahmood S. Mozaffari, 1120 15^th^ Street; CL-2134, Augusta University, Augusta, Georgia 30912-1128. E-mail: Mmozaffa@augusta.edu.

**Keywords:** Predictive preventive personalized medicine, Personalized dentistry, Tooth regeneration, Stem cells

**Abstract**

Clinical dentistry continues to rely heavily on traditional restorative approaches and biomaterials to treat/replace damaged and diseased teeth and the periodontium. More recently, implant dentistry has also made major strides in techniques for replacement of missing teeth. Importantly, the recognition of the pivotal role of stem cells in tissue repair and regeneration has led to intense research to identify tooth-derived stem cells. Studies have subsequently shown not only the ability of tooth-derived stem cells to generate various components of the tooth and periodontal structures, but also their utility in regenerative medicine, as exemplified by the generation of cells of organs such as the heart, liver and brain. More recent studies have demonstrated (functional) tooth regeneration in animal models that display physical properties seemingly similar to those of natural teeth. Even more exciting is the advent of induced pluripotent stem cells (iPSCs) which, similar to embryonic stem cells, have the hallmark feature of differentiation to cells of the three germ layers; iPSCs make it feasible to generate epithelial and mesenchymal stem cells for eventual in vitro generation of a tooth bud, followed by implantation into the tooth socket. Since the creation of iPSCs requires the use of somatic cells, investigators have explored the possibility of using integration-free human urine-induced pluripotent stem cells for tooth regeneration. Clearly, this approach has the potential to ultimately lead to “custom-making” of tooth and supporting structures, thereby bringing true meaning to the term “personalized dentistry”. Other highly relevant research areas involve the identification of growth factors and delivery options as well as scaffolding material that would be most conducive to tooth regeneration. These aspects and their relevance to PPPM are the focus of this presentation.

**Comparison of dental fear and anxiety in relation to dental health in Estonian and Vietnamese children**

Jana Olak* ^2^; Minh Son Nguyen^1,2^; Thuy Trang Nguyen^1^; Bui Bao Tien Nguyen^1^; Mare Saag^2^

^1^ Danang University of Medical Technology and Pharmacy, Danang, Vietnam

^2^ Institute of Dentistry, University of Tartu, Tartu, Estonia

*Correspondence: Assoc. Prof. Jana Olak, University of Tartu, Raekoja plats 6, Tartu, Estonia; e-mail: Jana.Olak@ut.ee

**Keywords:** Schoolchildren, Dental anxiety, Dental fear, Dental caries

**Abstract**

**Objectives.** We hypothesized was that differences in culture and oral health care system in Estonia and Vietnam might have an impact on dental health (dmft+DMFT) and dental fear and anxiety (DFA) in children of the two countries. The aim of this study was to compare dental fear and anxiety (DFA) in relation to dental health between Estonian and Vietnamese schoolchildren using the modified Dental Subscale of the Children's Fear Survey Schedule (MCFSS-DS).

**Methods.** Among a total of 900 schoolchildren 8 to 10 years of age, 344 from Estonia and 556 from Vietnam agreed to participate in this study. Dental health was recorded based on dental caries experience index of mixed dentition (dmft+DMFT). DFA was measured using the MCFSS-DS with five Likert-scale response options (1 = not afraid at all, 5 = very afraid).

**Results.** The mean dmft+DMFT score in Estonian and Vietnamese schoolchildren was 5.2 ± 3.1 and 4.1 ± 3.2, respectively. The mean score of 11 fear items among Vietnamese children (20.8 ± 9.1) was significantly higher than that of Estonian children (15.4 ± 4.4). The prevalence of high DFA in Estonian schoolchildren (30.7%) was statistical equivalent to that of Vietnamese schoolchildren (28.0%, *p*>0.05).

**Conclusion.** There were significant differences in DFA between Estonian and Vietnamese schoolchildren. Differences in oral health care systems between the two countries might be considered as a factor affecting dental health and DFA among schoolchildren.

**References**

1. Olak J, Saag M, Honkala S, Nõmmela R, Runnel R, Honkala E, et al. Children’s dental fear in relation to dental health and parental dental fear. Stomatol Public Inst Odontol Stud Al. 2013; 15: 26-31
2. Eitner S, Wichmann M, Paulsen A, Holst S. Dental anxiety–an epidemiological study on its clinical correlation and effects on oral health. J Oral Rehabil. 2006; 33: 588-593
3. Rantavuori K, Lahti S, Hausen H, Seppä L, Hausen H. Dental fear of Finnish children in the light of different measures of dental fear. Acta Odontol Scand. 2005; 63: 239-244
4. Klingberg G, Broberg AG. Dental fear/anxiety and dental behaviour management problems in children and adolescents: a review of prevalence and concomitant psychological factors. Int J Paediatr Dent. 2007; 17: 391-406

**Age-specific attitudes regarding dental health**

Isaeva Elena^1^, Sitkina Eugenia^1^, Kudryavtseva Тatiana^1^, Loboda Ekaterina^1, 2^

^1^ First St. Petersburg State Medical University of the Ministry of Healthcare of Russian Federation

^2^ City Periodontology Centre "PAKS"

Isaeva E.R. Psych.D, professor, head of the Department of General and Clinical Psychology

Sitkina E.V., assistant of the Department of General and Clinical Psychology

Kudryavtseva T.V. MD, professor of Therapeutic Dentistry Department

Loboda E.S., Ph.D., associate professor of Therapeutic Dentistry Department

*Correspondence: Sitkina Eugenia, Russia, St. Petersburg, L'va Tolstogo st., 6/8, 197022; e.mail: sitkina_evgenya@mail.ru

**Keywords.** Compliance, Treatment adherence, Patient psychological peculiarities, Relevance to treatment, Dental care, Diseases of the oral cavity

**Abstract**

Relevance. The success of dental treatment depends both on the professionalism of the dentist and on the patient’s compliance with the doctor’s medical recommendations [1]. One of the important factors of compliance is the attitude of patients toward their own dental health [2,3].

Methods. The study involved 136 people aged 18 to 60 years. Methods of diagnosis: "Health Attitude" by R.A. Berezovskaya, Questionnaire on the relationship to dental health.

Results. Among respondents, 34% defined "dental health" as a good physical or mental health, and 16% as a lack of disease. Most patients assessed their dental health as an average of 63.5 points out of 100, while 75% of respondents indicated that they needed dental treatment. Respondents aged 30–39 years (67 points) rated their health the best. A total of 37% of respondents noted that they did not visit the dentist because of high prices for services, and 21% because they were afraid of dentists. Respondents noted that in health, they relied more on the opinions of doctors and less on the media. Among patients, 42% across different ages preferred the empathic, non-referential type of doctor. A doctor of this type listens to the patient, builds partnerships, and discusses treatment strategies with the patient. Most patients (65%) preferred a female dentist.

Findings. The majority of respondents noted that dental health was important and they needed treatment, but they postponed it. Patients preferred a doctor who discussed the course of treatment.

Conclusion. The study revealed the need to develop a plan for individual approaches for patients of different age groups.

**References**

1. Orekhova L.Y., Kudryavtseva T.V., Isaeva E.R., Tachalov V.V., Loboda E.S., Sitkina E.V. Interrelation of the peculiarities of individual oral care from psychological personal characteristics. Stomatology of Slavic states: a collection of works on the materials of the VIII International Scientific and Practical Conference edited by A.V. Tsimbalistova, B.V. Trifonova, A.A. Kopytova. - Belgorod: Publishing house "Belgorod" Research Institute "BelSU". 2015; 208-211.
2. Collins F. M.: Factoring Patient Compliance into Oral Care // Earn 2 CE credits. 2008, https://www.dentalacademyofce.com/courses/2059/PDF/1103_CEDfpc-compliance_rev1.pdf
3. Macri D.: The expert advice: Dental patient compliance hinges on effective communication strategies// RDH Magazine. 2016;36(6). http://www.rdhmag.com/articles/print/volume-36/issue-6/contents/the-expert-advice.html

**Patient-specific periodontology: reality and outlook**

Tachalov Vadim^1^, Orekhova Ludmila^1, 2^, Isaeva Еlena^1^, Loboda Еkaterina^1, 2^.

1. First St. Petersburg State Medical University of the Ministry of Healthcare of Russian Federation

2. City Periodontology Centre "PAKS"

Orekhova L.Yu. MD, professor, head of the Department of Therapeutic Dentistry Department

Isaeva E.R. Psych.D, professor, head of the Department of General and Clinical Psychology Tachalov V.V., Ph.D., associate professor of Therapeutic Dentistry Department

Loboda E.S., Ph.D., associate professor of Therapeutic Dentistry Department

*Correspondence: Tachalov Vadim, Russia, St. Petersburg, L'va Tolstogo st., 6/8, 197022; e.mail: tachalov@mail.ru

**Keywords.** Compliance, Treatment adherence, Patient psychological peculiarities, Relevance to treatment, Dental care, Diseases of the oral cavity

**Abstract** In this article, the psychological characteristics of dental patients with periodontal disease are summarized, along with a description of patients with positive and negative dynamics of treatment.

Objective: To identify relationships between the characteristics of a patient’s personality and their implementation of dentist recommendations.

Materials and methods: Forty-five patients were surveyed, with an average age of 43 years. The following psycho-diagnostic methods were used: 1) a multidimensional questionnaire on health; 2) the "Diagnosis of interpersonal relationships" test; 3) Questionnaire characterological Leonhard–Shmishek; 4) individual-typological questionnaire Sobchik L.N.; 5) questionnaire of self-evaluation status; and 6) Behterevsky Institute Personality Inventory. The following dental indexes of oral health were measured: 1) CPITN, 2) PMA, 3) Saxer–Muhlemann, 4) Silness–Loe, and 5) OHI-S.

Results: Based on the results of two measurements of the dental indexes, three groups of patients were identified: 1) those with good oral hygiene, 2) those with poor hygiene and positive dynamics (improving dental health index), and 3) those with poor hygiene and negative dynamics (deterioration indexes). A relationship was found between dentist recommendations and patient personal characteristics.

Summary: Most patients tried to follow the doctor's recommendations for oral care, as evidenced by the decline in dental health indices in the second survey. Differences in patient personal characteristics, whether patients did or did not follow recommendations, and gender differences were found between groups with improvement and deterioration indexes.

**References**

1. AyerWilliam, Psychology in Dental Practice. - St. Petersburg: Peter. 2008; 219. In Russian
2. Binhas E. Comment augmenter l`acceptation des plans de traitement. Dialgue. 1999; 13–15
3. Doctor-patient: communication and interaction. - Geneva - Amsterdam - Kiev: WHO.1996; 55. In Russian
4. Tachalov V., Orekhova L., Kudryavtseva T., Isaeva E., Loboda E. Manifestations of personal characteristics in individual oral care. EPMA J. 2016;7:8. doi: 10.1186/s13167-016-0058-2.

**Electromagnetic influence on microstructural changes in dental filling materials: improvement in physical and mechanical properties**

Natalia S. Moiseeva*, Anatoly A. Kunin, Ruslan A. Shabanov, Nakhid T. Aliev

Voronezh N.N. Burdenko State Medical University, Voronezh, Russia

The Department of Hospital Dentistry

*Correspondence: PhD., Natalia S. Moiseeva, The Department of Hospital Dentistry, Voronezh N.N. Burdenko State Medical University, Voronezh, Russia, Avenue of Revolution 14, 394036 Voronezh, Russia; e-mail: natazarova@yandex.ru

**Keywords:** Caries prevention, Electromagnetic field, Scanning electron microscopy, Physical and mechanical properties of filling material

**Abstract**

Nowadays, the science of dental materials is intently focused on the creation of "ideal" filling material. The research carried out at the Dentistry Department of Voronezh State Medical University was motivated by the need to improve the properties of filling materials under the influence of an electromagnetic field to enhance their physical and mechanical properties and to prevent dental complications.

It is well known that filling materials used for dental caries prevention and treatment consist of various components, including synthetic polymers, which play an important role in forming the main structure of these materials as well as in their physical and chemical properties.

Scanning electron microscopy (SEM) was used to study the properties of polymeric restorative materials before and after their exposure to the electromagnetic field in vivo, including a study of microstructural characteristics of filling materials.

The SEM results confirmed the significant changes in the microstructure of polymeric restorative materials under the influence of an electromagnetic field, i.e. an improvement in their mechanical properties.

Analysis of the data indicates the presence of microstructural changes in the physical and mechanical properties of filling materials after the electromagnetic field exposure, namely increased strength under compression, diametrical fracture and bending.

These findings may contribute to increasing the service life of filling materials and preventing the occurrence of secondary caries, thus positively affecting the quality of treatment on the whole.

**Improving the effectiveness of dental caries prevention using therapeutic toothpastes**

Anatoly A. Kunin, Natalia S. Moiseeva*, L.E. Mekhantieva

Voronezh N.N. Burdenko State Medical University, Voronezh, Russia

The Department of Hospital Dentistry

*Correspondence: PhD., Natalia S. Moiseeva, The Department of Hospital Dentistry, Voronezh N.N. Burdenko State Medical University, Voronezh, Russia, Avenue of Revolution 14, 394036 Voronezh, Russia; e-mail: natazarova@yandex.ru

**Keywords:** Caries prevention, Therapeutic toothpaste, Electromagnetic field

**Abstract**

A number of authors consider rational oral hygiene an integral component of etiopathogenetic prevention of dental caries and periodontal disease.

For in vitro studies and the evaluation of physical and chemical properties, the structure and substructure of 30 toothpaste samples were examined using a Libra 120 transmission electron microscope (ZEISS) with different techniques: fast electron diffraction, and bright-field and dark-field imaging. In vivo studies included clinical assessment of the state of hard dental tissues after the application of ROCS Sensitive Instant Relief toothpaste during the month before and after electromagnetic field exposure.

The in vitro results revealed certain changes in the microstructure of the ROCS Sensitive Instant Relief toothpaste after electromagnetic field exposure consisting of compound particle enlargement and corresponding changes in density.

According to the in vivo results, a reliable positive effect after the application of therapeutic toothpaste was achieved in 98% of cases. However, when it was exposed to the electromagnetic field, a greater anti-caries effect was observed, which was confirmed by numerous diagnostic techniques.

Thus, as a result of the application of ROCS Sensitive Instant Relief toothpaste in the study group, the mean value of the dental hygiene index was reduced from 0.80 ± 0.06 to 0.50 ± 0.02 towards an improvement in oral hygiene status. The cariogenicity of dental plaque disappeared in most patients of the study group, which was confirmed by the statistical data, from 40.4 ± 2.53% before to 13.73 ± 1.82% after. There was no significant reduction in the control group indices.

The studies thus revealed certain changes in the microstructure of ROCS Sensitive Instant Relief toothpaste caused by the electromagnetic field and in the ion-exchange processes in hard dental tissues during their use, which leads to a significant increase in their therapeutic and prophylactic efficacy.

**The effectiveness of a personal approach to learning manual skills during dental hard tissue preparation**

B.R. Shumilovich *, V.V. Rostovtsev, A.V. Saneev, L.M. Adunts

Voronezh N.N. Burdenko State Medical University, Voronezh, Russia

*Correspondence: Dr.Med.Sc., Shumilovich B.R., The Department of Hospital Dentistry, Voronezh N.N. Burdenko State Medical University, Voronezh, Russia, Avenue of Revolution 14, 394036 Voronezh, Russia; e-mail: bogdanshum@gmail.com

**Keywords:** Training module, Removable prosthesis, Mechanical preparation of hard tissues, Manual skills, Simulation

**Abstract** The development of practical skills using simulation training eliminates the risk to patient’s health and life, provides a personalized approach to learning and the possibility of developing practical skills and bringing them to automatism, and ensures the objective control of the quality of their execution.

**Objective:** to determine the effectiveness of a simulation method of teaching manual skills when handling removable dentures in a clinical setting.

**Methods:** The first phase of the study involved 82 third-year students, divided into a control group where the training was conducted with the use of standard phantoms, and a study group that used a fifth-generation CDS 100 dental simulator. The second phase included 48 young specialists who had participated in the first phase, and were divided into groups similar to those of the first phase. The quality of the preparation for the both stages was objectively assessed with a Zirkozahn S600 ARTI scanner.

**Results:** In the control group, the main reason for the need for additional correction of the abutment teeth at both stages was inadequacy of the dental ledge, with an ANOVA factor indicator of 1.1 for the first phase and of 1.15 for the second, which indicates statistical identity of predictors in this group. The study group found no statistically significant difference between the quality of the dental ledges executed at the first and second stages of the study, with an ANOVA factor indicator of 0.9, which indicates the statistical significance of such a predictor for preliminary acquisition of automatic movements acquired while working with the simulator.

**Conclusion:** The use of the CDS 100 interactive dental simulator provides a higher level of practical skills development and, most significantly, their "survival" compared with the classical form of education, which is confirmed by subsequent clinical practice.

**Prediction of the development of dental caries in adolescents based on the functional state of the masseter muscle**

Yury A. Ippolitov^1^, Mikhail E. Kovalenko^1^, Ekaterina N. Bondareva^1^, Natalia S. Moiseeva^2^ *

Voronezh N.N. Burdenko State Medical University, Russia

^1^The Department of Pedodontics Dentistry

^2^The Department of Hospital Dentistry

*Correspondence: PhD., Natalia S. Moiseeva, The Department of Hospital Dentistry, Voronezh N.N. Burdenko State Medical University, Voronezh, Russia, Avenue of Revolution 14, 394036 Voronezh, Russia; e-mail: natazarova@yandex.ru

**Keywords:** Caries prediction, Electromyography, Masseter and temporal muscles activity level

**Abstract**

The scientific data proving that enamel is a mineral and protein tissue with a system of microcirculation in the form of enamel tubules served as the basis for this study. The orifices of the enamel tubules open onto the enamel surface. The dental pulp fluid rushes out through enamel and dentinal canals because of the pressure gradient in the pulp. The chewing function is the only physiological mechanism to provide the pressure gradient.

The aim of the study was to evaluate the functional state of superficially localized masseter and temporal muscles in adolescents aged 13–16 years with high and medium caries resistance, by means of electromyography.

Electromyography was conducted to evaluate the masseter and temporal muscles in 62 adolescents aged 13–16. The assessment was carried out using the Synapsis apparatus. The degree of tooth caries development was determined by intraoral roentgen diagnostics using the de Götzen S.r.l. electrometric diagnostics dental apparatus and light-induced diagnostics.

The electrobiological activity of the masseter muscles was found to be significantly lower in patients with medium caries resistance than in adolescents with high caries resistance. There was an imbalance between the electrobiological activity indices in the masseter and temporal muscles; an increase in masseter fatigue in response to a static load in adolescents with medium caries resistance was observed. That situation may lead to a reduced pressure gradient of the dental pulp fluid in the pulp cavity, its failure to enter enamel tubules, and consequently to lower enamel resistance, with the risk of a cariogenic situation in adolescents.

**Dental caries prevention using the modulated red light (MRL)**

Svetlana N. Pankova, Olga A. Kumirova, Natalia S. Moiseeva*, Olga A. Azarova

Voronezh N.N. Burdenko State Medical University, Voronezh, Russia

The Department of Hospital Dentistry

*Correspondence: PhD., Natalia S. Moiseeva, The Department of Hospital Dentistry, Voronezh N.N. Burdenko State Medical University, Voronezh, Russia, Avenue of Revolution 14, 394036 Voronezh, Russia; e-mail: natazarova@yandex.ru

**Keywords:** Preventive dentistry, MRL, Anticaries effect

**Abstract**

Objective. According to a number of authors, low-intensity laser radiation contributes to a significant increase in the resistance of enamel to caries. The development of new methods of prevention with the use of physiotherapy, and the resulting decrease in the volume of medical measures and material costs, is highly relevant. The main purpose of this study is to assess the effectiveness of preventive measures to achieve an anticaries effect using MRL physiotherapy with the Svetozar device.

**Materials and methods.** The Svetozar laser and photomedicine device (wavelength, 628 nm; radiation width, 10 nm; pulse frequency modulation, 76 Hz; wide interval pulses; radiation power, 6 mW) was used. Caries preventive measures were carried out in 30 patients aged 18 years. The teeth were dried and covered with fluoride varnish, and were then subjected to irradiation with the Svetozar for 2 min per tooth. The preventive measures were performed within 5 days, and the course was repeated in 6 months. Efficiency monitoring was carried out before and after treatment using acidic enamel biopsy according to V.K. Leontiev and the tooth plaque cariogenicity (TPC) evaluation.

**Results.** Preventive therapy resulted in a 43% reduction in the rate of acid solubility of enamel and a 54.8% decrease if combined with fluoride varnish. The use of MRL resulted in 82.5% complete elimination of TPC.

**Conclusion.** MRL plays an important role in caries prevention in adults, as it has a pronounced anti-caries effect on tooth enamel. If it is combined with fluoride varnish, it is possible to achieve a 71.5% reduction in the growth of dental caries.

**Prevention of cross infections in dental care facilities in different environments**

Svetlana N. Pankova*, Olga A. Azarova, Svetlana G. Shelkovnikova, E.A. Azarova

Voronezh N.N. Burdenko State Medical University, Russia

*Correspondence: PhD., Svetlana N. Pankova, The Department of Hospital Dentistry, Voronezh N.N. Burdenko State Medical University, Voronezh, Russia, Avenue of Revolution 14, 394036 Voronezh, Russia; e-mail: micvsma@yandex.ru

**Keywords:** Prevention, cross infection, Bacterial pollution of air, Dental care

**Abstract**

Bacterial pollution of air is associated with the existence of proinflammatory infections in surgical patients and the use of dental equipment dispersing bacterial aerosols of the oral cavity during orthopedic and therapeutic treatment.

**Objective:** Scientific justification of the principles of cross infection prevention in dental care facilities.

**Materials and methods:** We studied the material obtained in bacteriological studies of 2160 air samples taken in dental rooms of different profiles, in winter and in summer, in the city and in the countryside.

**Results:** The beginning of a working day is characterized by different levels of bacterial pollution of air in dental rooms of different profiles (surgical – 779 CFU/m^3^, therapeutic – 1702 CFU/m^3^, orthopedic – 1942 CFU/m^3^). In the middle of a working day, bacterial pollution increases (surgical – 2075 CFU/m^3^, therapeutic and orthopedic – 2525 CFU/m^3^). At the end of a working day, the bacterial pollution rate is the highest (surgical – 2623 CFU/m^3^, therapeutic – 3701 CFU/m^3^, orthopedic – 3709 CFU/m^3^).

Bacterial pollution in dental rooms differs between summer and winter. At the beginning of a working day, the summer indices are higher than those of winter (winter – 1702 CFU/m^3^, summer – 2188 CFU/m^3^). In the middle of a working day, the summer indices are also higher (winter – 2525 CFU/m^3^, summer – 2963 CFU/m^3^). At the end of a working day, however, the winter indices are higher (winter – 3701 CFU/m^3^, summer – 3098 CFU/m^3^).

Bacterial pollution in dental rooms differs between the city and the countryside: at the beginning of a working day (city – 1702 CFU/m^3^, countryside – 2370 CFU/m^3^); in the middle of a working day (city – 2525 CFU/m^3^, countryside – 3366 CFU/m^3^); at the end of a working day (city – 3701 CFU/m^3^, countryside – 3827 CFU/m^3^).

**Fundamental strategy and individual prevention of inflammatory periodontal diseases**

Olga I. Oleinik*, Andrey V. Sushchenko, Elena A. Alferova, Oksana P. Krasnikova

Voronezh N.N. Burdenko State Medical University, Russia

*Correspondence: Dr.Med.Sc., Olga I. Oleinik, The Department of Hospital Dentistry, Voronezh N.N. Burdenko State Medical University, Voronezh, Russia, Avenue of Revolution 14, 394036 Voronezh, Russia; e-mail: micvsma@yandex.ru

**Keywords**: Inflammatory periodontal diseases, Individual preventive measures, Regular health examinations, Medical and preventive activities

**Abstract**

This article covers the necessity of periodontal disease prevention at the individual level and development of new forms of periodontal care.

**Objective**. To develop organizational and methodological principles for performing individual medical and preventive activities for periodontal disease as part of regular health examinations.

**Materials and methods**. A total of 150 patients in group 1 (common preventive measures) and 150 patients in group 2 (individual approach) aged 19–45 were examined. Various methods were used to specify the extent and content of interventions, which included medical history, scanning electron microscopy (SEM), and electron microprobe analysis (EMPA).

**Results.** Periodontal disease prevention was performed in accordance with the three-stage principle: selection, recording, monitoring.

Significant changes in oral hygiene status were registered in patients of group 2 in the 3 years after initiation of the program. Among patients, 16.7% needed periodontal disease treatment, which was 4.2-fold lower than before the preventive programs were implemented. The reduction in clinical indices was greater than 85%. These findings indicated increased resistance of tooth enamel to caries. No significant changes were observed in the oral hygiene status in patients of group 1 for the duration of the observation period.

**Conclusion**. Based on the results, we can conclude that medical and preventive activities based on dynamic observation contribute to a significant decrease in the incidence and intensity of periodontal pathology in certain groups of patients, as well as improved teeth and oral mucosa health.

**Personalized approach for assessing the tonus of tongue muscles in patients with ankyloglossia**

Dmitry Yu. Kharitonov^1^, R.V. Lesnikov^1^, Elena Yu. Zolotareva^1^, Natalia S. Moiseeva^2^ *

Voronezh N.N. Burdenko State Medical University, Russia, Voronezh

^1^Department of oral and maxillofacial surgery

^2^ Department of Hospital Dentistry

*Correspondence: PhD., Natalia S. Moiseeva, The Department of Hospital Dentistry, Voronezh N.N. Burdenko State Medical University, Voronezh, Russia, Avenue of Revolution 14, 394036 Voronezh, Russia; e-mail: natazarova@yandex.ru

**Keywords:** Personalized approach, Ankyloglossia, Tongue strength, Myotherapy

**Abstract**

The functional pattern of the tongue neuromuscular apparatus and its morphological characteristics plays an important role in the development of the majority of malocclusion disorders. Based on an analysis of the scientific literature, there is no unified position regarding the research methodology and the treatment protocol for ankyloglossia combined with malocclusion. In the present study, the strength parameters of tongue muscles were evaluated in patients with ankyloglossia before and during the course of treatment. The research program involved a group of patients with ankyloglossia which included 39 children age 7 to 9 years. The control group included 20 children of similar age. In the patients of the base group, motivational-oriented activities such as frenuloplasty, orthodontic therapy, and instrumental myotherapy were conducted. The strength parameters of the tongue were evaluated using the IOPI Medical system. In the base group, a significant reduction in the strength of the tongue elevation muscles was observed.

With closed mouth, the force parameters were reduced by 27.3%, and with open mouth by 59.16%, in comparison with the results of the control group (*р*<0.05). Functional therapy resulted in an increase in the strength activity of the tongue as well as its endurance indicators. Thus, the implementation of frenulotomy combined with subsequent orthodontic therapy and myotherapy leads to an increase in the strength parameters of the tongue muscles. The use of a technology to increase children’s motivation for treatment is a determinant of the enhancement of tongue muscle endurance and the active remodeling of the tongue’s functional pattern.

**Orthopedic treatment of patients suffering from chronic oral mucosal diseases with removable prosthesis structures on a background of immunological correction**

A.L. Solovyeva^1^ *, E.A. Leshcheva^2^, N.G. Mashkova^2^, O.A. Kumirova^1^

Voronezh N.N. Burdenko State Medical University, Voronezh, Russia

^1^Department of Hospital Dentistry

^2^Department of Faculty Dentistry

*Correspondence: PhD., A.L. Solovyeva, The Department of Hospital Dentistry, Voronezh N.N. Burdenko State Medical University, Voronezh, Russia, Avenue of Revolution 14, 394036 Voronezh, Russia; e-mail: micvsma@yandex.ru

**Keywords:** Personalized approach, Prosthetics, Immunological correction, Removable structures, Prevention of diseases of the oral cavity mucosa

**Abstract**

A sequence of therapeutic and diagnostic stages of orthopedic treatment and prophylaxis in patients with chronic oral mucosal diseases based on the complex application of the soft base material Mollosil plus and Hepon immunomodulating preparation has been developed and described. Forty patients were divided into groups. The patients underwent a complete dental examination, X-ray studies, bacteriological examination, bacterioscopy and immunological examination of saliva. The assessment of the condition of the mucosa was carried out on days 3 and 10.

During the objective assessment of prosthetic bed mucosa, it was revealed that the greatest number of patients with diffuse hyperemia had occurred on the third day. When studying the nature of changes in the mucous membrane of the prosthetic bed, along the integrity disturbance of the epithelial cover, on the third day after the prosthetics, we registered a significantly lower amount of traumatic erosion in 50% of patients who received an immunomodulating therapy. In patients of this group, the content of s-IgA in saliva increased significantly throughout the period of observation, and the IgA and IgG values ​​after a certain increase on the tenth day subsequently returned to the baseline level (0.125 ± 0.005 М ± m). The reduction in local humoral immunity intensity in patients taking Hepon was accompanied by the activation of cellular immunity, which was evidenced by increased phagocytic number (5.0 ± 0.3) and phagocytic index values (42.0 ± 1.2). Throughout the period of adaptation to the prostheses, 80% of patients expressed positive dynamics in the reduction of the area of inflammation by 20% (2.5-fold).

The effectiveness of treatment in patients with chronic oral mucosal diseases was proved by the removable orthopedic prosthesis design on the basis of clinical and laboratory studies of the complex application of elastic base material and immunocorrection.

**Dentaseptin for periodontal diseases prevention**

A.N. Morozov*, N.V. Chirkova, Zh.V. Vecherkina, E.A. Lescheva

Voronezh N.N. Burdenko State Medical University, Voronezh, Russia

*Correspondence: A.N. Morozov, The Department of Hospital Dentistry, Voronezh N.N. Burdenko State Medical University, Voronezh, Russia, Avenue of Revolution 14, 394036 Voronezh, Russia; e-mail: micvsma@yandex.ru

**Keywords:** Periodontal diseases, Prevention, Oral rinses

**Abstract**

The search for available and effective products for inflammatory periodontal disease prevention is an important objective in preventive dentistry.

From a great variety of oral rinses, we chose the most commonly used 0.05% chlorhexidine solution and the new preparation Dentaseptin, due to the significance of their use for periodontal disease prevention.

We examined 50 patients aged 40–50 years; an unsatisfactory oral hygiene index (more than 1.7) was observed in 70%, which indicates the presence of inflammatory periodontal disease. The patients were divided into two groups who brushed their teeth twice a day with individually selected toothbrush and toothpaste. Twenty patients from the control group used chlorhexidine as an oral rinse, and 30 patients from the study group used Dentaseptin for oral cavity rinsing. The results in the study group showed 30% improvement in oral health compared to the control patients (*p*<0.01), which demonstrates its positive effects and opens the possibility for recommending it for wide use in dentistry.

**Prevention of the risk of complications in patients with cardiovascular disease using anesthesia during an outpatient visit to the dentist**

Anna V. Podoprigora^1^, Olga A. Kumirova^2^, Natalia S. Moiseeva^2^ *, Alexandr S. Scherbinin^1^

Voronezh N.N. Burdenko State Medical University, Voronezh, Russia

^1^Department of oral and maxillofacial surgery

^2^ Department of Hospital Dentistry

*Correspondence: PhD., Natalia S. Moiseeva, The Department of Hospital Dentistry, Voronezh N.N. Burdenko State Medical University, Voronezh, Russia, Avenue of Revolution 14, 394036 Voronezh, Russia; e-mail: natazarova@yandex.ru

**Keywords:** Preventive measures, Anesthesia complications, Cardiovascular diseases

**Abstract**

Nowadays there is a high demand for local anesthesia during a dental patient visit. The follow-up rate of patients with cardiovascular disease is one-third of the number applying for dental care (J. A. Baart, H. S. Brand, 2010, 2015). Prevention of the risk of complications in these patients with the use of high-tech anesthetics is a priority for every dental doctor.

At the dental departments of Voronezh State Medical University, 100 patients aged 45–70 years were examined. Among these patients, 53% applied for treatment of complicated forms of caries and 47% applied for teeth removal due to complications of chronic periodontitis. The analysis of medical histories of patients and their clinical records, as well as advisory expert opinions, revealed the presence of cardiovascular disease. In 86% of cases, the examined had different cardiovascular diseases or their combinations; 79% had hemodynamic disorders due to atherosclerosis of the heart and brain vessels. As pain causes a significant increase in the amount of adrenaline released during dental procedures, patients with ischemic heart disease and essential hypertension need adequate anesthesia.

Therefore, this category of patients was recommended for use of adrenaline only at a dilution of 1:100.000 and 1:200.000 with restriction of its quantity. Thus, the elimination of cardiovascular complications is explained by an individualized approach to the choice of anesthetic during anesthesia, which will allow for effective dental treatment. Keeping the oral cavity in good condition plays a key role in general well-being and quality of life.

**Informativeness of epidemiological indicators as the basis of prevention effectiveness**

Irina A. Belenova^1^ *, Elena A. Andreeva^1^, Inessa V. Koretskaya^2^, Elena Yu. Caverina^3^

Voronezh N.N. Burdenko State Medical University, Voronezh, Russia

^1^ Department of Hospital Dentistry

^2^ Propaedeutic Dentistry Department

*Correspondence: Dr.Med.Sc., Irina A. Belenova, The Department of Hospital Dentistry, Voronezh N.N. Burdenko State Medical University, Voronezh, Russia, Avenue of Revolution 14, 394036 Voronezh, Russia; e-mail: vrnvgma@mail.ru

**Keywords:** Oral health indicators, Prevention programs, Children’s dental health

**Abstract**

In Russia, the principle criterion for children’s dental health is traditionally considered the DMFT (total number of decayed, missing and filled teeth) index. This criterion does not allow identification of individual risk factors for dental health and, even more importantly, determining the directions of individualized prevention of dental diseases.

The present research evaluates the informativeness of a number of the European Community Health Indicators of dental health among 116 13-year-old schoolchildren from Voronezh, according to the following criteria: the percentage of healthy children, the average value of the DMFT index and its components, the prevalence of gingival bleeding, and the oral hygiene index, as well as a number of subjective criteria related to behavioral habits.

The objective indicators (dental status data) revealed the need for preventive and/or non-emergency treatment in 93% and the need for emergency dental treatment in 11%, among many others. The subjective indicators (questionnaire survey data) revealed the ineffectiveness of preventive measures in more than 53% of respondents.

European indicators of dental health have made it possible to assess the main negative objective and subjective trends in society and to develop mass and personalized preventive and hygienic measures [1, 2].

**References:**

1. Leus P.A., Denga O.V., Kalbaev A.A. et al. Monitoring of Children’s Dental Health with the Use of the European Community Health Indicators**.** Dent Art. 2013; 4 (73). 63-69.
2. Kunin A.A, Belenova I.A., Ippolitov Y.A, Moiseeva N.S, Kunin D.A. Predictive research methods of enamel and dentine for initial caries detection. EPMA J. 201326;4(1):19. doi: 10.1186/1878-5085-4-19.

**Preventive effectiveness of the cyto-bacterioscopic method of investigation in the detection of periodontal disease**

Aleksandr Shcherbakov*, V.V. Sudareva, I. Shcherbakova

Voronezh N.N. Burdenko State Medical University, Voronezh, Russia

*Correspondence: Dr., Aleksandr Shcherbakov, Voronezh N.N. Burdenko State Medical University, Voronezh, Russia, Avenue of Revolution 14, 394036 Voronezh, Russia; e-mail: micvsma@yandex.ru

**Keywords:** Periodontal diseases, Different markers, Preventive therapy, *F. alocis*, *P. gingivalis*

**Abstract**

The primary detection and diagnosis of periodontal disease using conventional diagnostic methods is not informative, does not reveal features of the pathological process, and does not predict the outcome of therapy based on etiology and pathogenesis. The gingival crevicular fluid is highly informative in a study focused on searching for methods for the early diagnosis and detection of periodontal disease. In our study, data of various parameters of the gingival crevicular fluid and its correlation with the development of pathological processes in the periodontium are summarised. Previously, we studied 120 subjects of both genders aged 18–25 years and analyzed data of cytological and bacterioscopic investigations, public health research methods and physical examination, and investigation of multiple enzymes in the gingival crevicular fluid. The results revealed that at the early stages of inflammation in periodontal tissue, the cocci population increases and forms clusters, mature pseudomycelium of yeasts increases up to 8–10 colonies in the field of view, and immature epithelial cells with nuclear-to-cytoplasmic ratio of 1:2 appear. Recent studies have shown that *F. alocis* has high potential for the early diagnosis and detection of periodontal disease. This is due to the fact that *F. alocis* and *P. gingivalis* are able to generate mixed colonies and coexist. For example, enzymes of *F. alocis* can induce *P. gingivalis* to reproduce and spread, enhancing its virulence. Our preliminary data collected in a study consisting of 45 subjects have shown interesting findings in cytology and bacterioscopy. An increase in the number of *F. alocis* and *P. gingivalis* led to an increase in streptococci and fungi, and vice versa. All of the above constitutes evidence that cytology and bacterioscopy are highly informative methods for early detection of periodontal disease.

**Application of the Vector System with ozonized water for secondary prevention of inflammatory periodontal diseases**

Anatoly A. Kunin*, Kristina P. Kubyshkina

Voronezh N.N. Burdenko State Medical University, Voronezh, Russia

*Correspondence: Dr.Med.Sc., Anatoly A. Kunin, Voronezh N.N. Burdenko State Medical University, Voronezh, Russia, Avenue of Revolution 14, 394036 Voronezh, Russia; e-mail: kunin36@gmail.com

**Keywords:** Prevention of inflammatory periodontal diseases, Dental calculus, Biofilm, Vector System, Ozonized water

**Abstract**

The main reason for tooth loss is inflammatory periodontal disease. That is why early diagnostics, timely prevention and complex treatment are necessary to relieve the inflammatory process in order to recover or to achieve sustained remission within a short time. Treatment should start with the removal of dental calculus, plaque and biofilms from the teeth surfaces. The Vector System (Durr-Dental, Germany) and ultrasonic scalers are used for this. According to a number of authors, however, its bactericidal effect is not enough to achieve sustained long-term remission. Ozone therapy is considered an effective method of treatment of inflammatory diseases. However, ozone has only a superficial antimicrobial action.

Thus the purpose of our research was to assess the efficiency of complex treatment of inflammatory periodontal disease using the Vector System with ozonized water.

We examined 151 patients, who were divided into two groups: the main group (91 people) and the control group (60 people). The control patients received traditional treatment, and the main group received vector therapy with ozonized water. Relief from inflammation was achieved in a shorter time (on the second day) in the main group, with treatment time reduced to half; 96% of these patients experienced remission for 1 year, whereas 4% of patients had only primary signs of inflammation. The patients inf the control group experienced mild inflammation on the seventh day, and remission lasted for 6 months in 43% and for 3 months in 57% of patients.

The advantage of this method of periodontal disease treatment compared to traditional treatment has been proven by clinical, bacterioscopic and radiological indicators.

**The use of fluoride-free toothpastes in prophylaxis of dental caries**

Anatoly A. Kunin, Tatyana Kupets, Natalia S. Moiseeva*, Dmitry A. Kunin

Voronezh N.N. Burdenko State Medical University, Russia

*Correspondence: PhD., Natalia S., Voronezh N.N. Burdenko State Medical University, Voronezh, Russia, Avenue of Revolution 14, 394036 Voronezh, Russia; e-mail: natazarova@yandex.ru

**Keywords:** Caries prevention, High-tech methods, Fluoride-free toothpastes

**Abstract**

For 25 years we have been publishing research papers confirming the anti-caries effect of fluoride in persons who have not reached a particular age (15.5 years) [1]. Unfortunately, at present, toothpastes with high fluoride content are being promoted for both adults and children.

More than 150 patients between the ages of 20 and 40 were placed under our observation, all of whom used the fluoride-free ROCS brand toothpaste to maintain their oral hygiene. The control group comprised over 100 patients who used toothpastes containing fluoride. The results were measured using electron microscopy, and microchemical and clinical methods. The reduction in caries was 84.5% in the study group and only 62.3% in the control group (*p*<0.05). Electron and atomic force microscopy studies in the first group showed that magnesium contained in the toothpaste was actively integrated into the hydroxyapatite crystal cells in the enamel. The fluoride that was present in the control group toothpaste was not detected on the chemical spectrum of the enamel, but nevertheless had a strong bactericidal effect.

The studies showed that the presence of magnesium inside the enamel structure increased from 1.5 to 2.7 μmol/L (*p*<0.05) in adult patients, with only trace amounts of fluoride found (0.1), confirming the fact that toothpastes containing magnesium play an active role in strengthening enamel crystal cells.

As such, electron and atomic force microscopy studies with microchemical spectrum determination have confirmed the caries-resistant effect (clinical) that occurs when using fluoride-free toothpaste.

**Reference**

1. Kunin АА, Belenova IA, Ippolitov YaA, Moiseeva NS, Kunin DA. Predictive research methods of enamel and dentine for initial caries detection. EPMA J. 2013;4(1):19. doi:10.1186/1878-5085-4-19.

